# Acetaldehyde in the Enders triple cascade reaction via acetaldehyde dimethyl acetal

**DOI:** 10.3762/bjoc.19.92

**Published:** 2023-08-24

**Authors:** Alessandro Brusa, Debora Iapadre, Maria Edith Casacchia, Alessio Carioscia, Giuliana Giorgianni, Giandomenico Magagnano, Fabio Pesciaioli, Armando Carlone

**Affiliations:** 1 Department of Physical and Chemical Sciences, Università degli Studi dell’Aquila, via Vetoio, 67100, L’Aquila, Italy; 2 IUSS Scuola Universitaria Superiore di Pavia, Palazzo del Broletto, Piazza della Vittoria, 15, 27100, Pavia, Italy; 3 INSTM, Consorzio Nazionale per la Scienza e Tecnologia dei Materiali, RU L’Aquila, Italy

**Keywords:** acetaldehyde, acetaldehyde dimethyl acetal, cascade reaction, multicomponent reaction, organocatalysis

## Abstract

Asymmetric organocatalyzed multicomponent reactions represent an important toolbox in the field of organic synthesis to build complex scaffolds starting from simple starting materials. The Enders three-component cascade reaction was a cornerstone in the field and a plethora of organocatalyzed cascade reactions followed. However, acetaldehyde was not shown as a successful reaction partner, probably because of its high reactivity. Herein, we report the Enders-type cascade reaction using acetaldehyde dimethyl acetal, as a masked form of acetaldehyde. This strategy directly converts acetaldehyde, nitroalkenes and enals into stereochemically dense cyclohexenals in good yield and excellent enantioselectivity.

## Introduction

Multicomponent reactions (MCRs) are chemical processes that involve three or more compounds, in which the product contains all the atoms of the reagents, except for condensation coproducts, such as water, hydrogen chloride or other small molecules [1–3]. MCRs have a great advantage over the classical two-component reactions; they allow the construction of complex molecular motifs in only one synthetic operational step starting from simpler building blocks. For this reason, the use of MCRs is appealing in the construction of natural or synthetic products [2–5] or libraries of compounds [2], and is generally considered an advantage in organic synthesis for atom economy, waste reduction and time saving. Cascade reactions are defined as chemical processes in which two or more bond-forming steps happen under identical reaction conditions, and where a subsequent transformation takes place at the functionality obtained in the former bond-forming event. Cascade reactions are valuable tools for streamlining the synthesis of structurally complex molecules in a single operation and from readily available substrates. Their combination with asymmetric aminocatalysis [4,6–8] has recently led to innovative approaches for the one-step enantioselective preparation of stereochemically dense molecules. Nowadays, organocatalytic cascade processes provide a powerful tool for achieving molecular complexity. Their synthetic potential has been demonstrated by their application in the total synthesis of complex natural compounds [2,4,9–12].

**Scheme 1:**
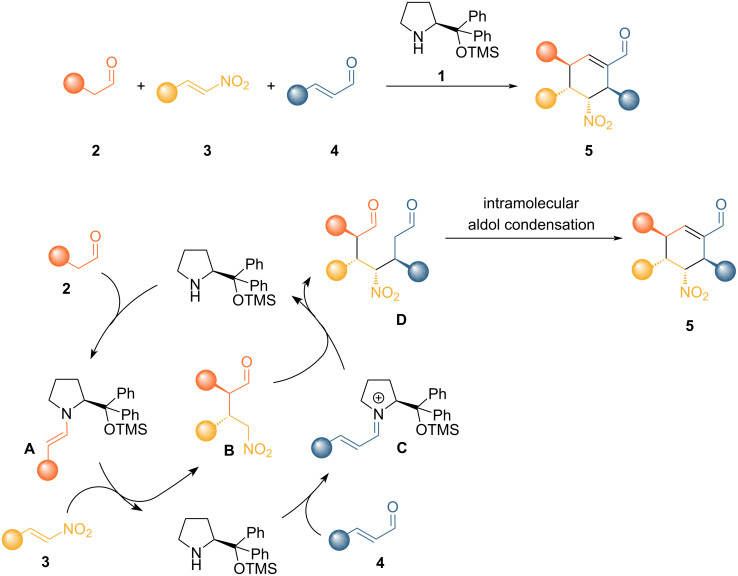
Original triple organocatalytic cascade reaction developed by Enders.

A remarkable example of an amino-catalyzed cascade process was reported by Enders [11], a three-component cascade reaction for the synthesis of polyfunctionalized cyclohexenes bearing multiple stereocenters. The reaction is promoted by a chiral secondary amine, which is capable of catalyzing each step of the process activating the substrates through enamine and iminium ion catalysis towards a Michael/Michael/aldol process. The ingenious crafting of the reaction lies in the selection of the reactivity of the different nucleophiles and electrophiles present in the mixture, both as reagents and as intermediates. First, the chiral aminocatalyst **1** activates the saturated aldehyde **2** via enamine intermediate **A**, which intercepts the nitroalkene **3** in a Michael-type addition forming intermediate **B**. Hydrolysis regenerates catalyst **1** that can then selectively condense with the α,β-unsaturated aldehyde **4** to form chiral iminium ion intermediate **C**. Iminium ion **C** reacts with intermediate **B** in a further Michael-type reaction. The last step involves the enamine intermediate which drives an intramolecular aldol condensation to form the final product **5**. In this elegant cascade process, catalyst **1** promotes three consecutive carbon–carbon bond forming steps generating four stereogenic centers with high diastereoselectivity and complete enantiocontrol (Scheme 1).

This elegantly designed example established a new direction in asymmetric aminocatalysis, leading to an impressive growth of methods based on organocascade processes [8,10,13–16]. The experimental simplicity of the strategy offers the potential of rapidly increasing structural and stereochemical complexity starting from readily available substrates. The scope of the process was shown to be successful with aliphatic aldehydes **2**, aromatic nitroalkenes **3**, and both aromatic and aliphatic unsaturated aldehydes **4**.

Given our recent interest in the use of a surrogate of acetaldehyde [17–19] to address the challenges of working with free acetaldehyde, we wondered why the scope of this reaction did not include acetaldehyde and questioned whether a three-component triple cascade would indeed work employing this highly reactive substrate. The use of acetaldehyde as a reagent has always been challenging. The low boiling point and high volatility pose a problem with its handling and safety. The small steric hindrance gives rise to a high reactivity both as an electrophile and as a pro-nucleophile, hampering chemoselectivity (further to side reactions such as self-aldol condensations, polymerization and Tishchenko-type processes) and stereoselectivity [20]; the activation of acetaldehyde via aminocatalysis, furthermore, suffers from a lack of proper steric hindrance for the enantio-discrimination process. However, some methodologies enabling the use of acetaldehyde have been reported [20–24].

The safety and handling problems associated with acetaldehyde can be solved by synthetic equivalents that can be generated in situ through different paths. Some examples are represented by vinyl acetate [25], silyl vinyl ethers [26], ethanol, pyruvic acid, (*E*)-3-chloroacrylic acid, 2,4,6-trimethyl-1,3,5-trioxane (paraldehyde) [24,27], and acetaldehyde dimethyl acetal (**6**) [17–19]. On the basis of a long-term project based on masked reagents, our group has previously demonstrated the feasibility of the addition of a masked acetaldehyde **6** to nitroalkene derivatives with low reagent excess and high enantioselectivity; this reaction represents the first step in the Enders triple cascade catalytic cycle.

**Figure 1:**
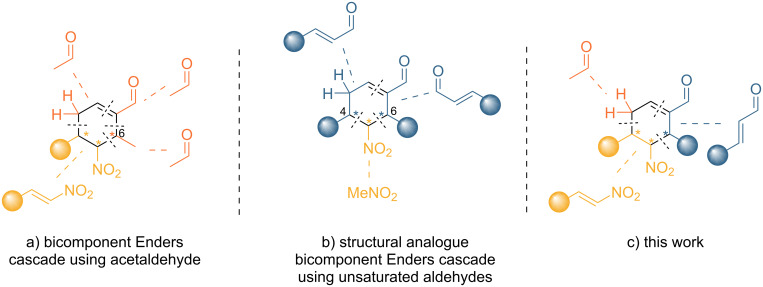
Approaches based on the original Enders cascade reaction to access trisubstituted cyclohexene carbaldehyde derivatives.

The use of acetaldehyde in a two-component cascade reaction was previously reported by Enders [27]; however, the scope of this reaction is limited to cyclohexene carbaldehydes bearing a methyl group on the C-6 atom (Figure 1a). On the other hand, these structural motifs can also be synthesized via the condensation of two equivalents of an enal and nitromethane (Figure 1b), although in this case C-4 and C-6 present the same substituent [28]. Interestingly, to the best of our knowledge, the use of acetaldehyde in the original Enders triple cascade reaction has not been explored despite its synthetic value. Indeed, this protocol would enable a wide structural variability in the synthesis of 3-substituted cyclohexene carbaldehydes (Figure 1c).

## Results and Discussion

In order to explore the feasibility of the triple cascade reaction with acetaldehyde (**2a**) as a substrate, we tested the original reaction conditions reported by Enders using *trans*-β-nitrostyrene (**3**) and *trans*-cinnamaldehyde (**4**) as the other substrates (Table 1, entry 1). The reaction proved to yield the desired product, indicating that the catalytic system may indeed be applicable. Lowering the amount of organocatalyst **1** to 10 mol % (Table 1, entry 2) resulted in a decrease of both yield and selectivity.

**Table 1:** Enders reaction with acetaldehyde.^a^

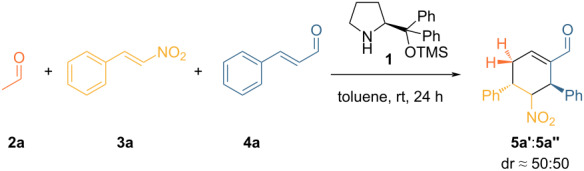					

Entry	**1** (mol %)	ee^b^ (%)	Conversion^c^ (%)	Yield^c^ (%)	Selectivity^d^ (%)

1	20	99	91	43	48
2	10	99	95	33	35

^a^Reaction conditions: **3** (0.5 mmol, 1 equiv), **2a** (0.6 mmol, 1.2 equiv), **4** (0.525 mmol, 1.05 equiv) were added to a solution of **1** (0.05 mmol, 0.1 equiv) in toluene (0.625 M wrt limiting reagent) and allowed to stir for 24 hours at room temperature. ^b^Determined by chiral HPLC analysis. ^c^Calculated by ^1^H NMR using triphenylmethane as an internal standard. ^d^Ratio between yield and conversion.

**Scheme 2:**
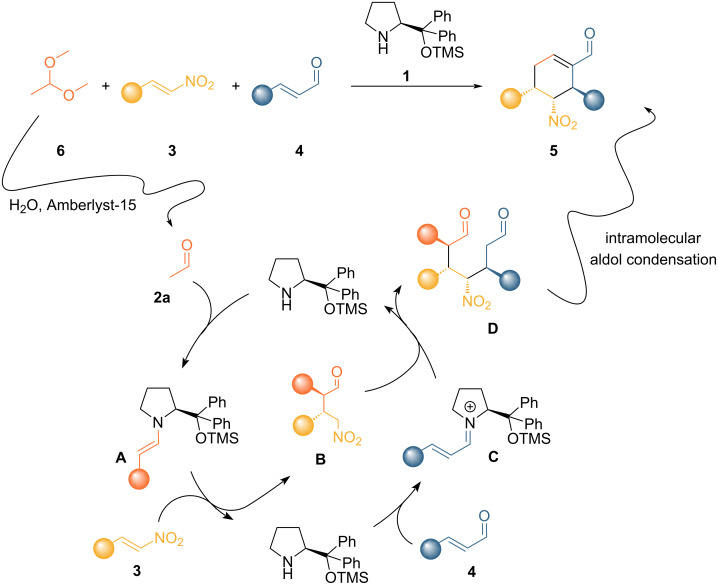
Acetaldehyde dimethyl acetal (**6**) as an acetaldehyde surrogate to effect a triple organocatalytic cascade reaction.

Based on the results obtained (Table 1, entry 2) and the reaction conditions developed in our previous work [17], we tried to introduce **6** as an acetaldehyde equivalent, adding water in the reaction system and Amberlyst-15 as a catalyst to accelerate the hydrolysis process (Scheme 2).

To our delight, **6** as an acetaldehyde surrogate allows a slightly better yield with doubled selectivity, measured as the ratio between yield and conversion (Table 2, entry 1). This means that the productivity of the reaction using **6** is superior, since far more substrate was converted into the desired product. A solvent screening (see Supporting Information File 1) did not reveal any better alternative to toluene. The use of chloroform (Table 2, entry 2), as in our previous report [17], showed an improvement on selectivity, however, toluene was chosen as a more benign solvent. After further optimization of the acidic resin, stoichiometry, concentration, temperature, and reaction time (see Supporting Information File 1), we identified the best reaction conditions that yield the desired product in 44% yield, over 48 h (Table 2, entry 6). Unfortunately, side products and unwanted reactions do not allow to have a higher yield. All reactions provide the product in >99% ee, which is expected for a process which involves two consecutives stereoselective reactions. The control of the diastereomeric ratio is, however, difficult to attain as demonstrated by an extensive screening and is always near 50:50 without significant deviations changing the reaction conditions. On the contrary, the use of aldehydes other than acetaldehyde generates higher control [11]. It was previously shown that the first stereogenic center formed in the presented cascade process is formed with high control [17]. Therefore, the second carbon–carbon bond forming step, i.e., the organocatalyzed Michael addition of the nitronate to the α,β-unsaturated iminium ion, should be tackled to improve the diastereocontrol. All efforts to discriminate the two faces of the nitroenolate during the addition proved unproductive during the optimization, therefore, a potential epimerization was envisaged during the course of the reaction, but this hypothesis was discarded after further experimentation; submitting the two isolated diastereomers to the reaction conditions did not show any change. A slight improvement in the dr was found by exposing the mixture of the diastereomers to a strong basic environment (*t*-BuOK/MeOH) to force the formation of secondary nitronates. However, it could not be improved to synthetically interesting values. The observed diastereomeric ratio is, therefore, the direct result of the second Michael addition reaction, as previously reported [29], and no post-process epimerization event could be found.

**Table 2:** Selected optimization reaction conditions.^a^

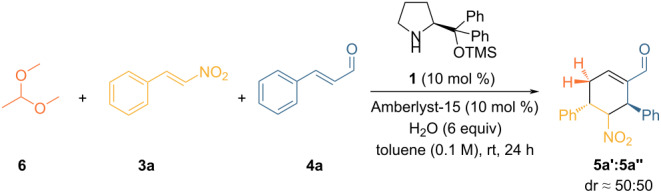					

Entry	Deviation from above	ee^b^ (%)	Conversion^c^ (%)	Yield^c^ (%)	Selectivity^d^ (%)

1	none	99	61	40	66
2	CHCl_3_	99	54	43	79
3	0.25 M	99	72	35	48
4	0.5 M	99	93	39	42
5	1 M	99	88	35	40
6	0.25 M, 48 h	99	79	44	56

^a^Reaction conditions: **3** (0.1 mmol, 1 equiv), **6** (0.2 mmol, 2 equiv), **4** (0.1 mmol, 1.05 equiv), H_2_O (0.6 mmol, 6 equiv), and Amberlyst-15 (10 mol %) were added to a solution of **1** (10 mol %) in 1 mL of solvent (0.1 M) and allowed to stir for 24 hours at room temperature. ^b^Determined by chiral HPLC analysis. ^c^Calculated by ^1^H NMR using triphenylmethane as an internal standard. ^d^Ratio between yield and conversion.

**Figure 2:**
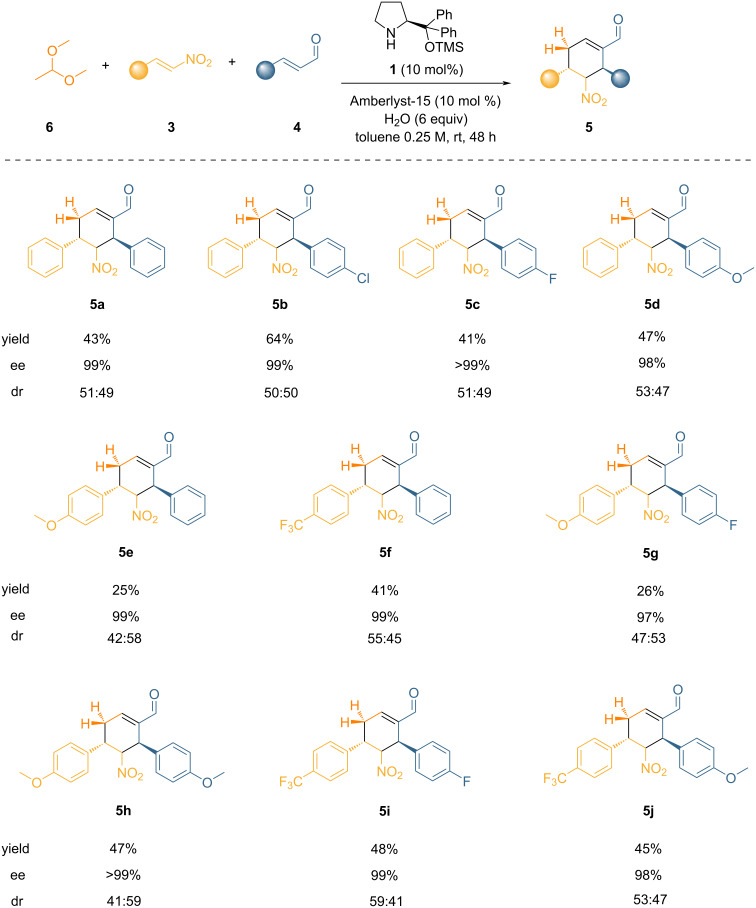
Scope of the cascade reaction using **6** as an acetaldehyde equivalent. Reaction conditions: **3** (0.5 mmol, 1 equiv), **6** (1 mmol, 2 equiv), **4** (0.525 mmol, 1.05 equiv), H_2_O (3.0 mmol, 6 equiv), and Amberlyst-15 (10 mol %) were added to a solution of **1** (10 mol %) in 2 mL of solvent (0.25 M) and allowed to stir for 48 hours at room temperature. Yields of isolated compounds are given. Diastereomeric ratio (dr) determined by ^1^H NMR analysis. Enatiomeric excess (ee) determined by chiral HPLC analysis.

With the best conditions in our hand, we evaluated the scope of our triple cascade reaction enabled by masked acetaldehyde. As highlighted in Figure 2, the reaction proceeded smoothly regardless of structural and electronic variations of both substrates, giving access to a variety of complex cyclohexenals. Although the scope is limited to aromatic nitroalkenes and enals, the yields are good considering the complexity of the domino process that involves highly reactive partners, and the ees are consistently very high. Finally, albeit there is low diastereocontrol, the diastereomers can be easily separated by simple flash chromatography.

## Conclusion

An unprecedented methodology for the synthesis of 4,6-disubstituted 5-nitrocyclohexene carbaldehydes with three contiguous stereogenic centers using acetaldehyde as one of the reaction components of an Enders cascade reaction has been developed. The masked form of acetaldehyde, which is hydrolyzed in situ using Amberlyst-15 as an acid catalyst, instead of directly using acetaldehyde allows for higher yields and fewer byproducts. Using mild reaction conditions, it was possible to obtain a variety of functionalized cyclohexene carbaldehydes in good yields and very high enantiomeric excesses. Unfortunately, the developed methodology is currently limited to aromatic substrates and the formation of one stereocenter is difficult to control, leading to a mixture of two diastereomers. Current efforts in our laboratories are addressing these challenges.

## Supporting Information

File 1 Experimental part, NMR and HPLC spectra.
