# Supplementary material for: Acetaldehyde in the Enders triple cascade reaction via acetaldehyde dimethyl acetal
Source: Beilstein J Org Chem. 2023 Aug 24;19:1243–50. doi: 10.3762/bjoc.19.92 (PMC10477997; doi:10.3762/bjoc.19.92)
Supplement: File 1 — Experimental part, NMR and HPLC spectra. [file Beilstein_J_Org_Chem-19-1243-s001.pdf]

BEILSTEIN JOURNAL OF ORGANIC CHEMISTRY

Supporting Information

for

Acetaldehyde in the Enders triple cascade reaction via acetaldehyde dimethyl acetal

Alessandro Brusa, Debora Iapadre, Maria Edith Casacchia, Alessio Carioscia, Giuliana Giorgianni, Giandomenico Magagnano, Fabio Pesciaioli and Armando Carlone

Beilstein J. Org. Chem. **2023,** *19,* 1243–1250. doi:10.3762/bjoc.19.92

Experimental part, NMR and HPLC spectra

License and Terms: This is a supporting information file under the terms of the Creative Commons Attribution License (https://creativecommons.org/licenses/by/4.0). Please note that the reuse, redistribution and reproduction in particular requires that the author(s) and source are credited and that individual graphics may be subject to special legal provisions.
The license is subject to the *Beilstein Journal of Organic Chemistry* terms and conditions: (https://www.beilstein-journals.org/bjoc/terms)

Table of Contents

1. General information S1
1.1 Instrumentations S1
1.2 Materials and Methods... S2
2. Experimental procedures S3
2.1 Optimization reactions S3
2.2 General procedure S4
2.3 General procedure for racemic compound preparation.. S5
3. NMR Spectra S55
4. HPLC Spectra……………………………………………………………………………S35
5. References ……………………………………………………………………………S55

1. General information

1.1 Instrumentations

Nuclear magnetic resonance analyses (^{1}H NMR spectra) were acquired using a Bruker Advance III 400 MHz spectrophotometer. Chemical shifts (δ) are reported in ppm relative to residual solvent signals for ^{1}H NMR (^{1}H NMR: 7.26 ppm for $CDCl_3$). Coupling constants are given in Hz. Due to the congestion in the products, in some cases additional peaks and line broadenings are observed due to rotameric species. ^{1}H NMR yields were measured by analysing the reaction mixture using triphenylmethane as an internal standard. Chromatographic purifications of compounds **5a–j** were performed using automated Biotage® Isolera LS Systems. HPLC analyses were acquired using an Agilent 1220 Infinity II liquid chromatographer equipped with Phenomenex columns Lux 3 µm i-Cellulose 5, 100 × 4.6 mm. HPLC analysis was performed on the aldehyde products and it often revealed a set of two peaks for each enantiomer, probably due to the formation of hemiacetal/acetal derivatives. Therefore, the evaluation of the enantiomeric excess was performed by integrating both peaks; the retention time was arbitrarily reported with respect to the first

peak of the set for each compound. Reduction to the alcohol with $NaBH_4$ was attempted but it afforded a complex mixture of products, with probably also a likely epimerisation on the carbon α to the nitro group. Optical rotations were measured on a ZUZI 412 Digital Polarimeter (tube length: 100 mm). Exact masses were measured on hybrid quadrupole-TOF high resolution (HRMS) mass spectrometer (Xevo G2 QTOF, Waters, Manchester, UK) using a Z-spray-ESI interface operating in positive ionization mode with resolution of the TOF mass spectrometer about 20,000 at full width half maximum (FWHM).

1.2 Materials and Methods

Acetaldehyde dimethyl acetal (**6**), *trans*-β-nitrostyrene (**3a**), (*S*)/(*R*)-(–)-α,α-diphenyl-2-pyrrolidinemethanol trimethylsilyl ether (**1**), Amberlyst-15 hydrogen form (Amberlyst-15), *trans*-cinnamaldehyde (**4a**) were purchased from Merck, aldehydes **4b–d** were purchased from Fluorochem and used as received unless otherwise stated. Silica Gel 60A (35–70 μ), HPLC solvents and analytical grade solvents were purchased from Merck. Nitroalkenes **3b**,**c** were prepared as previously reported [1]. All nitroalkenes and aldehydes used as shown below.

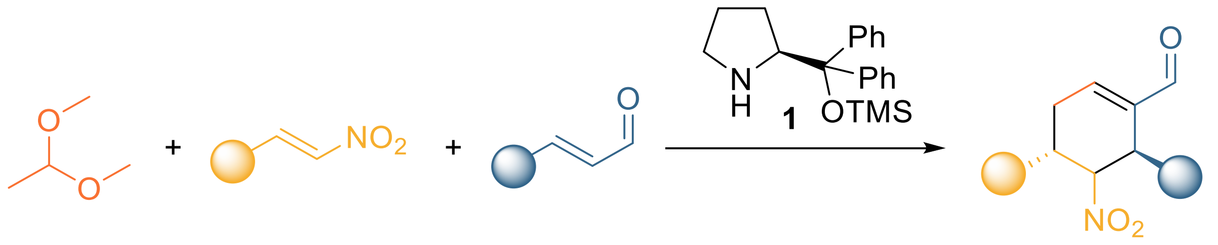

6 **3** **4** **5**

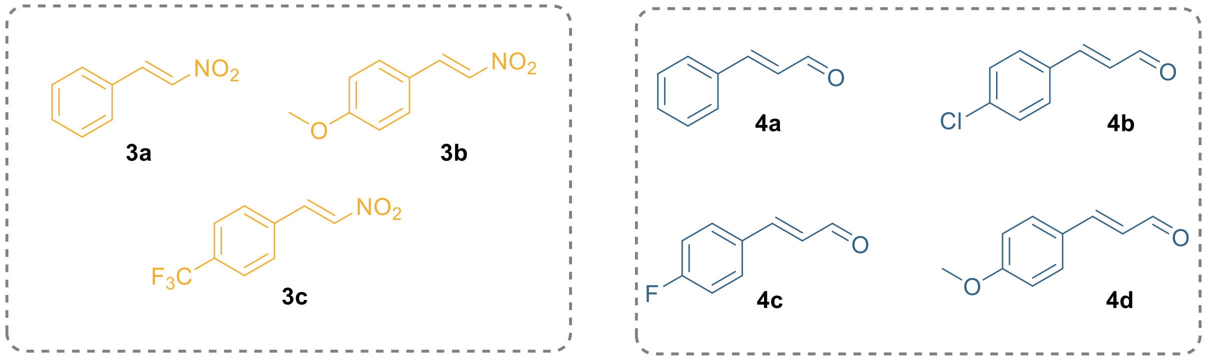

2. Experimental procedures

2.1 Optimization reactions

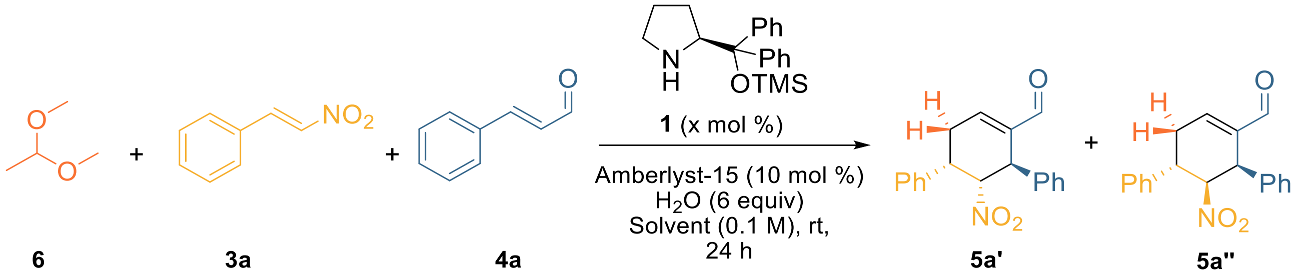

Table 1: Optimization reactions

Entry	6 (eq.)	4a (eq.)	1 (eq.)	Solvent	Conversion (%)	Selectivity (%)	NMR Yield (%)	*dr* (5a' : 5a'')
1	1.2	1.5	0.1	Chloroform	53	62	33	58:42
2	2	1.05	0.1	Chloroform	54	79	43	52:48
3	2	1.05	0.2	Chloroform	45	44	20	60:40
4	2	1.05	0.1	Acetonitrile	43	12	5	50:50
5	2	1.05	0.1	Ethyl acetate	77	23	18	50:50
6	2	1.05	0.1	Tetrahydrofuran	79	37	29	44:56
7	2	1.05	0.1	Cyclohexane	58	43	25	44:56
**8**	**2**	**1.05**	**0.1**	**Toluene**	**61**	**66**	**40**	**50:50**
9 [a]	2	1.05	0.1	Toluene	62	37	23	44:56
10 [b]	2	1.05	0.1	Toluene	59	50	30	47:53
11	2	2.1	0.1	Toluene	70	48	33	44:56
12 [c]	2	1.05	0.1	Toluene	72	47	34	41:59
13 [d]	2	1.05	0.1	Toluene	58	45	26	37:63
14 [e]	2	1.05	0.1	Toluene	71	50	36	44:56
15 [f]	2	1.05	0.1	Toluene	75	48	36	44:56

[a] 20% Amberlyst-15; [b] 5% Amberlyst-15; [c] Reaction performed at 40 °C; [d] Reaction performed at 60 °C; [e] 10% Alumina added; [f] 10% Amberlite-45 IR added.

Entries 1, 2 and *3* demonstrated that **6** performs similarly to acetaldehyde in this reaction. As expected, all reactions gave high enantiomeric excesses since the process involves two consecutive enantioselective steps. Therefore, different solvents have been screened and toluene gave the best results in terms of selectivity and conversion (*entry 8*). Additionally, different acidic resins, temperatures, and additives were screened. Nevertheless, the best results were given by *entry 8* and a further concentration-time optimisation of these conditions has been carried out, as shown in Table 2.

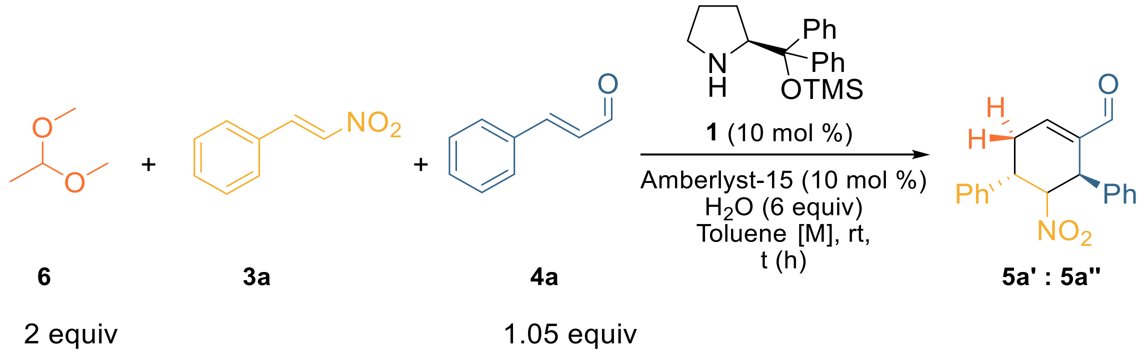

Table 2: Concentration-time optimization.

Entry	Concentration (M)	Time (h)	Conversion (%)	Selectivity (%)	NMR Yield (%)	*dr* (5a' : 5a'')
1	0.05	24	36	59	21	44:56
2	0.25	24	72	48	35	52:48
3	0.5	24	93	42	39	55:45
4	1	24	88	40	35	57:43
5	0.05	48	63	39	25	44:56
**6[a]**	**0.25**	**48**	**79**	**56**	**44**	**52:48**
7	0.5	48	77	48	37	55:45
8	1	48	93	44	41	55:45

[a] Enantiomeric excess 99%, measured as the average of the two diastereomers

Starting from the good outcomes obtained from entry 8 (Table 1), different concentrations and reaction times have been screened. *Entry 6* gave the best results in terms of yield and selectivity and these conditions were employed to perform the reaction scope.

2.2 General procedure

Acetaldehyde dimethyl acetal (**6**) (2 equiv) and a *trans*-cinnamaldehyde derivative (**4**) (0.525 mmol 1.05 equiv) were added to a 4 mL scintillation vial equipped with a magnetic bar containing a solution of (*S*)-diphenyltrimethylsiloxymethylpyrrolidine (**1**) (16.3 mg, 0.05 mmol, 0.1 equiv), nitroalkene (**3**) (0.5 mmol, 1 equiv), Amberlyst-15 (10.6 mg, 4.7 mmol/g, 0.1 equiv), water (54 µL, 3 mmol, 6 equiv) and toluene (2 mL). The reaction mixture was left stirring at room temperature for 48 h. Amberlyst-15 resin was filtered off using a cotton plug and the solvent was evaporated under reduced pressure. The crude was purified by flash chromatography on SiO_2 using a mixture of petroleum ether/ethyl acetate to yield the desired products. The absolute configuration of **5a** was assigned by comparison with the literature [2]. The absolute configuration of other products was assigned by analogy, considering a uniform mechanism of stereoinduction.

2.3 General procedure for racemic compound preparation

The racemic mixture used for the measurement of the enantiomeric excess were prepared “artificially”, setting-up a parallel reaction with the other enantiomer of the catalyst. The corresponding products were purified and used as they were for the HPLC analysis.

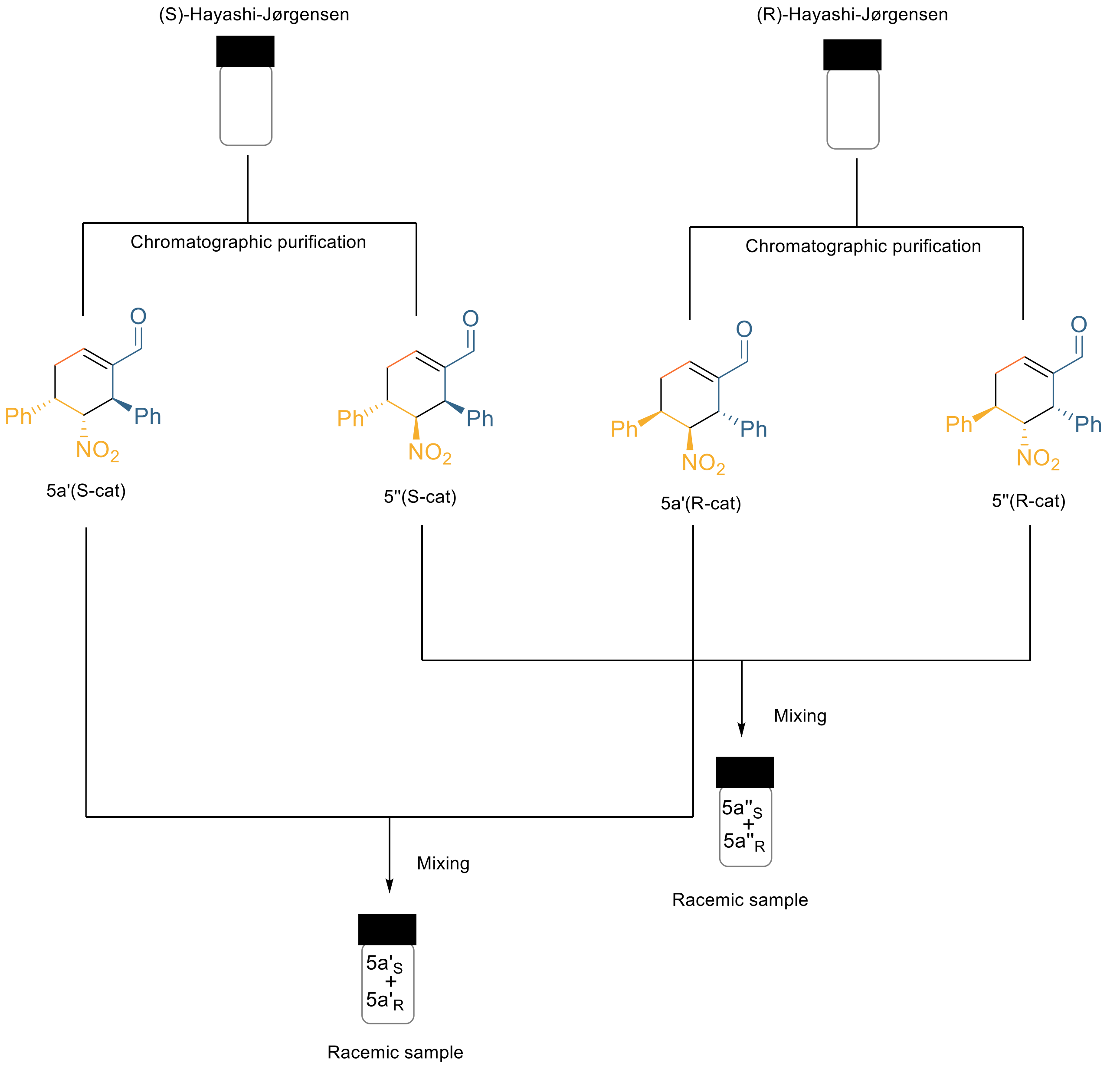

Scheme 1: Process to obtain the racemates avoiding the formation of undesired diastereomers.

This atypical preparation of the racemic samples was done to avoid the formation of undesired diastereomers using an equimolar mixture of both the enantiomers of the catalyst; being a multistep one-pot reaction it is not possible to guarantee that a chiral intermediate generated by one enantiomer of the catalyst will not do the second step using the other enantiomer of the catalyst.

(1'S,2'R,3'R)-2'-Nitro-1',2',3',6'-tetrahydro-[1,1':3',1''-terphenyl]-4'-carbaldehyde ***5a'*** [2]

The desired product was obtained as an oil (24.3 mg, 0.0791 mmol, yield = 15.8%, ee >99%).

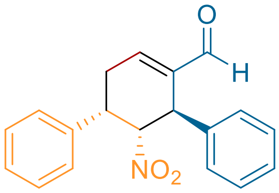

^{1}H-NMR (400 MHz, $CDCl_3$, 303 K) δ 9.53 (s, 1H), 7.35 – 7.11 (m, 10H), 5.33 – 5.26 (m, 1H), 4.73 (d, J = 4.8 Hz, 1H), 3.64 (m, 1H), 3.12 (m, 1H), 2.67 (m, 1H).. ^{13}C NMR (101 MHz, $CDCl_3$, 303 K) δ 190.7, 148.0, 140.1, 135.2, 129.0, 128.7, 128.6, 128.4, 127.7, 127.2, 89.2, 42.6, 37.8, 35.4, 29.7.HPLC (Lux 3µm i-Cellulose 5, 100 X 4.6 mm, Hexane/i-Propanol 80:20, flow: 0.5 mL/min, λ=210 nm), t_{minor}: 17.8 min; t_{major}: 24.2 min. $[\alpha]_D^{25}$ = -111.6 (c = 1.9·10^{-3} g/mL, $CHCl_3$). All analytical data were in agreement with reported data.[2]

(1'S,2'S,3'R)-2'-Nitro-1',2',3',6'-tetrahydro-[1,1':3',1''-terphenyl]-4'-carbaldehyde ***5a''***

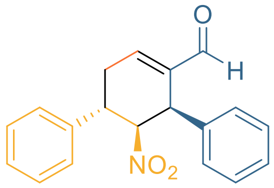

The desired product was obtained as an oil (42.1 mg, 0.137 mmol, yield = 27.4%, ee >99%). ^{1}H-NMR (400 MHz, $CDCl_3$, 303 K) δ 9.53 (s, 1H), 7.27 – 7.05 (m, 11H), 5.26 (dd, *J* = 12.5, 5.9 Hz, 1H), 4.71 (d, *J* = 5.6 Hz, 1H), 3.61 (td, *J* = 11.5, 6.8 Hz, 1H), 3.17 – 3.07 (m, 1H), 2.66 (dd, *J* = 20.7, 10.4 Hz, 1H). ^{13}C NMR (101 MHz, $CDCl_3$, 303 K) δ 190.8, 148.1, 140.2, 140.2, 135.3, 129.13, 128.8, 128.7, 128.6, 127.8, 127.3, 89.4, 42.8, 37.9, 35.5. HPLC (Lux 3µm i-Cellulose 5, 100 X 4.6 mm, Hexane/i-Propanol 80:20, flow: 0.5 mL/min, λ=210 nm), t_{minor}: 20.9 min; t_{major}: 21.5 min. $[\alpha]_D^{25}$ = -124.0 (c = 3.4·10^{-3} g/mL, $CHCl_3$).

(1'S,2'R,3'R)-4''-Chloro-2'-nitro-1',2',3',6'-tetrahydro-[1,1':3',1''-terphenyl]-4'-carbaldehyde ***5b'***

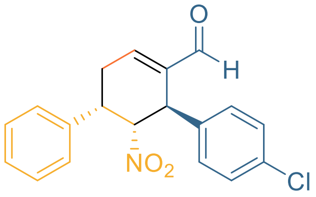

The desired product was obtained as an oil (43.2 mg, 0.126 mmol, yield = 25.3 %, ee >99%). ^{1}H NMR (400 MHz, $CDCl_3$, 303 K) δ 9.59 (s, 1H), 7.57 – 6.97 (m, 10H), 5.33 (m, 1H), 4.78 (m, 1H), 3.82 – 3.46 (m, 1H), 3.18 (m, 1H), 2.84 – 2.63 (m, 1H). ^{13}C NMR (101 MHz, $CDCl_3$, 303 K) δ 190.7, 148.5, 147.6, 147.4, 140.2, 139.9, 139.8, 138.7, 135.1, 134.6, 133.9, 133.6, 129.9, 129.4, 129.3, 129.2, 129.1, 128.9, 128.8, 128.7, 128.7, 128.7, 128.6, 128.5, 127.9, 127.7, 127.3, 127.2, 89.2, 89.2, 77.2, 42.7, 42.3, 42.1, 37.9, 37.4, 35.4, 35.3, 35.1. HPLC (Lux 3µm i-Cellulose 5, 100 X 4.6 mm, Hexane/i-Propanol 80:20, flow: 0.5 mL/min, λ=210 nm), t_{minor}: 15.4 min; t_{major}: 22.2 min. $[\alpha]_D^{25}$ = -135.6 (c = 1.87·10^{-3} g/mL, $CHCl_3$). ESI-MS: $C_{19}H_{16}ClNO_3Na$ [M+Na]+ calcd.: 364.0716, found: 364.0717.

(1'S,2'S,3'R)-4''-Chloro-2'-nitro-1',2',3',6'-tetrahydro-[1,1':3',1''-terphenyl]-4'-carbaldehyde ***5b***''

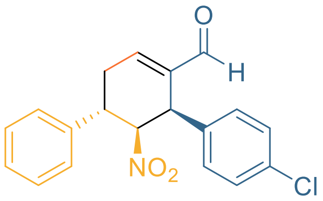

The desired product was obtained as an oil (66.7 mg, 0.195 mmol, yield = 39%, ee= 98%). ^{1}H-NMR (400 MHz, $CDCl_3$, 303 K) δ 9.52 (s, 1H), 7.24 (m, 8H), 7.05 – 6.97 (m, 1H), 6.94 (d, J = 8.6 Hz, 1H), 4.87 (m, 1H), 4.47 (d, J = 22.4 Hz, 1H), 3.37 – 3.09 (m, 2H), 2.92 – 2.77 (m, 1H).. ^{13}C NMR (101 MHz, $CDCl_3$, 303 K) δ 207.1, 191.6, 150.7, 150.3, 149.9, 138.8, 138.2, 138.0, 137.8, 137.5, 136.6, 134.1, 134.0, 129.5, 129.5, 129.4, 129.2, 129.2, 129.1, 128.9, 128.8, 128.2, 128.2, 128.1, 127.9, 127.4, 127.4, 91.1, 91.1, 43.2, 42.5, 40.0, 38.0, 37.5, 36.8, 28.0, 27.9. HPLC (Lux 3µm i-Cellulose 5, 100 X 4.6 mm, Hexane/i-Propanol 80:20, flow: 0.5 mL/min, λ=210 nm), t_{minor}: 56.8min; t_{major}: 26.8 min. $[\alpha]_D^{25}$ = -194.8 (c = $0.87 \cdot 10^{-3}$ g/mL, $CHCl_3$). ESI-MS: $C_{19}H_{16}ClNO_3Na$ [M+Na]+ calcd.: 364.0716, found: 364.0775.

(1'S,2'R,3'R)-4''-Fluoro-2'-nitro-1',2',3',6'-tetrahydro-[1,1':3',1''-terphenyl]-4'-carbaldehyde ***5c'***

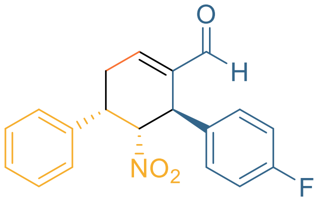

The desired product was obtained as an oil (31.4 mg, 0.0965 mmol, yield = 19.3%, ee >99%). ^{1}H NMR (400 MHz, $CDCl_3$, 303 K) δ 9.57 (s, 1H), 7.39 – 7.05 (m, 10H), 5.42 – 5.22 (m, 1H), 4.77 (m, 1H), 3.75 – 3.53 (m, 1H), 3.16 (dt, J = 10.8, 5.0 Hz, 1H), 2.81 – 2.54 (m, 1H). ^{13}C NMR (101 MHz, $CDCl_3$, 303 K) δ 190.8, 164.1, 163.4, 161.6, 160.9, 148.3, 147.8, 140.1, 139.9, 135.8, 135.2, 131.2, 130.3, 130.2, 129.1, 129.0, 128.9, 128.8, 128.7, 128.6, 127.8, 127.3, 116.1, 115.9, 115.7, 89.5, 89.2, 42.7, 41.9, 37.8, 37.2, 35.4, 30.9, 29.8. HPLC (Lux 3µm i-Cellulose 5, 100 X 4.6 mm, Hexane/i-Propanol 80:20, flow: 0.5 mL/min, λ=210 nm), t_{minor}: 14.4 min; t_{major}: 19.3 min. $[\alpha]_D^{25}$ = -113.2 (c = $2.2 \cdot 10^{-3}$ g/mL, $CHCl_3$). ESI-MS: $C_{19}H_{16}FNO_3Na$ [M+Na]+ calcd.: 348.1012, found: 348.1056.

(1'S,2'S,3'R)-4''-Fluoro-2'-nitro-1',2',3',6'-tetrahydro-[1,1':3',1''-terphenyl]-4'-carbaldehyde ***5c***''

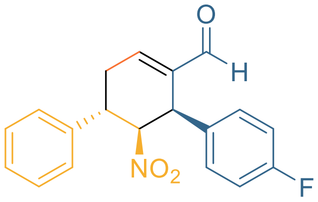

The desired product was obtained as an oil (34.8 mg, 0.107 mmol, yield = 21.4 %, ee= 96%). ^{1}H NMR (400 MHz, $CDCl_3$, 303 K) δ 9.59 (s, 1H), 7.49 – 6.77 (m, 10H), 4.92 (s, 1H), 4.53 (d, J = 11.6 Hz, 1H), 3.79 (m, 1H), 3.29 (m, 1H), 2.89 (m, 1H).. ^{13}C NMR (101 MHz, $CDCl_3$, 303 K) δ 191.7, 163.7, 163.7, 161.3, 161.2, 150.5, 149.9, 138.9, 138.34, 137.9, 134.8, 134.8, 133.9, 133.8, 129.8, 129.7, 129.4, 129.1, 129.1, 128.2, 128.2, 128.1, 127.4,

116.4, 116.2, 116.1, 115.9, 91.4, 91.3, 91.3, 43.2, 42.5, 38.3, 37.4, 36.8, 31.4, 29.8, 28.2, 28.1. HPLC (Lux 3µm i-Cellulose 5, 100 X 4.6 mm, Hexane/i-Propanol 80:20, flow: 0.5 mL/min, λ=210 nm), t_{minor}: 28,6 min; t_{major}: 46,6 min. $[\alpha]_D^{25}$ = -193.5 (c = 3.7·10^{-3} g/mL, $CHCl_3$). ESI-MS: $C_{19}H_{16}FNO_3Na$ [M+Na]+ calcd.: 348.1012, found: 348.1012.

(1'S,2'R,3'R)-4''-Methoxy-2'-nitro-1',2',3',6'-tetrahydro-[1,1':3',1''-terphenyl]-4'-carbaldehyde **5d'**

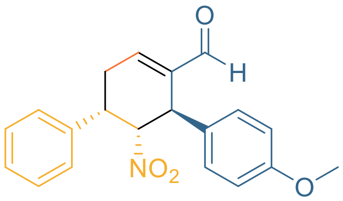

The desired product was obtained as an oil (58.5 mg, 0.173 mmol, yield = 34.7%, ee= 99%). ^{1}H NMR (400 MHz, $CDCl_3$, 303 K) δ 9.56 (s, 1H), 7.39 – 7.19 (m, 5H), 7.14 (d, J = 8.8 Hz, 1H), 7.07 – 7.02 (m, 1H), 6.95 (d, J = 8.8 Hz, 1H), 6.89 (d, J = 8.8 Hz, 1H), 6.80 (d, J = 8.8 Hz, 1H), 4.92 (m, 1H), 4.49 (d, J = 9.2 Hz, 1H), 3.77 (two s, rotamers, 3H), 3.42 – 3.31 (m, 1H), 3.30 – 3.14 (m, 1H), 2.94 – 2.81 (m, 1H).. ^{13}C NMR (101 MHz, $CDCl_3$, 303 K) δ 198.9, 191.8, 191.8, 191.7, 159.4, 159.3, 150.5, 150.2, 139.0, 138.4, 138.3, 138.2, 138.2, 130.9, 129.9, 129.3, 129.3, 129.2, 129.0, 128.5, 128.2, 128.1, 128.1, 127.5, 127.4, 114.7, 114.4, 91.6, 91.5, 79.5, 55.4, 55.4, 53.5, 46.5, 43.2, 42.5, 38.1, 37.3, 36.7, 29.8, 28.3, 27.9. HPLC (Lux 3µm i-Cellulose 5, 100 X 4.6 mm, Hexane/i-Propanol 80:20, flow: 0.5 mL/min, λ=210 nm), t_{minor}: 24.9 min; t_{major}: 31.4 min. $[\alpha]_D^{25}$ = -109.2 (c = 3.5·10^{-3} g/mL, $CHCl_3$). ESI-MS: $C_{20}H_{19}NO_4Na$ [M+Na]+ calcd.: 360.1212, found: 360.1212.

(1'R,2'R,3'S)-4''-Methoxy-2'-nitro-1',2',3',6'-tetrahydro-[1,1':3',1''-terphenyl]-4'-carbaldehyde **5d''**

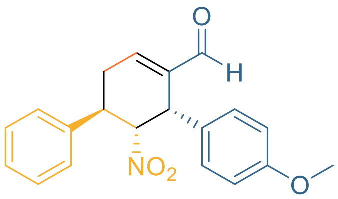

The desired product was obtained, using (*R*)-(**1**), as an oil (21 mg, 0.0622 mmol, yield = 12.4%, ee= >99% (*R*)-(**1**)). ^{1}H NMR (400 MHz, $CDCl_3$, 303 K) δ 9.50 (s, 1H), 7.29 (m, 3H), 7.25 – 7.16 (m, 2H), 7.13 – 6.99 (m, 4H), 6.82 (m, 1H), 5.27 – 5.16 (m, 1H), 4.67 (m, 1H), 3.76 (two s, rotamers, 3H), 3.57 (m, 1H), 3.08 (m, 1H), 2.63 (m, 1H). ^{13}C NMR (101 MHz, $CDCl_3$, 303 K) δ 190.9, 190.9, 159.8, 159.1, 148.3, 147.8, 140.4, 140.3, 140.2, 135.4, 131.9, 129.8, 129.1, 128.8, 128.7, 128.5, 128.4, 127.7, 127.3, 127.2, 114.7, 114.5, 114.3, 89.7, 89.4, 55.5, 55.4, 55.3, 42.8, 42.1, 37.9, 37.1, 35.5, 35.5. HPLC (Lux 3µm i-Cellulose 5, 100 X 4.6 mm, Hexane/i-Propanol 80:20, flow: 0.5 mL/min, λ=210 nm), t_{minor}: 66.5 min; t_{major}: 65.1 min. $[\alpha]_D^{25}$ = +304.3 (c = 1.7·10^{-3} g/mL, $CHCl_3$). ESI-MS: $C_{20}H_{19}NO_4Na$ [M+Na]+ calcd.: 360.1212, found: 360.1207.

(1'S,2'R,3'R)-4-Methoxy-2'-nitro-1',2',3',6'-tetrahydro-[1,1':3',1''-terphenyl]-4'-carbaldehyde ***5e'***

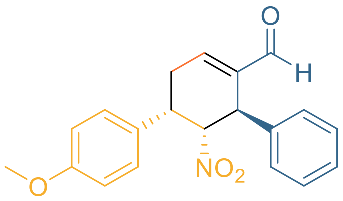

The desired product was obtained as an oil (24 mg, 0.0711 mmol, yield = 14.2%, ee >99%). ^{1}H NMR (400 MHz, $CDCl_3$, 303 K) δ 9.51 (s, 1H), 7.39 – 6.66 (m, 10H), 4.86 (m, 1H), 4.43 (d, J = 9.3 Hz, 1H), 3.72 (two s, rotamers, 3H), 3.36 – 3.07 (m, 2H), 2.82 (m, 1H).. ^{13}C NMR (101 MHz, $CDCl_3$, 303 K) δ 191.8, 191.8, 159.4, 159.4, 150.5, 150.2, 139.0, 138.5, 138.3, 138.2, 130.9, 129.9, 129.3, 129.2, 129.1, 128.5, 128.5, 128.1, 128.1, 127.4, 114.7, 114.4, 91.6, 91.5, 55.4, 55.4, 55.4, 43.2, 42.6, 37.3, 37.3, 36.7, 28.3, 27.9. HPLC (Lux 3µm i-Cellulose 5, 100 X 4.6 mm, Hexane/i-Propanol 80:20, flow: 0.5 mL/min, λ=210 nm), t_{minor}: 24.4 min; t_{major}: 30.9 min. $[\alpha]_D^{25}$ = -165.7 (c = 1.33·10^{-3} g/mL, $CHCl_3$). ESI-MS: $C_{20}H_{19}NO_4Na$ [M+Na]+ calcd.: 360.1212, found: 360.1213.

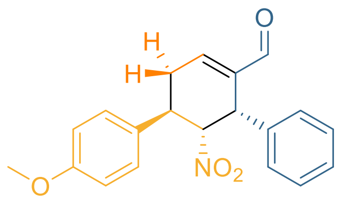

(1'R,2'R,3'S)-4-Methoxy-2'-nitro-1',2',3',6'-tetrahydro-[1,1':3',1''-terphenyl]-4'-carbaldehyde ***5e''*** The desired product was obtained, using (*R*)-(**1**), as an oil (17.5 mg, 0.0519 mmol, yield = 10.4%, ee >99%, (*R*)-(**1**)). ^{1}H NMR (400 MHz, $CDCl_3$, 303 K) δ 9.57 (s, 1H), 7.50 – 6.80 (m, 10H), 5.38 – 5.20 (m, 1H), 4.75 (d, J = 5.9 Hz, 1H), 3.83 (two s, rotamers, 3H), 3.68 – 3.51 (m, 1H), 3.15 (m, 1H), 2.78 – 2.60 (m, 1H). ^{13}C NMR (101 MHz, $CDCl_3$, 303 K) δ 190.9, 190.9, 159.8, 159.1, 148.3, 148.3, 147.8, 140.2, 135.4, 131.9, 129.8, 129.1, 128.8, 128.7, 128.5, 128.4, 127.7, 127.3, 114.5, 114.3, 114.3, 89.7, 89.4, 55.4, 55.3, 42.8, 42.1, 37.9, 37.1, 35.5. HPLC (Lux 3µm i-Cellulose 5, 100 X 4.6 mm, Hexane/i-Propanol 80:20, flow: 0.5 mL/min, λ=210 nm), t_{minor}: 46.2 min; t_{major}: 36.5 min. $[\alpha]_D^{25}$ = +195.6 (c = 0.57·10^{-3} g/mL, $CHCl_3$). ESI-MS: $C_{20}H_{19}NO_4Na$ [M+Na]+ calcd.: 360.1212, found: 360.1319.

(1'R,2'S,3'S)-2'-Nitro-4-(trifluoromethyl)-1',2',3',6'-tetrahydro-[1,1':3',1''-terphenyl]-4'-carbaldehyde **5f'**

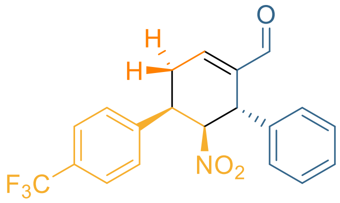

The desired product was obtained, using (*R*)-(**1**), as an oil (42.7 mg, 0.114 mmol, yield = 22.8%, ee >99%, (*R*)-(**1**)). ^{1}H NMR (400 MHz, $CDCl_3$, 303 K) δ 9.60 (s, 1H), 7.65 (d, J = 8.4 Hz, 1H), 7.55 (d, J = 8.4 Hz, 1H), 7.44 – 7.28 (m, 5H), 7.25 – 7.16 (m, 2H), 7.10 – 7.02 (m, 1H), 4.96 (m, 1H), 4.59 (m, 1H), 3.52 – 3.20 (m, 2H), 3.01 – 2.86 (m, 1H). ^{13}C NMR (101 MHz, $CDCl_3$, 303 K) δ 191.6, 151.0, 149.5, 143.0, 138.7, 138.3, 137.9, 137.6, 129.5, 129.2, 128.6, 128.4, 128.3, 128.1, 127.9, 127.4, 126.5, 126.4, 126.4, 126.3, 126.1,

126.0, 90.9, 43.3, 42.9, 37.7, 37.2, 29.8, 28.1, 27.8. HPLC (Lux 3µm i-Cellulose 5, 100 X 4.6 mm, Hexane/i-Propanol 80:20, flow: 0.5 mL/min, λ=210 nm), t_{minor}: 21.7 min; t_{major}: 10.4 min. $[\alpha]_D^{25}$ = +92.4 (c = 2.95·10^{-3} g/mL, $CHCl_3$). ESI-MS: $C_{20}H_{16}F_3NO_3Na$ [M+Na]+ calcd.: 398.0980, found: 398.1027.

(1'S,2'S,3'R)-2'-Nitro-4-(trifluoromethyl)-1',2',3',6'-tetrahydro-[1,1':3',1''-terphenyl]-4'-carbaldehyde ***5f''***

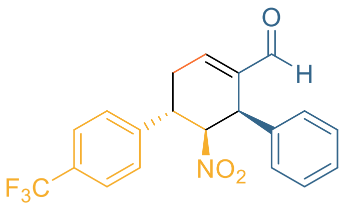

The desired product was obtained as an oil (34.1 mg, 0.0908 mmol, yield = 18.2%, ee= 97%; please note that at the λ= 254nm ratio signal/noise is too low and only λ= 210 nm has been used to calculate ee). ^{1}H NMR (400 MHz, $CDCl_3$, 303 K) δ 9.60 (s, 1H), 7.65 (d, J = 8.3 Hz, 2H), 7.55 (d, J = 8.6 Hz, 1H), 7.30 (m, 6H), 7.09 – 7.02 (m, 1H), 4.94 (m, 1H), 4.57 (m, 1H), 3.40 – 3.22 (m, 2H), 2.96 (m, 1H).. ^{13}C NMR (101 MHz, $CDCl_3$, 303 K) δ 191.5, 150.9, 143.0, 137.9, 137.6, 129.5, 129.2, 128.6, 128.4, 127.4, 126.4, 90.9, 42.9, 37.7, 29.9, 28.1. HPLC (Lux 3µm i-Cellulose 5, 100 X 4.6 mm, Hexane/i-Propanol 80:20, flow: 0.5 mL/min, λ=210 nm), t_{minor}: 18.2 min; t_{major}: 21.0 min. $[\alpha]_D^{25}$ = -160.4 (c = 5.95·10^{-3} g/mL, $CHCl_3$). ESI-MS: $C_{20}H_{16}F_3NO_3Na$ [M+Na]+ calcd.: 398.0980, found: 398.0980.

(1'S,2'R,3'R)-4''-Fluoro-4-methoxy-2'-nitro-1',2',3',6'-tetrahydro-[1,1':3',1''-terphenyl]-4'-carbaldehyde **5g'**

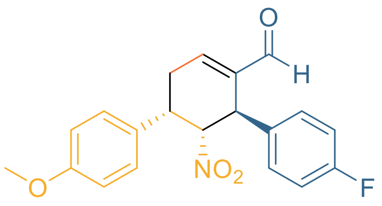

The desired product was obtained as an oil (18.9 mg, 0.0532 mmol, yield = 10.6%, ee >99%). ^{1}H NMR (400 MHz, $CDCl_3$, 303 K) δ 9.50 (s, 1H), 7.12 – 6.93 (m, 7H), 6.83 (m, 2H), 5.17 (m, 1H), 4.66 (d, J = 7.2 Hz, 1H), 3.76 (two s, rotamers, 3H), 3.63 – 3.44 (m, 1H), 3.14 – 2.99 (m, 1H), 2.70 – 2.57 (m, 1H). ^{13}C NMR (101 MHz, $CDCl_3$, 303 K) δ 190.8, 190.8, 164.1, 163.4, 161.7, 159.8, 159.2, 148.4, 147.4, 140.4, 140.1, 131.7, 131.3, 131.3, 130.3, 130.3, 129.8, 129.0, 128.9, 128.4, 116.1, 115.9, 115.9, 115.7, 115.5, 114.6, 114.3, 114.3, 89.6, 89.5, 55.4, 55.3, 42.1, 42.0, 37.3, 37.1, 35.4. HPLC (Lux 3µm i-Cellulose 5, 100 X 4.6 mm, Hexane/i-Propanol 80:20, flow: 0.5 mL/min, λ=210 nm), t_{minor}: 19.8 min; t_{major}: 28.9 min. $[\alpha]_D^{25}$ = -99.5 (c = 2.95·10^{-3} g/mL, $CHCl_3$). ESI-MS: $C_{20}H_{18}FNO_4Na$ [M+Na]+ calcd.: 378.1118, found: 378.1118.

(1'S,2'S,3'R)-4''-Fluoro-4-methoxy-2'-nitro-1',2',3',6'-tetrahydro-[1,1':3',1''-terphenyl]-4'-carbaldehyde ***5g''***

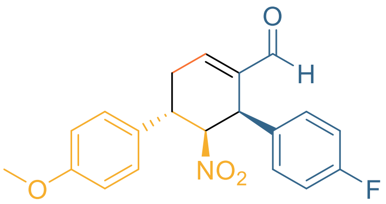

The desired product was obtained as an oil (27 mg, 0.076 mmol, yield = 15.2%, ee >99%). ^{1}H NMR (400 MHz, $CDCl_3$, 303 K) δ 9.57 (s, 1H), 7.42 – 6.73 (m, 9H), 4.97 – 4.78 (m, 1H), 4.48 (s, 1H), 3.79 (two s, rotamers, 3H), 3.37 – 3.28 (m, 1H), 3.27 – 3.15 (m, 1H), 2.88 (m, 1H). ^{13}C NMR (101 MHz, $CDCl_3$, 303 K) δ 191.7, 163.7, 161.2, 159.5, 150.7, 149.7, 138.5, 138.3, 134.9, 134.8, 130.8, 129.8, 129.8, 129.2, 129.1, 128.5, 128.4, 116.4, 116.2, 116.1, 115.9, 114.8, 114.6, 114.5, 91.5, 91.5, 55.5, 55.4, 42.5, 42.4, 36.8, 36.7, 28.4, 28.2. HPLC (Lux 3µm i-Cellulose 5, 100 X 4.6 mm, Hexane/i-Propanol 80:20, flow: 0.5 mL/min, λ=210 nm), t_{minor}: 28.2 min; t_{major}: 29.4 min. $[\alpha]_D^{25}$ = -150.9 (c = 1.88·10^{-3} g/mL, $CHCl_3$). ESI-MS: $C_{20}H_{18}FNO_4Na$ [M+Na]+ calcd.: 378.1118, found: 378.1131.

(1'R,2'S,3'S)-4,4''-Dimethoxy-2'-nitro-1',2',3',6'-tetrahydro-[1,1':3',1''-terphenyl]-4'-carbaldehyde ***5h'***

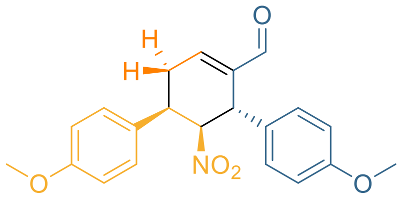

The desired product was obtained, using (*R*)-(**1**), as an oil (39.9 mg, 0.109 mmol, yield = 21.7%, ee >99%, (*R*)-(**1**)). ^{1}H NMR (400 MHz, $CDCl_3$, 303 K) δ 9.56 (s, 1H), 7.14 (d, J = 8.7 Hz, 3H), 6.98 (d, J = 8.8 Hz, 2H), 6.89 (d, J = 8.8 Hz, 2H), 6.82 (d, J = 12.0 Hz, 2H), 4.89 (s, 1H), 4.46 (s, 1H), 3.78 (d, J = 16.3 Hz, 7H), 3.41 – 3.29 (m, 1H), 3.19 (dd, J = 20.2, 11.1 Hz, 1H), 2.92 – 2.73 (m, 1H). ^{13}C NMR (101 MHz, $CDCl_3$, 303 K) δ 199.1, 191.9, 159.4, 159.3, 159.3, 150.3, 138.4, 132.1, 130.9, 130.1, 130.1, 129.7, 129.2, 128.6, 128.5, 127.9, 114.7, 114.5, 114.4, 114.2, 91.7, 79.8, 60.5, 55.4, 55.4, 55.3, 55.3, 46.6, 42.5, 37.5, 36.6, 29.8, 28.3, 14.3. HPLC (Lux 3µm i-Cellulose 5, 100 X 4.6 mm, Hexane/i-Propanol 80:20, flow: 0.5 mL/min, λ=210 nm), t_{minor}: 58.0 min; t_{major}: 43.6 min. $[\alpha]_D^{25}$ = +188.8 (c = 2.31·10^{-3} g/mL, $CHCl_3$). ESI-MS: $C_{21}H_{21}NO_5Na$ [M+Na]+ calcd.: 390.1317, found: 390.1334.

(1'R,2'R,3'S)-4,4''-Dimethoxy-2'-nitro-1',2',3',6'-tetrahydro-[1,1':3',1''-terphenyl]-4'-carbaldehyde ***5h''***

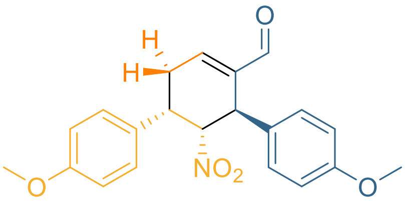

The desired product was obtained, using (*R*)-(**1**), as an oil (45.5 mg, 0.124 mmol, yield = 24.8%, ee >99%, (*R*)-(**1**)). ^{1}H NMR (400 MHz, $CDCl_3$, 303 K) δ 9.48 (s, 1H), 7.10 (d, J = 8.8 Hz, 2H), 7.01 (d, J = 8.8 Hz, 2H), 6.82 (m, 5H), 5.16 (dd, J = 12.3, 5.7 Hz, 1H), 4.63 (d, J = 4.9 Hz, 1H), 3.75 (two s, 6H), 3.58 – 3.44 (m, 1H), 3.10 – 2.98 (m, 1H), 2.59 (m, 1H).. ^{13}C NMR (101 MHz, $CDCl_3$, 303 K) δ 190.9, 190.6, 159.7, 159.1, 147.9, 140.3, 132.1, 129.7, 128.4, 127.3, 114.7, 114.5, 114.4, 114.2, 114.1, 89.7, 55.3, 55.3, 42.1, 37.1, 35.5, 29.8. HPLC (Lux 3µm i-Cellulose 5, 100 X 4.6 mm, Hexane/i-Propanol 80:20, flow: 0.5 mL/min, λ=210 nm), t_{minor}: 59.9 min; t_{major}: 47.4 min. $[\alpha]_D^{25}$ = +221.5 (c = 3.96·10^{-3} g/mL, $CHCl_3$). ESI-MS: $C_{21}H_{21}NO_5Na$ [M+Na]+ calcd.: 390.1317, found: 390.1314.

(1'R,2'S,3'S)-4''-Fluoro-2'-nitro-4-(trifluoromethyl)-1',2',3',6'-tetrahydro-[1,1':3',1''-terphenyl]-4'-carbaldehyde **5i'**

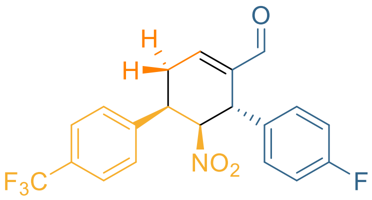

The desired product was obtained, using (*R*)-(**1**), as an oil (45.2 mg, 0.115 mmol, yield = 23%, ee= 99%, (*R*)-(**1**)). ^{1}H-NMR (400 MHz, $CDCl_3$, 303 K) δ 9.58 (s, 1H), 7.65 (d, J = 7.9 Hz, 1H), 7.57 (d, J = 8.0 Hz, 1H), 7.44 – 7.33 (m, 2H), 7.21 (m, 2H), 7.13 – 6.95 (m, 3H). 4.92 (d, *J* = 9.1 Hz, 1H), 4.57 (s, 1H), 3.47 – 3.33 (m, 1H), 3.32 – 3.19 (m, 1H), 2.95 (dt, *J* = 20.1, 5.2 Hz, 1H). ^{13}C NMR (101 MHz, $CDCl_3$, 303 K) δ 190.5, 148.3, 147.3, 140.3, 139.8, 139.4, 135.3, 130.8, 130.4, 130.3, 129.1, 129.0, 128.9, 128.1, 127.8, 126.2, 125.9, 125.9, 116.4, 116.2, 116.1, 115.9, 89.3, 88.9, 42.4, 41.9, 37.9, 37.4, 35.4, 35.1, 32.1, 29.9, 14.3. HPLC (Lux 3µm i-Cellulose 5, 100 X 4.6 mm, Hexane/i-Propanol 80:20, flow: 0.5 mL/min, λ=210 nm), t_{minor}: 14.4 min; t_{major}: 10.3 min. $[\alpha]_D^{25}$ = +67.2 (c = 4.1·10^{-3} g/mL, $CHCl_3$. ESI-MS: $C_{20}H_{15}F_4NO_3Na$ [M+Na]+ calcd.: 416.0886, found: 416.0940.

(1'R,2'R,3'S)-4''-Ffluoro-2'-nitro-4-(trifluoromethyl)-1',2',3',6'-tetrahydro-[1,1':3',1''-terphenyl]-4'-carbaldehyde **5i''**

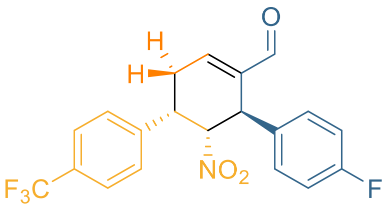

The desired product was obtained, using (*R*)-(**1**), as an oil (48.3 mg, 0.123 mmol, yield = 24.6%, ee> 99%, (*R*)-(**1**)). ^{1}H NMR (400 MHz, $CDCl_3$, 303 K) δ 9.59 (s, 1H), 7.65 (d, J = 8.3 Hz, 1H), 7.57 (d, J = 8.3 Hz, 1H), 7.43 – 7.33 (m, 2H), 7.25 – 7.17 (m, 2H), 7.12 – 6.97 (m, 3H), 4.91 (m, 1H), 4.56 (s, 1H), 3.52 – 3.17 (m, 2H), 2.94 (dt,

J = 20.2, 5.5 Hz, 1H). ^{13}C NMR (101 MHz, $CDCl_3$, 303 K) δ 191.4, 150.6, 149.6, 141.9, 138.3, 137.9, 134.5, 129.8, 129.7, 129.2, 129.1, 128.6, 127.9, 126.4, 126.1, 126.1, 116.6, 116.4, 116.3, 116.1, 90.9, 90.8, 42.9, 42.5, 37.2, 37.1, 29.9, 28.3, 27.8. HPLC (Lux 3µm i-Cellulose 5, 100 X 4.6 mm, Hexane/i-Propanol 80:20, flow: 0.5 mL/min, λ=210 nm), t_{minor}: 15.1 min; t_{major}: 7.9 min. $[\alpha]_D^{25}$ = -104.8 (c = $4.4 \cdot 10^{-3}$ g/mL, $CHCl_3$). ESI-MS: $C_{20}H_{15}F_4NO_3Na$ [M+Na]+ calcd.: 416.0886, found: 416.0886.

(1'R,2'S,3'S)-4''-Methoxy-2'-nitro-4-(trifluoromethyl)-1',2',3',6'-tetrahydro-[1,1':3',1''-terphenyl]-4'-carbaldehyde **5j'**

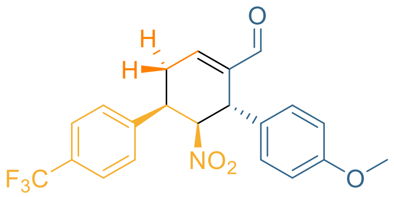

The desired product was obtained, using (*R*)-(**1**), as an oil (53.4 mg, 0.132 mmol, yield = 26.3%, ee= 99%, (*R*)-(**1**)). ^{1}H NMR (400 MHz, $CDCl_3$, 303 K) δ 9.59 (s, 1H), 7.64 (d, J = 8.3 Hz, 1H), 7.56 (d, J = 8.4 Hz, 1H), 7.46 – 7.30 (m, 3H), 7.18 (m, 1H), 6.97 (d, J = 8.8 Hz, 1H), 6.91 (d, J = 8.8 Hz, 1H), 6.82 (d, J = 8.8 Hz, 1H), 4.96 – 4.86 (m, 1H), 4.54 (s, 1H), 3.79 (two s, rotamers, 3H), 3.38 – 3.17 (m, 2H), 3.00 – 2.83 (m, 1H).. ^{13}C NMR (101 MHz, $CDCl_3$, 303 K) δ 191.6, 159.6, 151.1, 149.2, 143.11, 137.9, 129.4, 129.2, 128.6, 128.5, 127.9, 126.4, 126.3, 126.1, 114.9, 114.6, 91.1, 55.5, 55.4, 42.9, 42.6, 37.1, 29.9, 28.5, 27.8. HPLC (Lux 3µm i-Cellulose 5, 100 X 4.6 mm, Hexane/i-Propanol 80:20, flow: 0.5 mL/min, λ=210 nm), t_{minor}: 25.4 min; t_{major}: min. $[\alpha]_D^{25}$ = +692.3 (c = $1.45 \cdot 10^{-3}$ g/mL, $CHCl_3$). ESI-MS: $C_{21}H_{18}F_3NO_4Na$ [M+Na]+ calcd.: 428.1086, found: 428.1086.

(1'R,2'R,3'S)-4''-Methoxy-2'-nitro-4-(trifluoromethyl)-1',2',3',6'-tetrahydro-[1,1':3',1''-terphenyl]-4'-carbaldehyde **5j''**

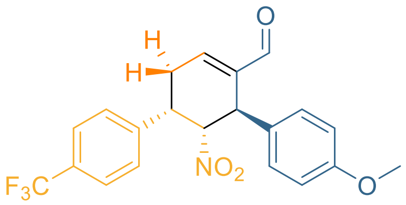

The desired product was obtained, using (*R*)-(**1**), as an oil (37.0 mg, 0.0913 mmol, yield = 18.3%, ee= 96%, (*R*)-(**1**)). ^{1}H NMR (400 MHz, $CDCl_3$, 303 K) δ 9.59 (s, 1H), 7.64 (d, J = 8.1 Hz, 1H), 7.56 (d, J = 8.2 Hz, 1H), 7.43 – 7.34 (m, 2H), 7.17 (m, 1H), 6.97 (d, J = 8.8 Hz, 1H), 6.91 (d, J = 8.8 Hz, 1H), 6.82 (d, J = 8.8 Hz, 2H), 4.95 – 4.86 (m, 1H), 4.54 (s, 1H), 3.79 (two s, rotamers, 3H), 3.37 – 3.17 (m, 2H), 2.92 (m, 1H).. ^{13}C NMR (101 MHz, $CDCl_3$, 303 K) δ 191.4, 163.8, 161.4, 150.5, 149.6, 141.9, 138.3, 137.9, 134.5, 130.8, 129.8, 129.7, 129.2, 129.1, 128.6, 127.9, 126.5, 126.4, 126.1, 126.1, 126.1, 116.6, 116.4, 116.3, 116.1, 90.9, 90.8, 42.9, 42.5, 37.3, 37.1, 29.9, 28.3, 27.8. HPLC (Lux 3µm i-Cellulose 5, 100 X 4.6 mm, Hexane/i-Propanol 80:20, flow: 0.5 mL/min, λ=210 nm), t_{minor}: 18.1 min; t_{major}: 14.5 min. $[\alpha]_D^{25}$

= +750.34 (c = 1.3·10^{-3} g/mL, $CHCl_3$). ESI-MS: $C_{21}H_{18}F_3NO_4Na$ [M+Na]+ calcd.: 428.1086, found: 428.1075.

3. NMR spectra

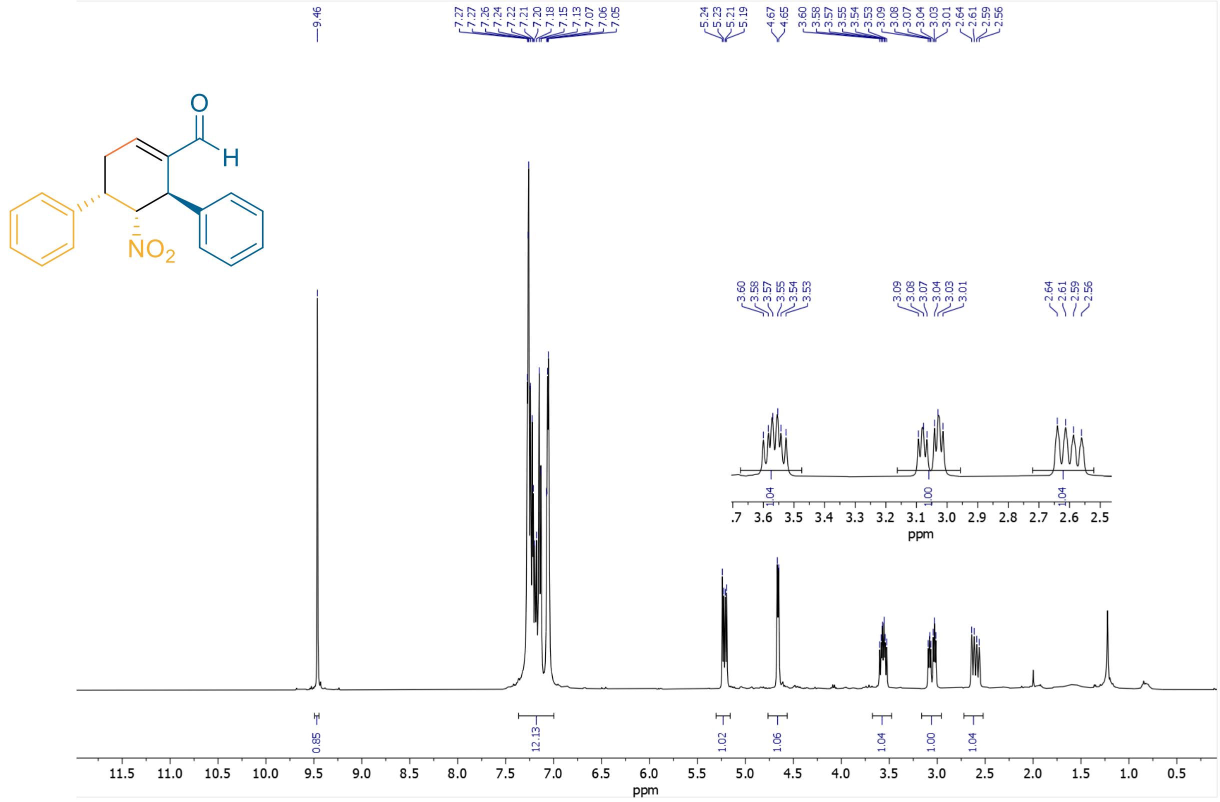

Figure S1: ^{1}H-NMR (400 MHz) spectrum of 5a' in $CDCl_3$.

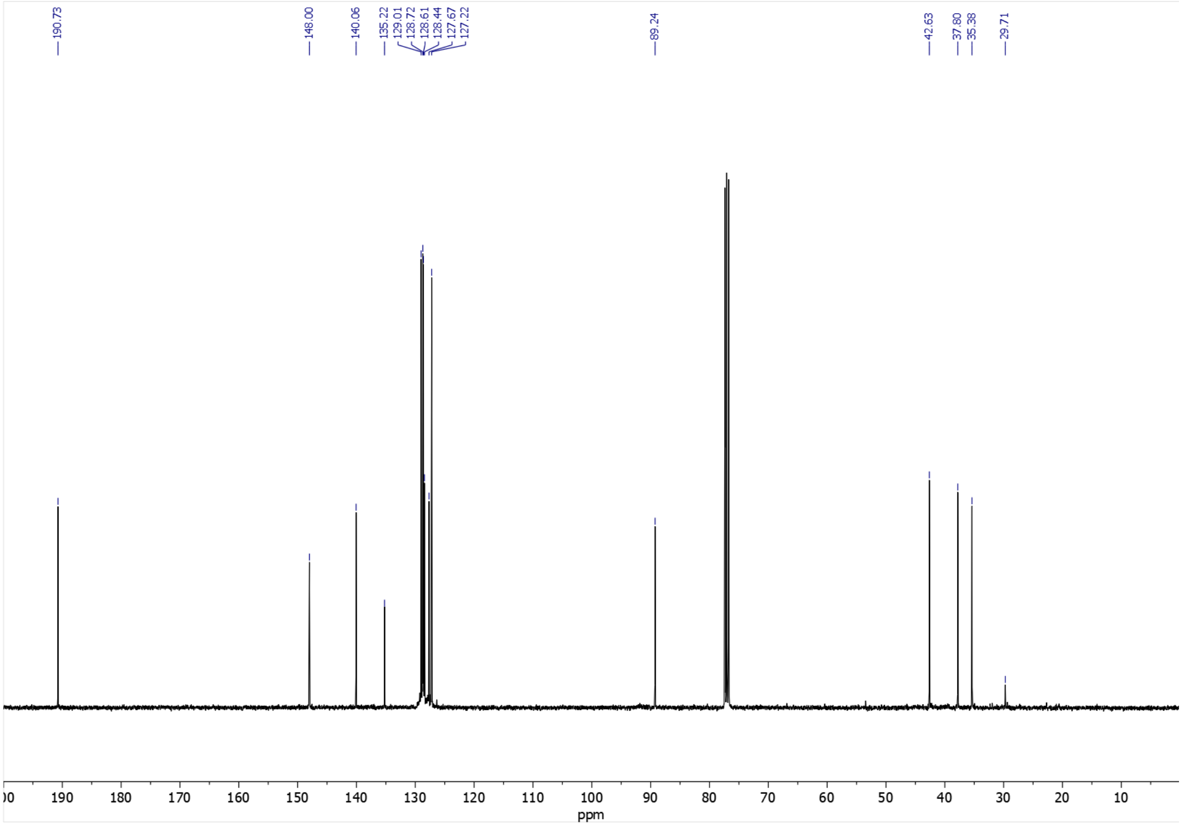

Figure S2: ^{13}C-NMR (101 MHz) spectrum of 5a' in $CDCl_3$.

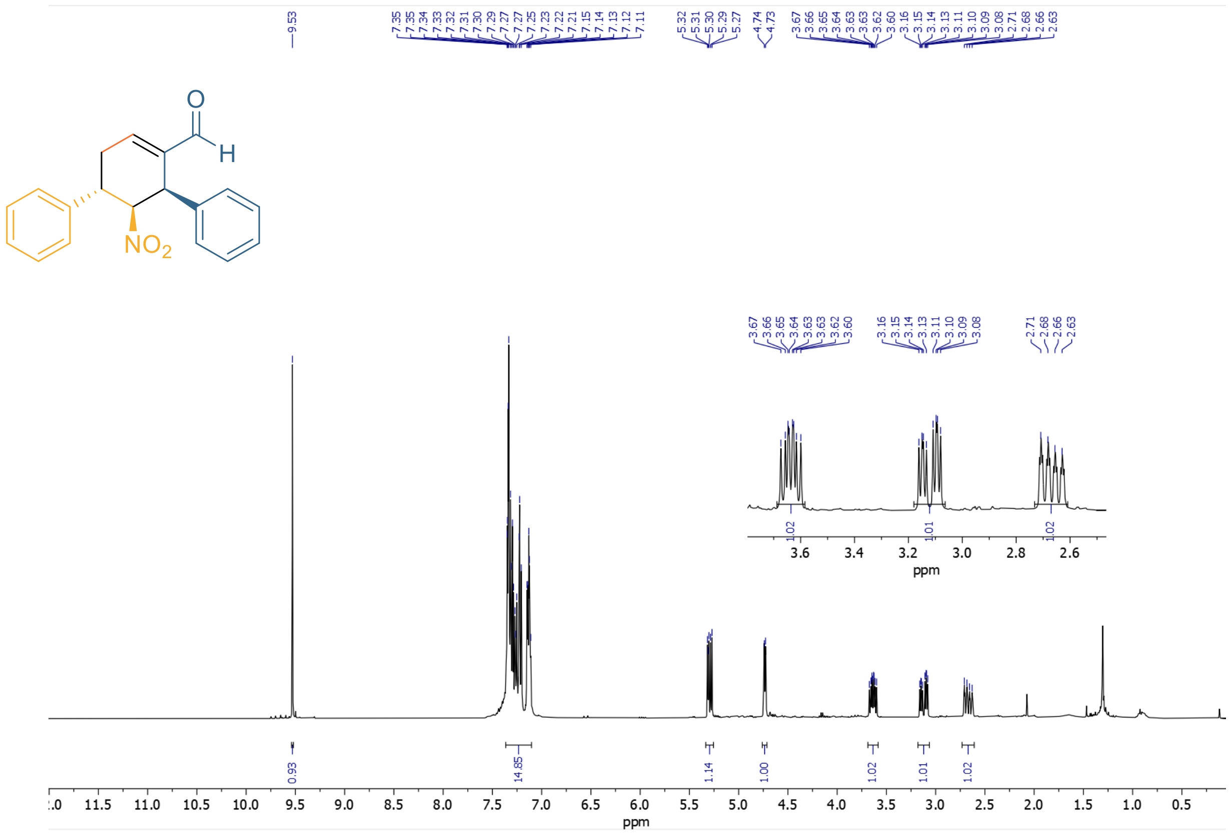

Figure S3: ^{1}H-NMR (400 MHz) spectrum of 5a'' in $CDCl_3$.

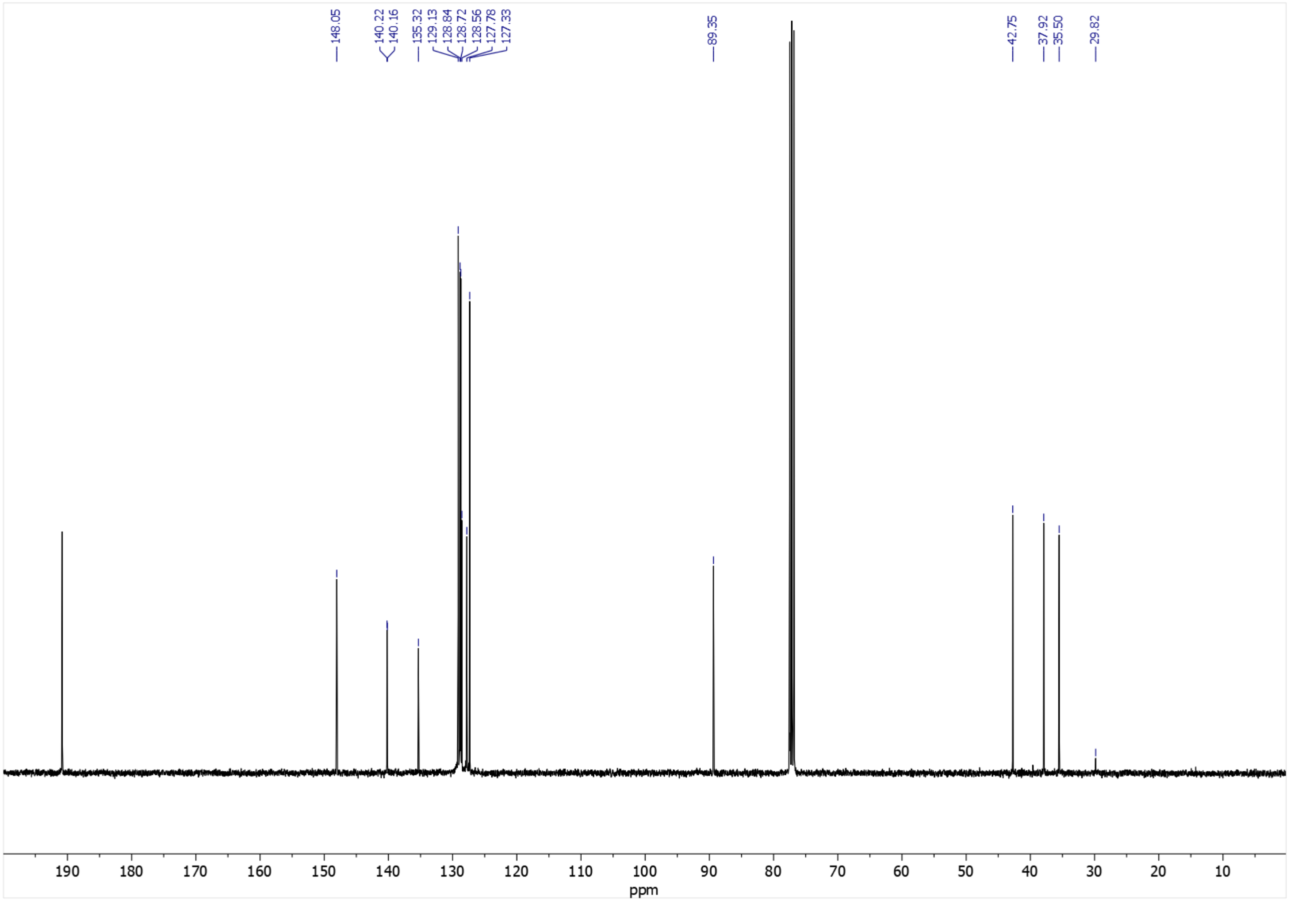

Figure S4: ^{13}C-NMR (101 MHz) spectrum of 5a'' in $CDCl_3$.

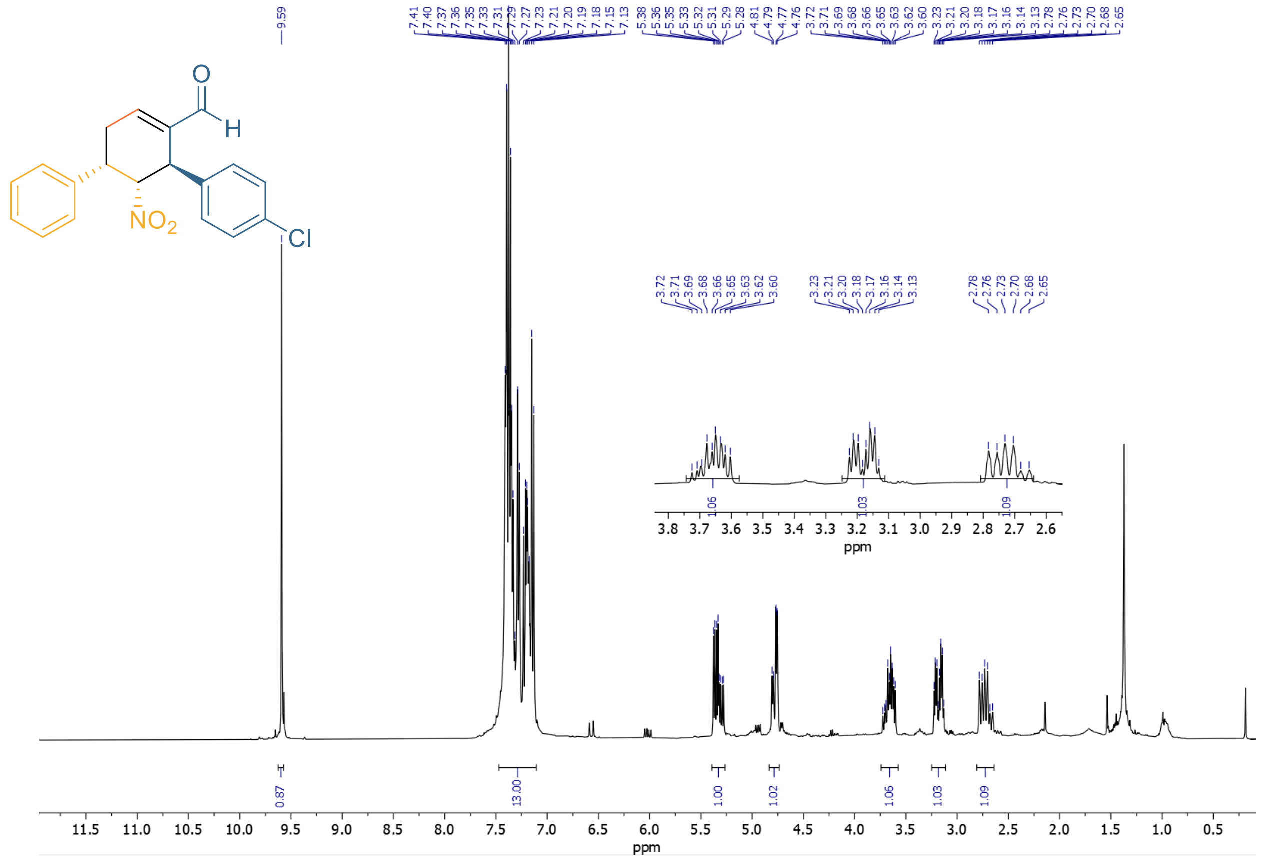

Figure S5: ^{1}H-NMR (400 MHz) spectrum of 5b' in $CDCl_3$.

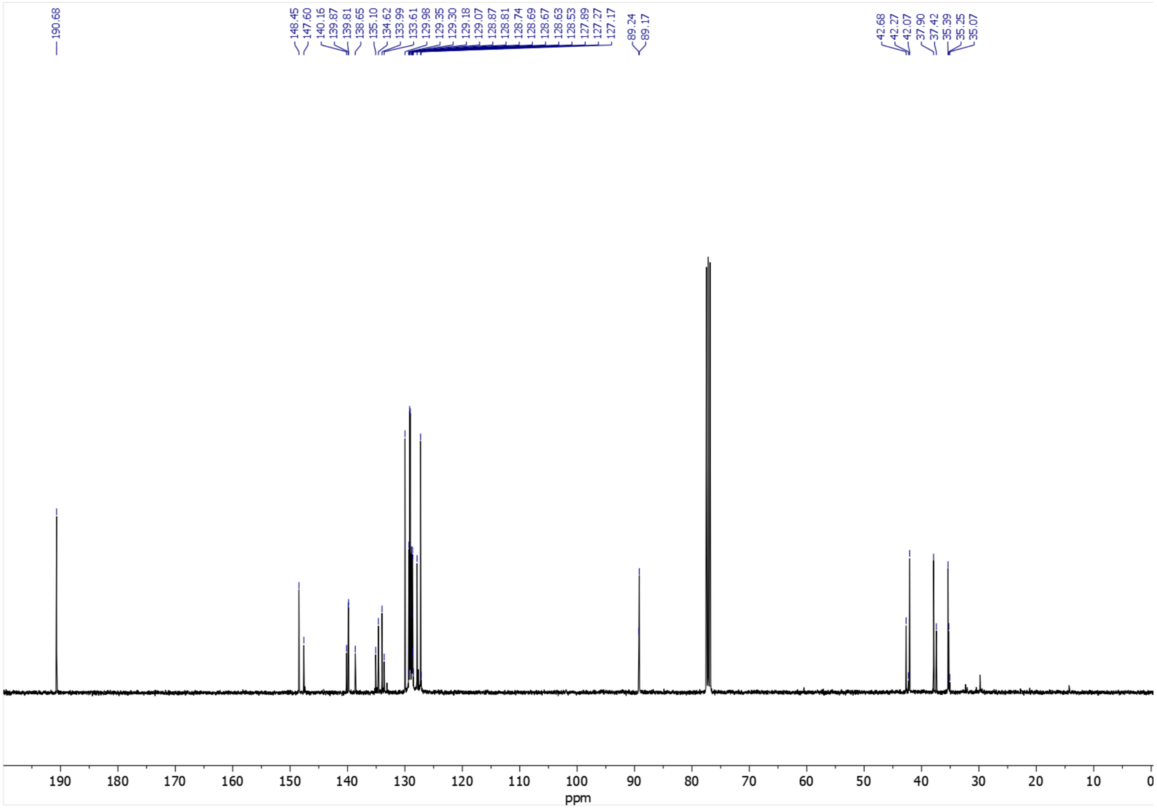

Figure S6: ^{13}C-NMR (101 MHz) spectrum of 5b' in $CDCl_3$.

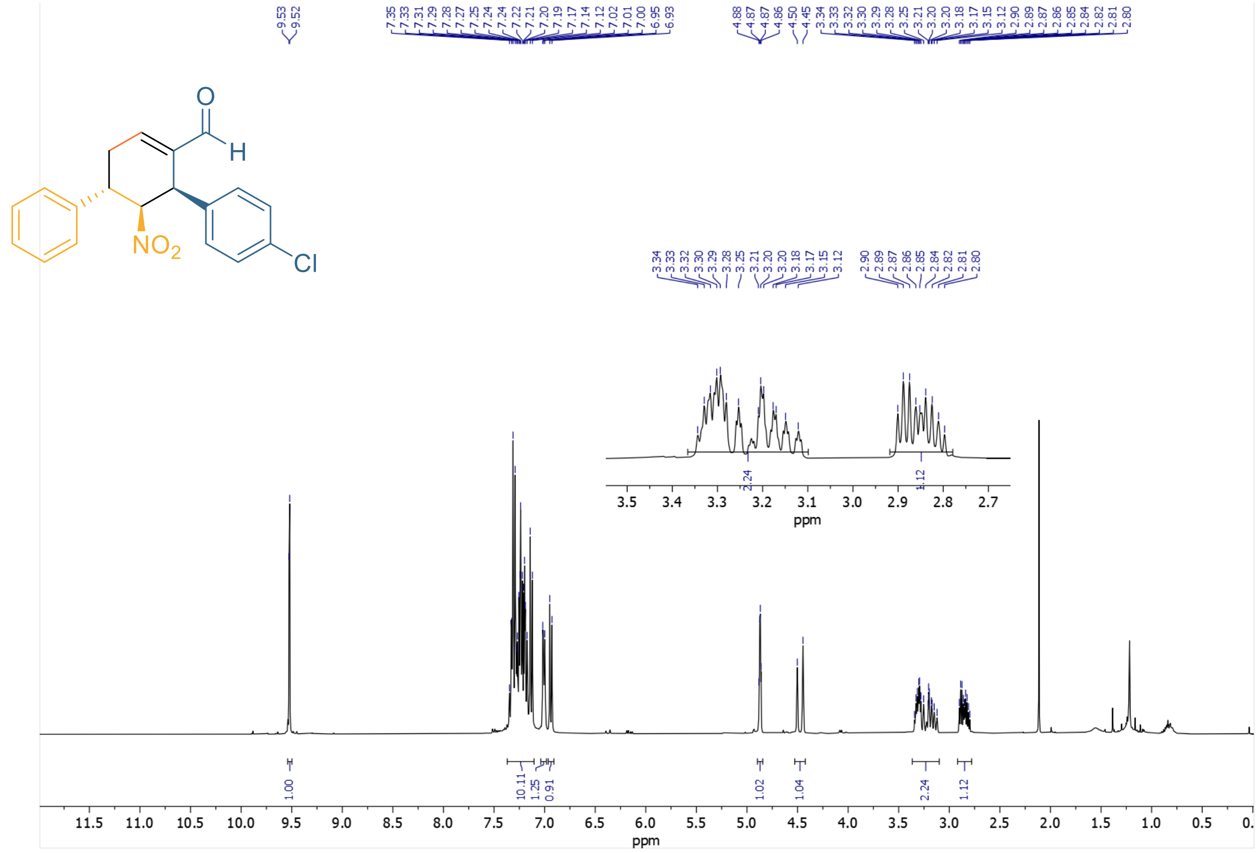

Figure S7: ^{1}H-NMR (400 MHz) spectrum of 5b'' in $CDCl_3$.

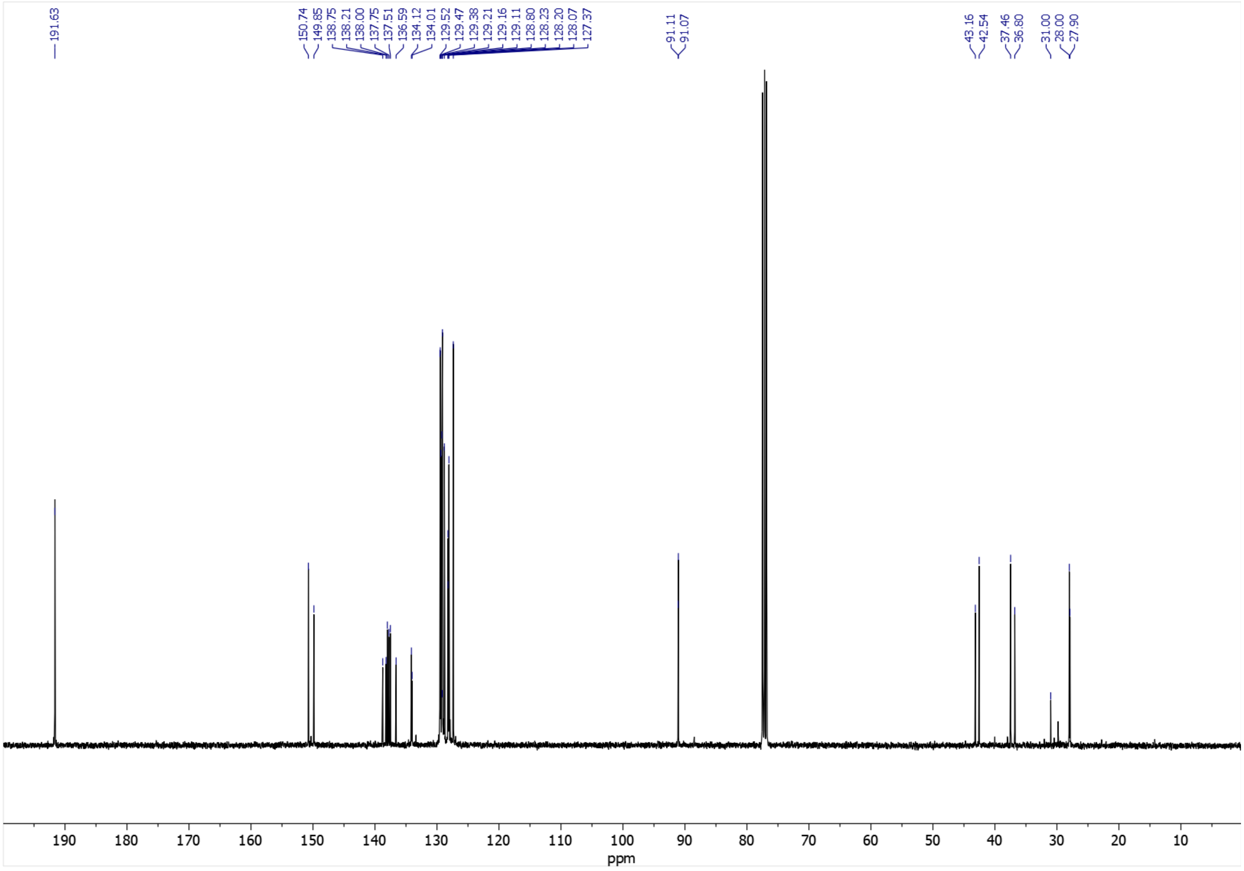

Figure S8: ^{13}C-NMR (101 MHz) spectrum of 5b'' in $CDCl_3$.

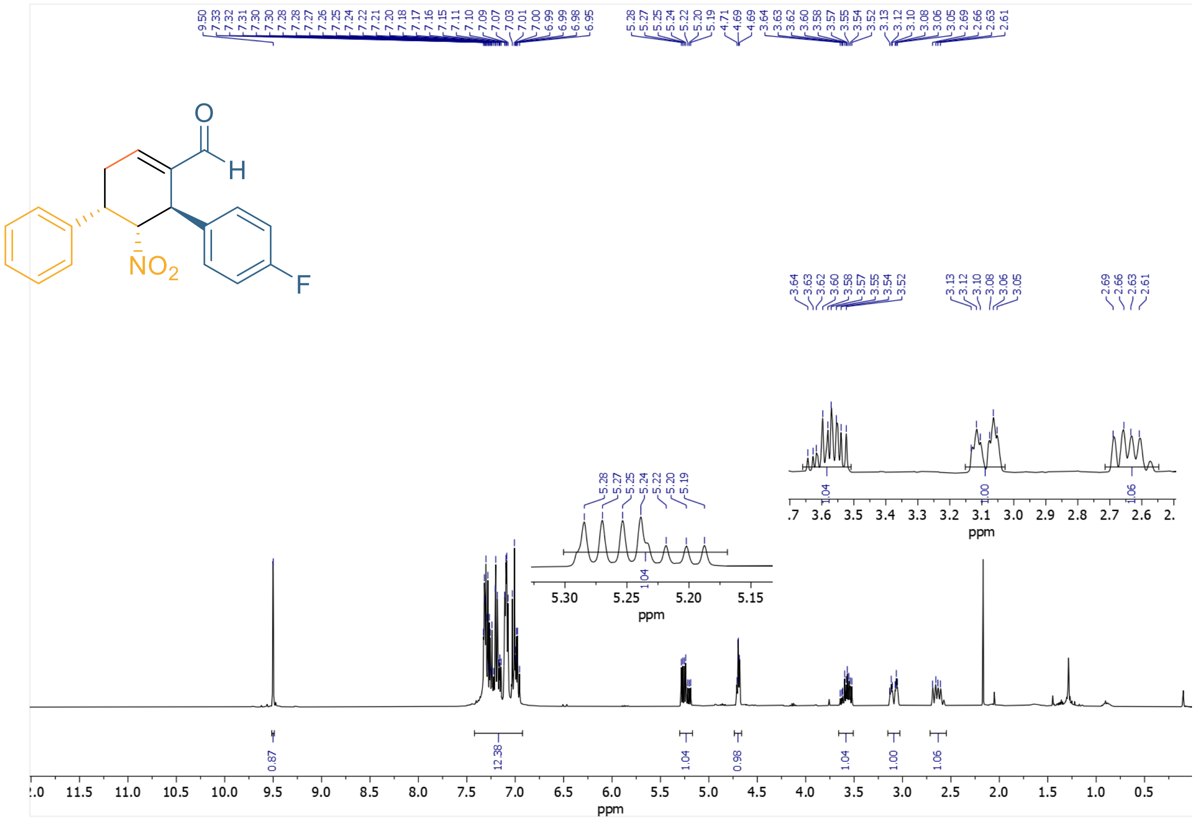

Figure S9: ^{1}H-NMR (400 MHz) spectrum of 5c' in $CDCl_3$.

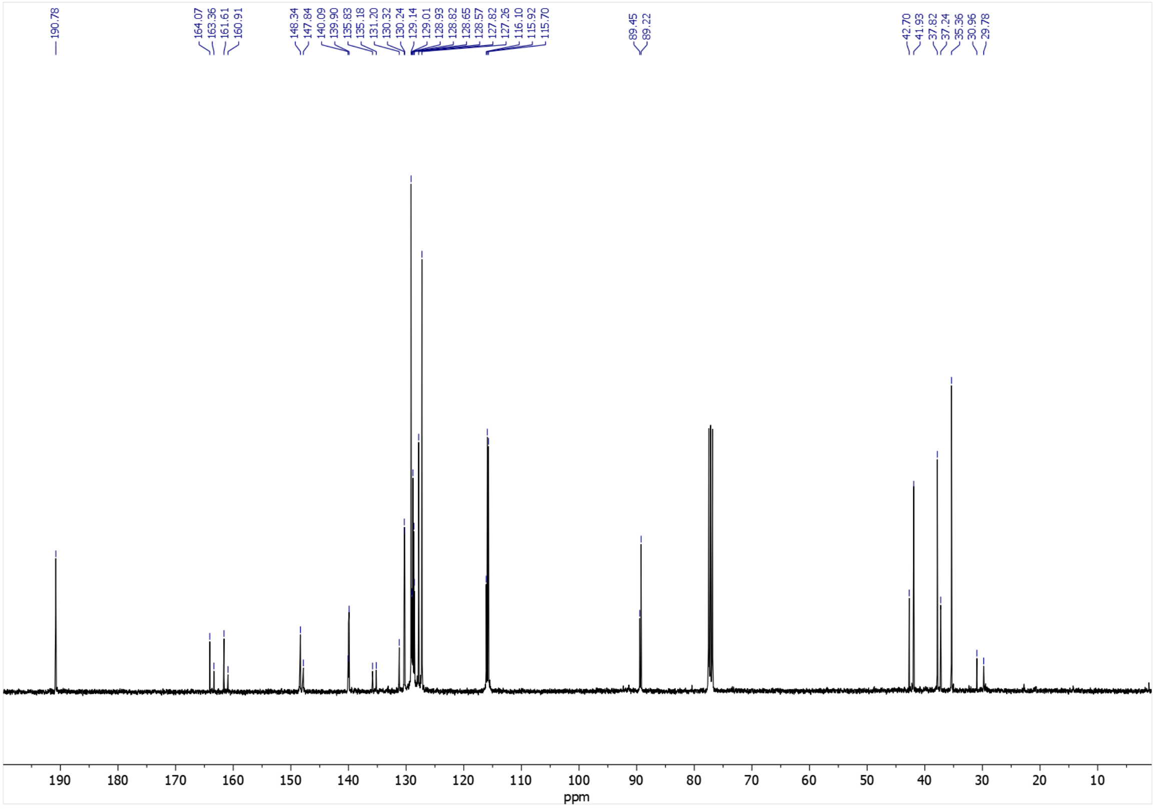

Figure S10: ^{13}C-NMR (101 MHz) spectrum of 5c' in $CDCl_3$.

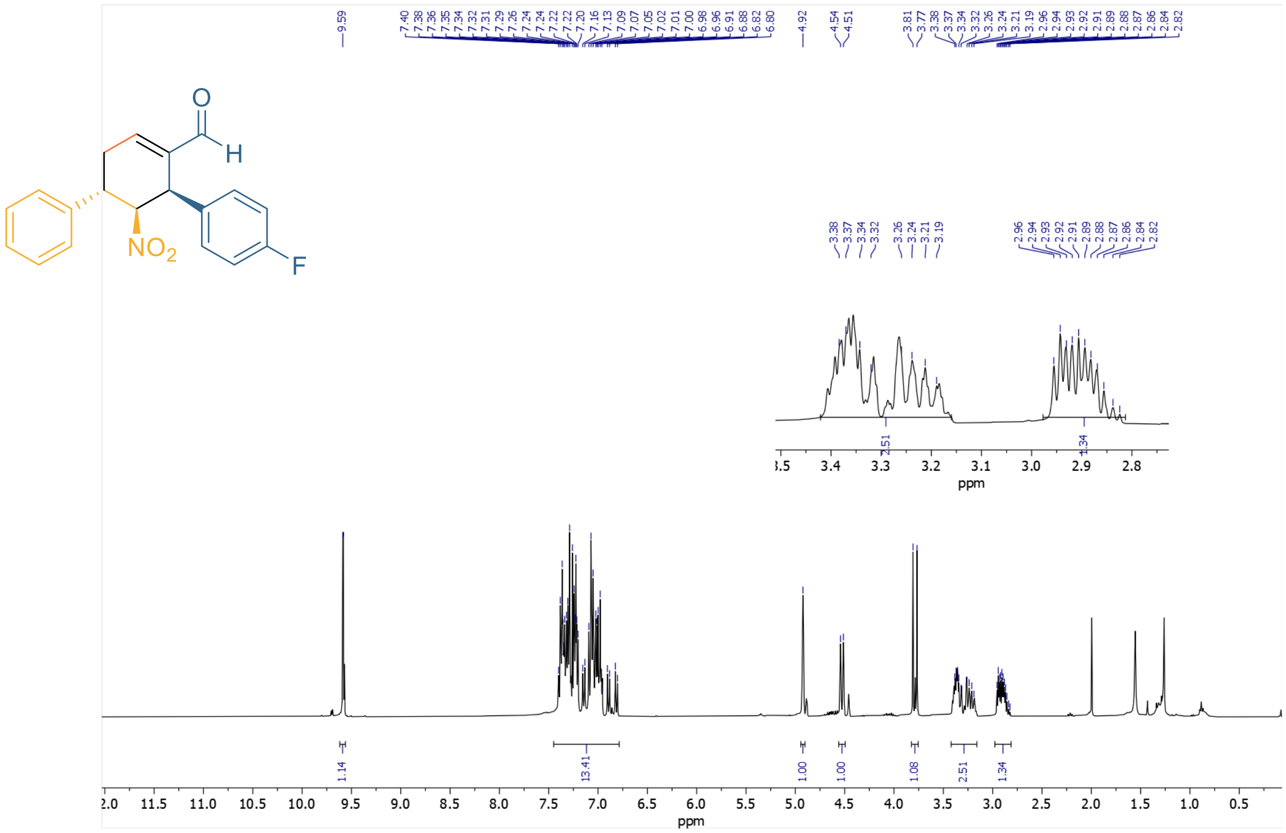

Figure S11: ^{1}H-NMR (400 MHz) spectrum of 5c'' in $CDCl_3$.

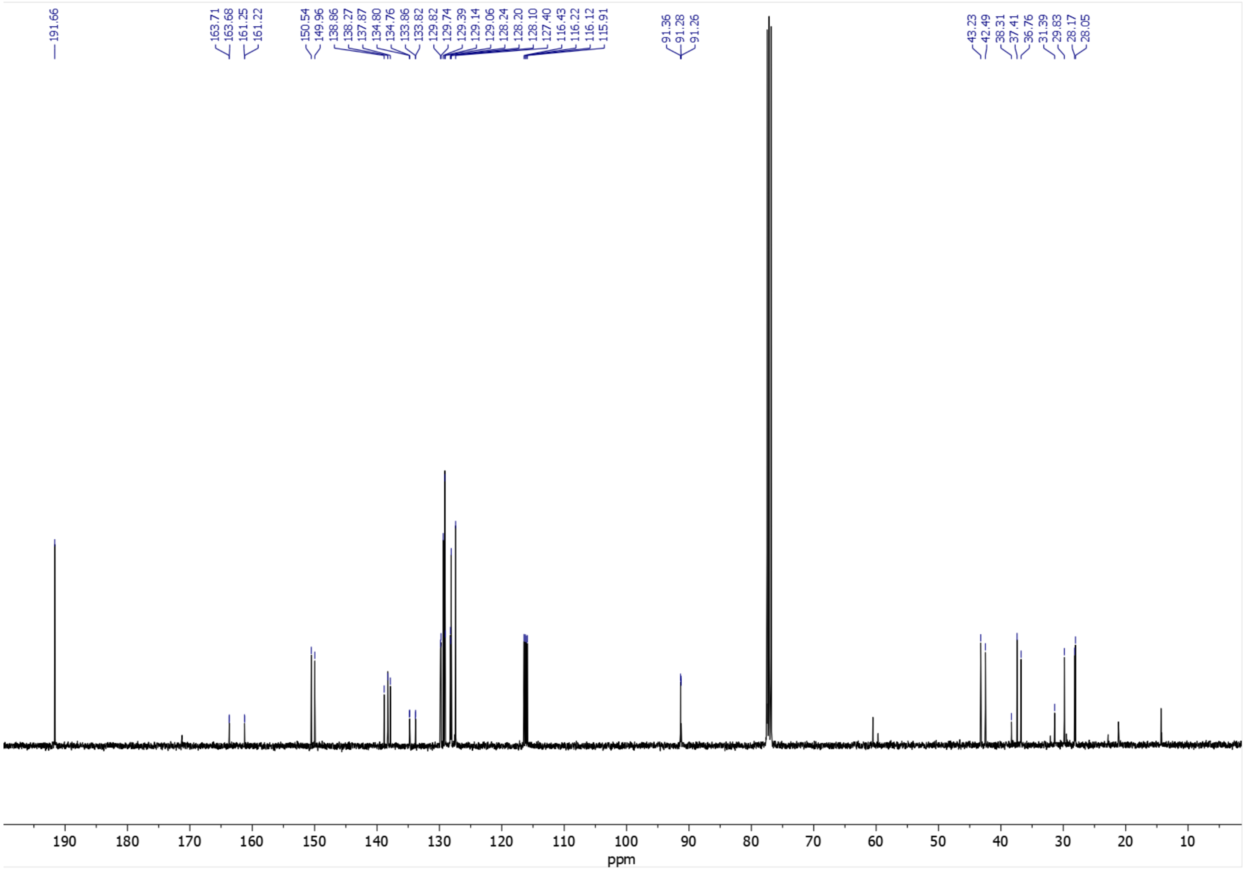

Figure S12: ^{13}C-NMR (101 MHz) spectrum of 5c'' in $CDCl_3$.

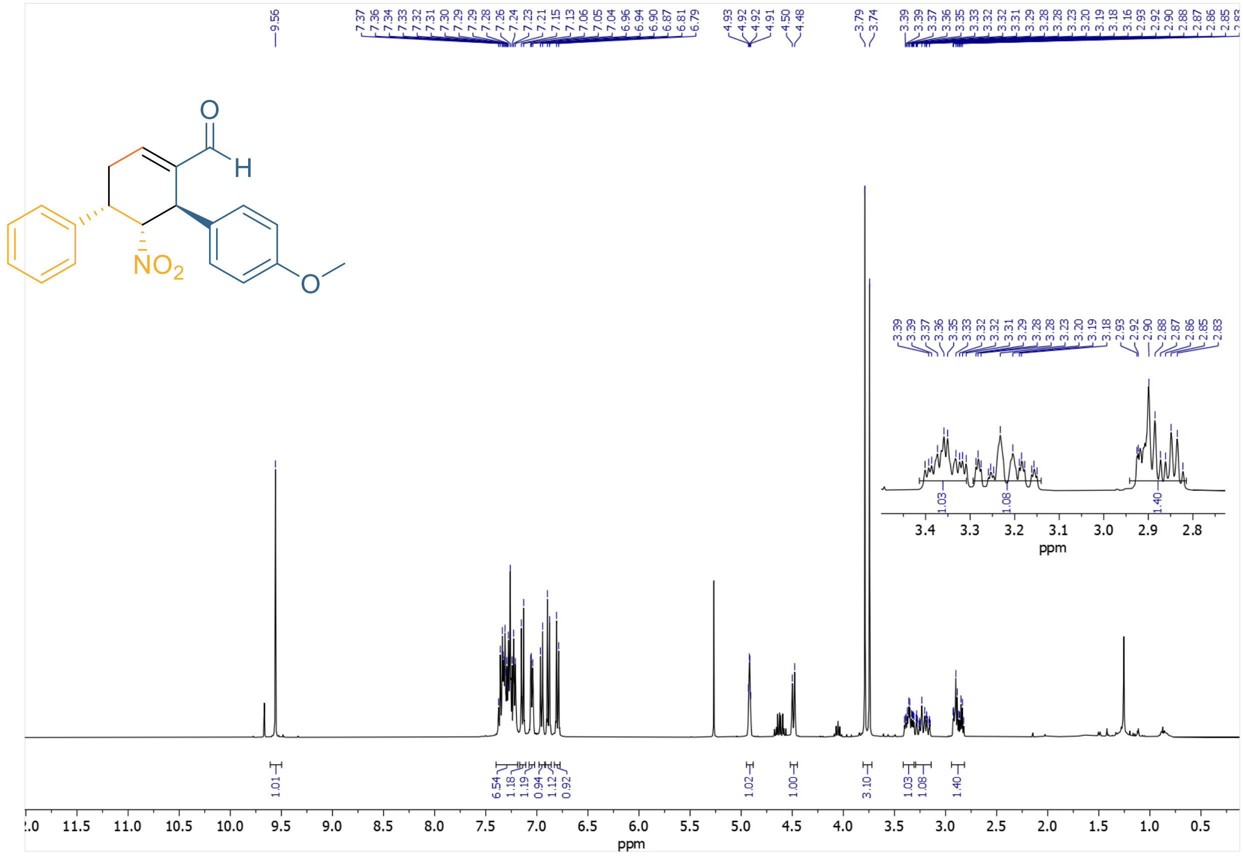

Figure S13: ^{1}H-NMR (400 MHz) spectrum of 5d' in $CDCl_3$.

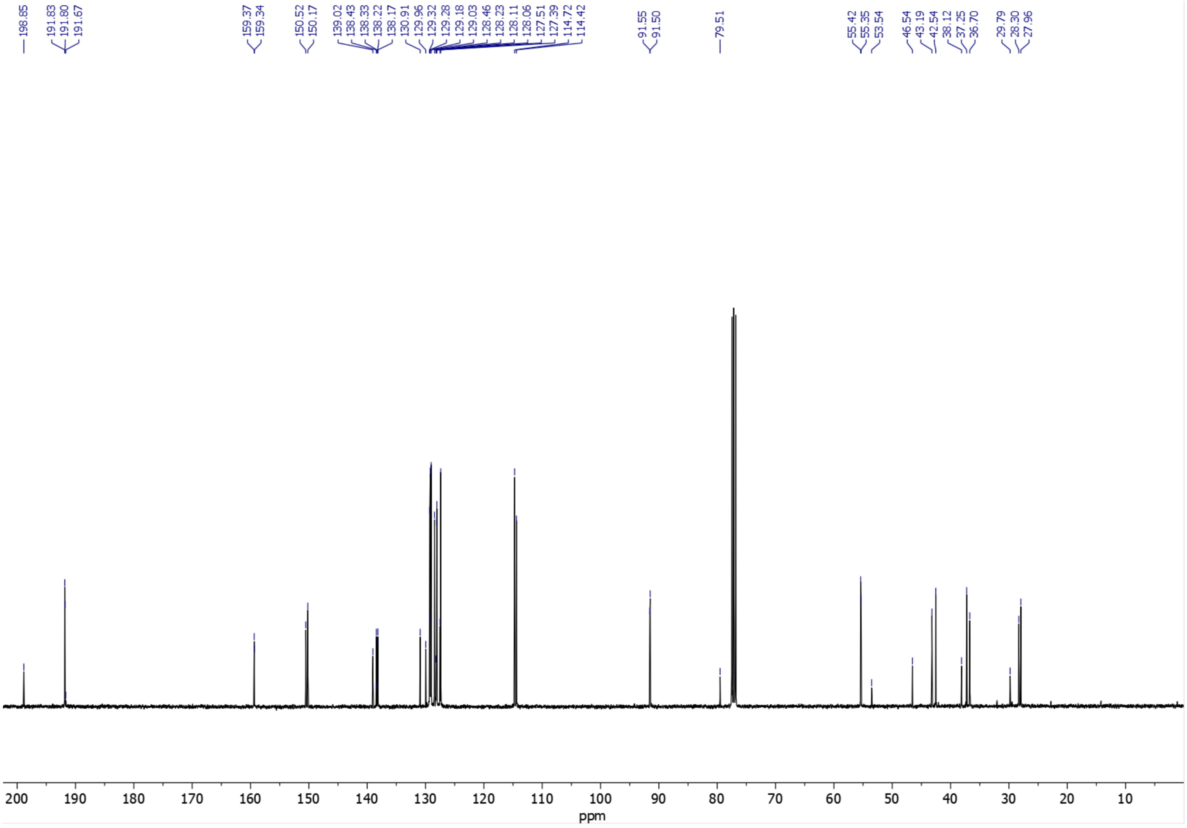

Figure S14: ^{13}C-NMR (101 MHz) spectrum of 5d' in $CDCl_3$.

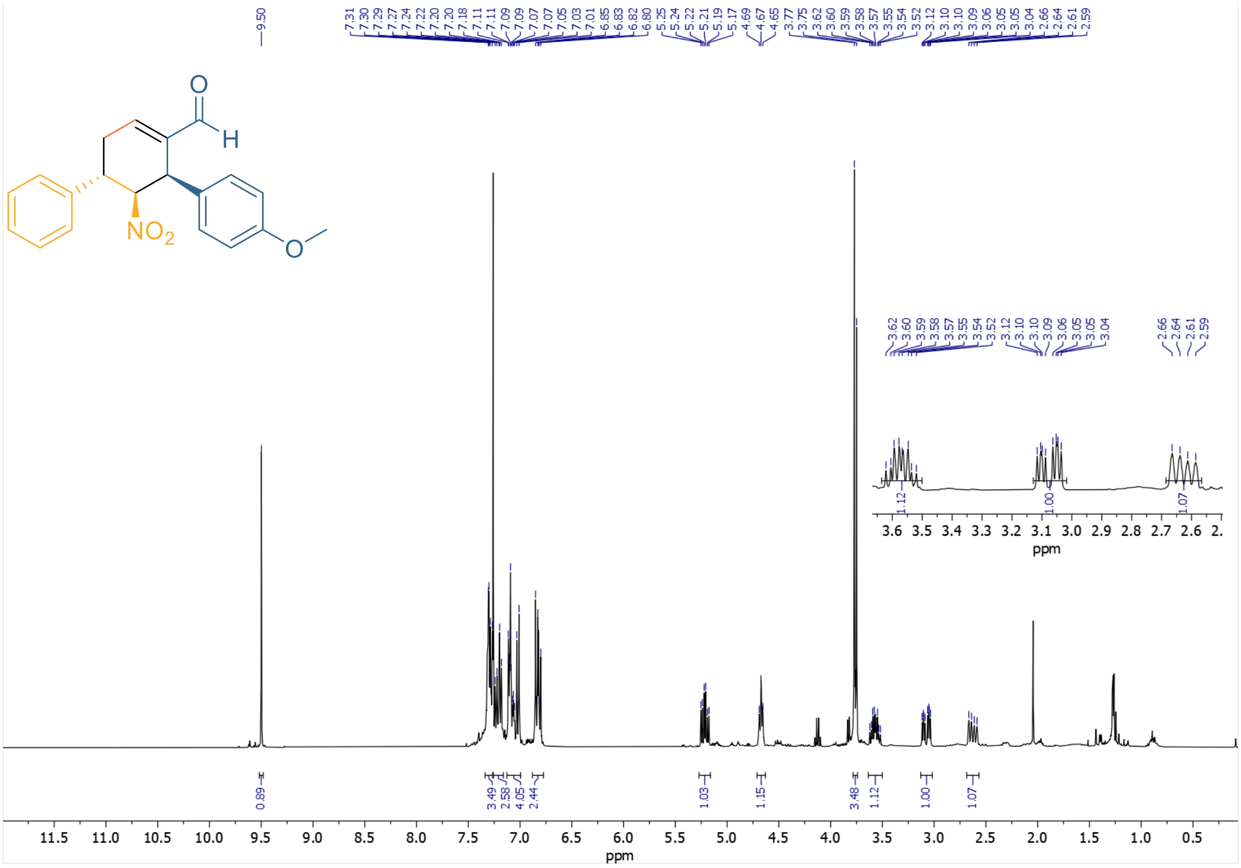

Figure S15: ^{1}H-NMR (400 MHz) spectrum of 5d'' in $CDCl_3$.

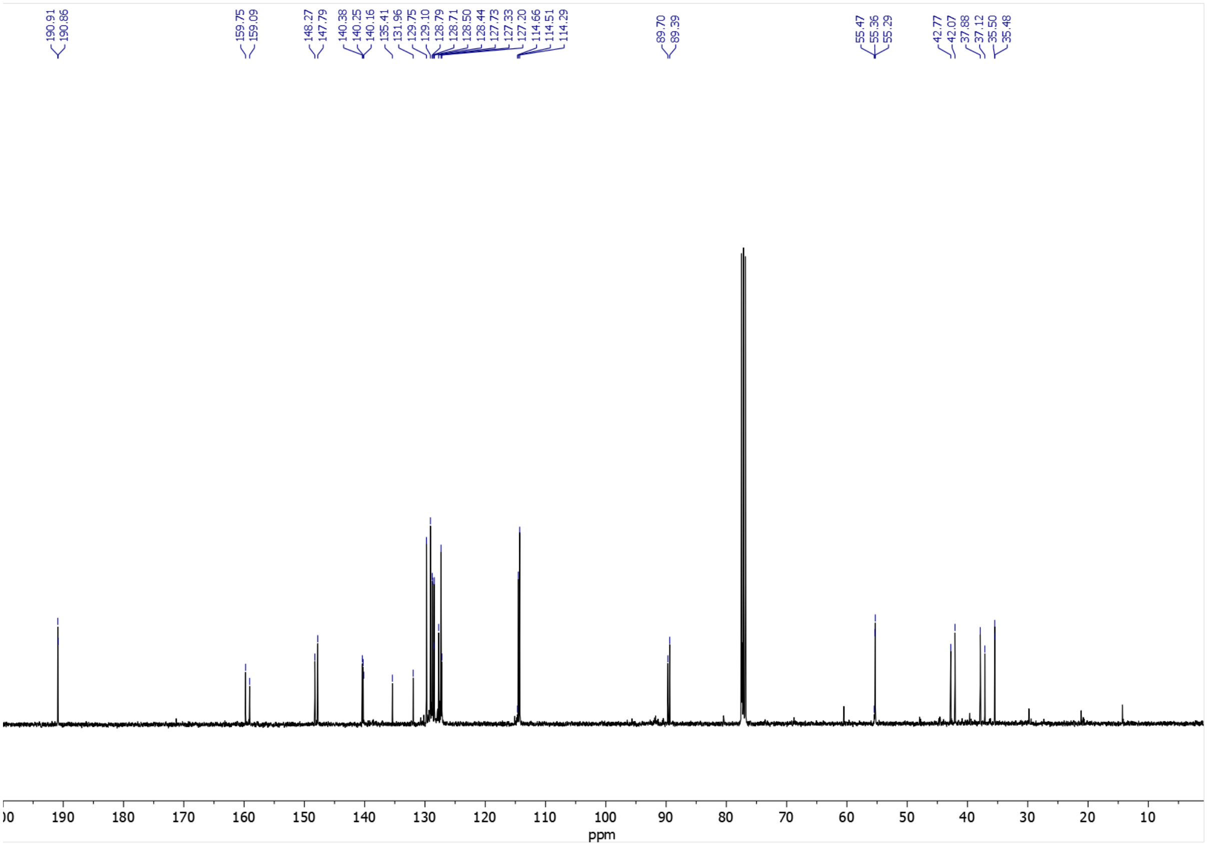

Figure S16: ^{13}C-NMR (101 MHz) spectrum of 5d'' in $CDCl_3$.

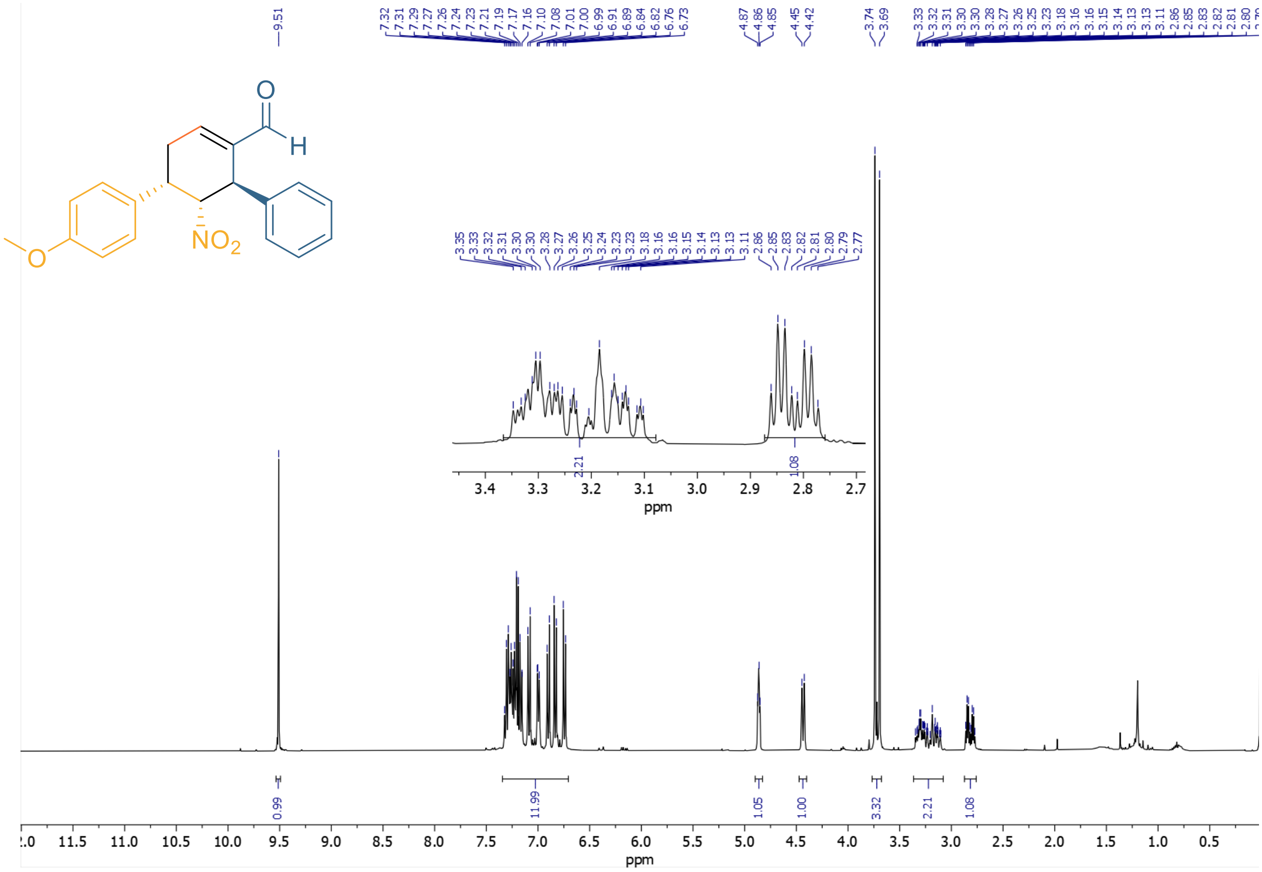

Figure S17: ^{1}H-NMR (400 MHz) spectrum of 5e' in $CDCl_3$.

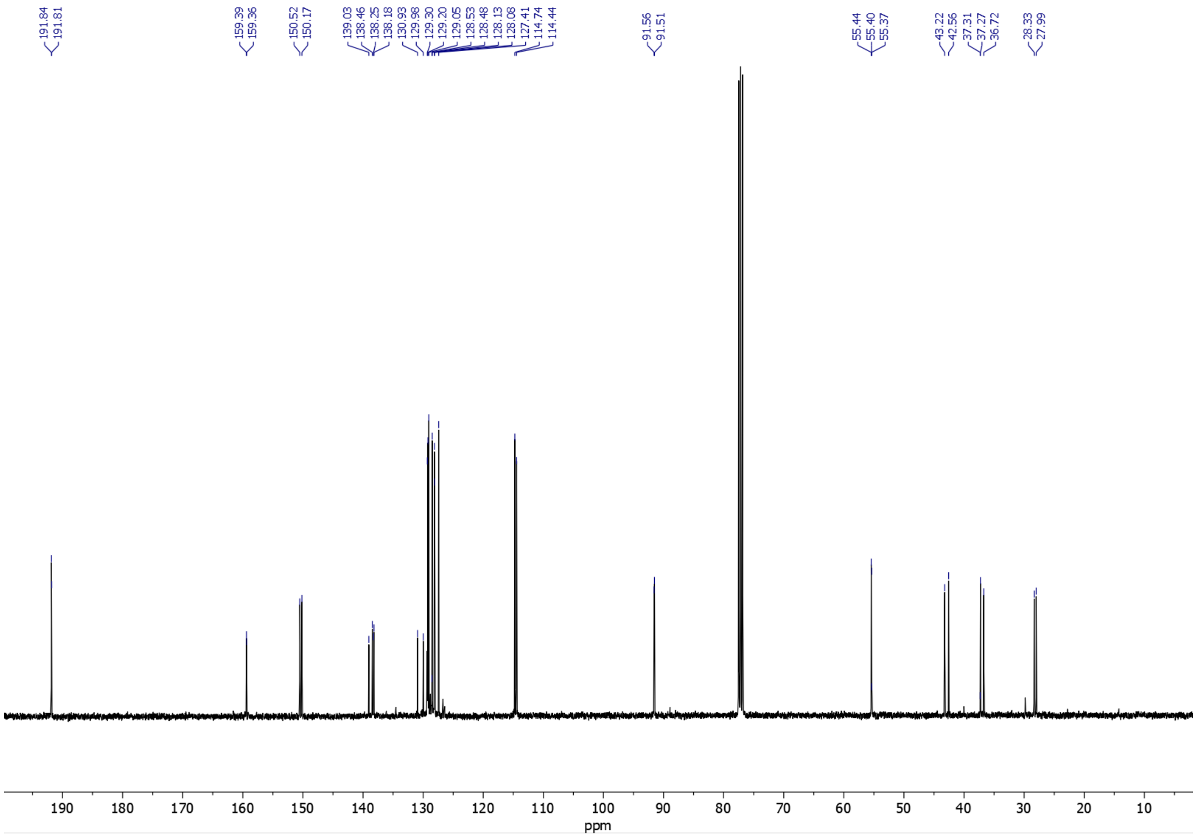

Figure S18: ^{13}C-NMR (101 MHz) spectrum of 5e' in $CDCl_3$.

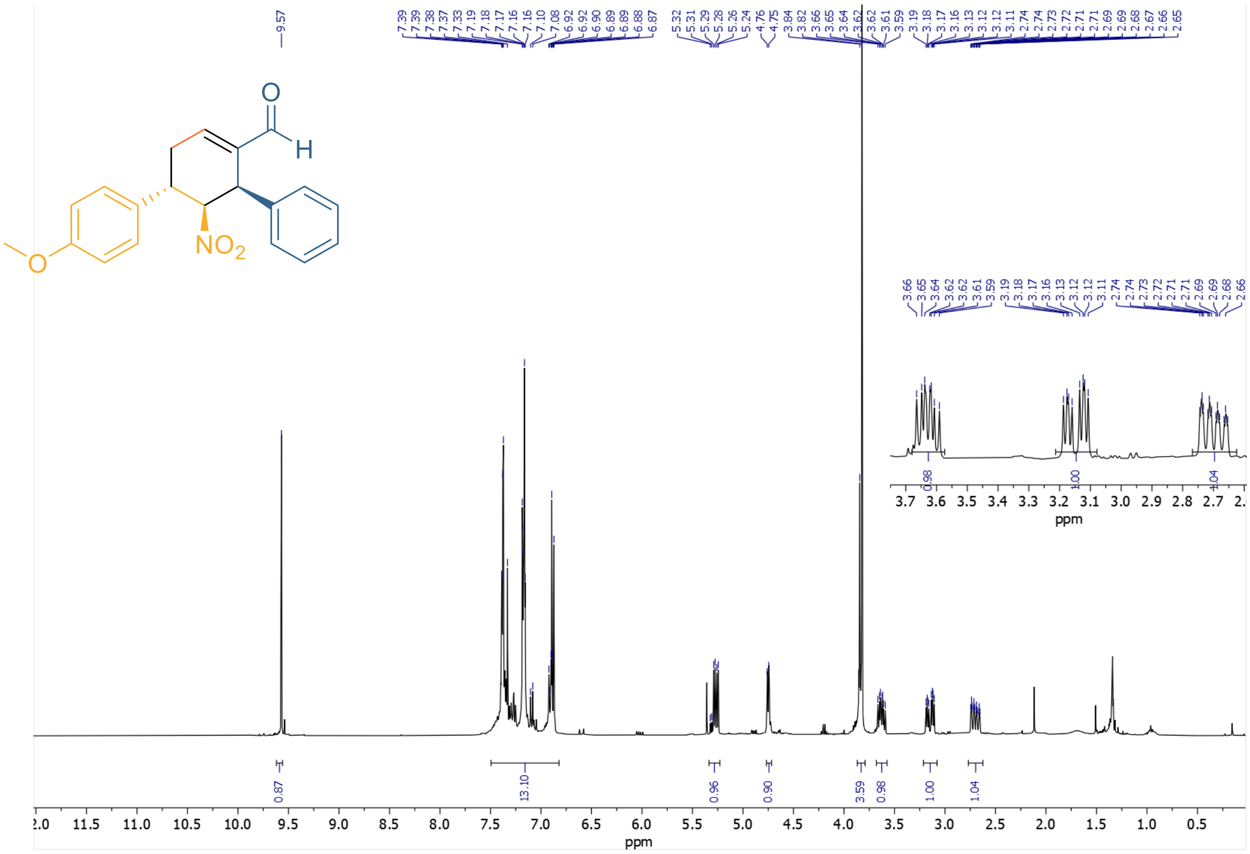

Figure S19: ^{1}H-NMR (400 MHz) spectrum of 5e'' in $CDCl_3$.

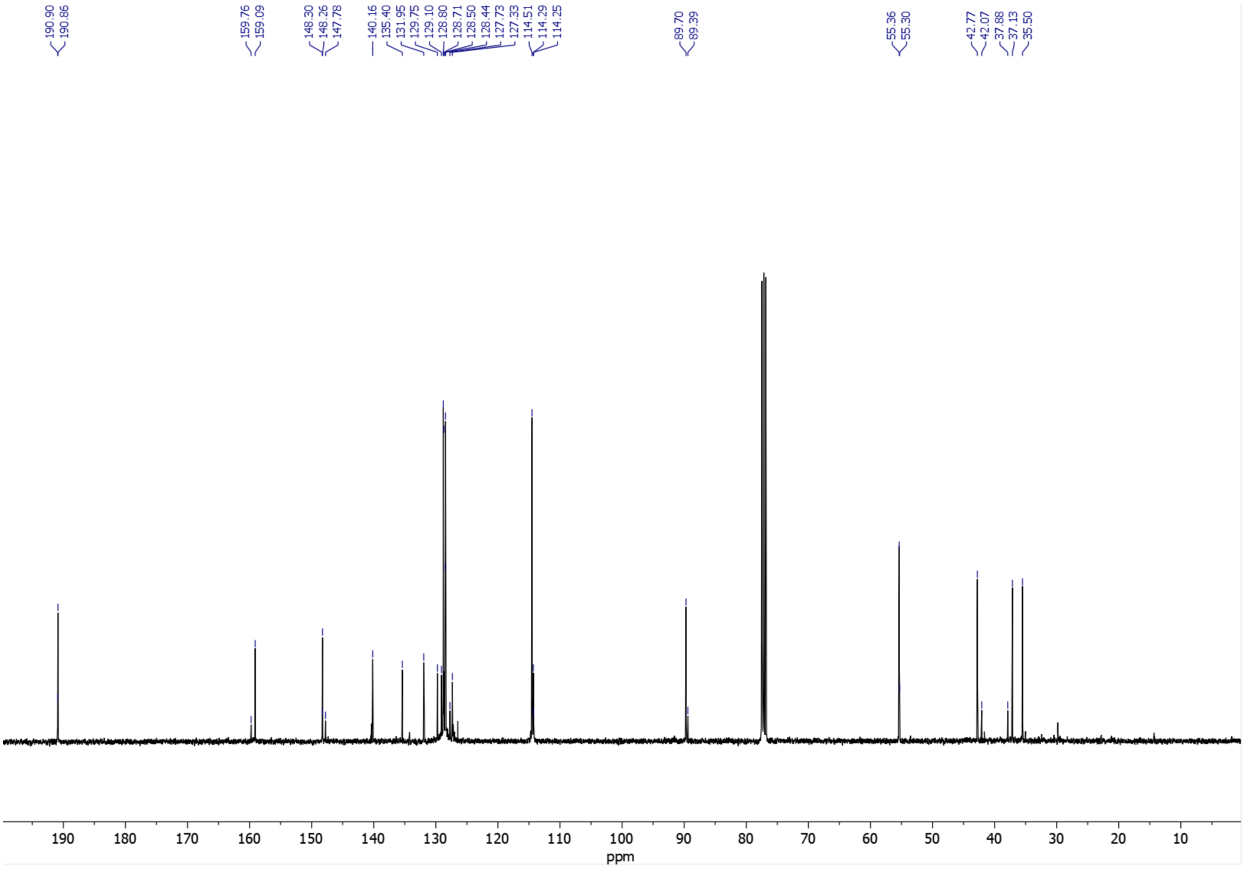

Figure S20: ^{13}C-NMR (101 MHz) spectrum of 5e'' in $CDCl_3$.

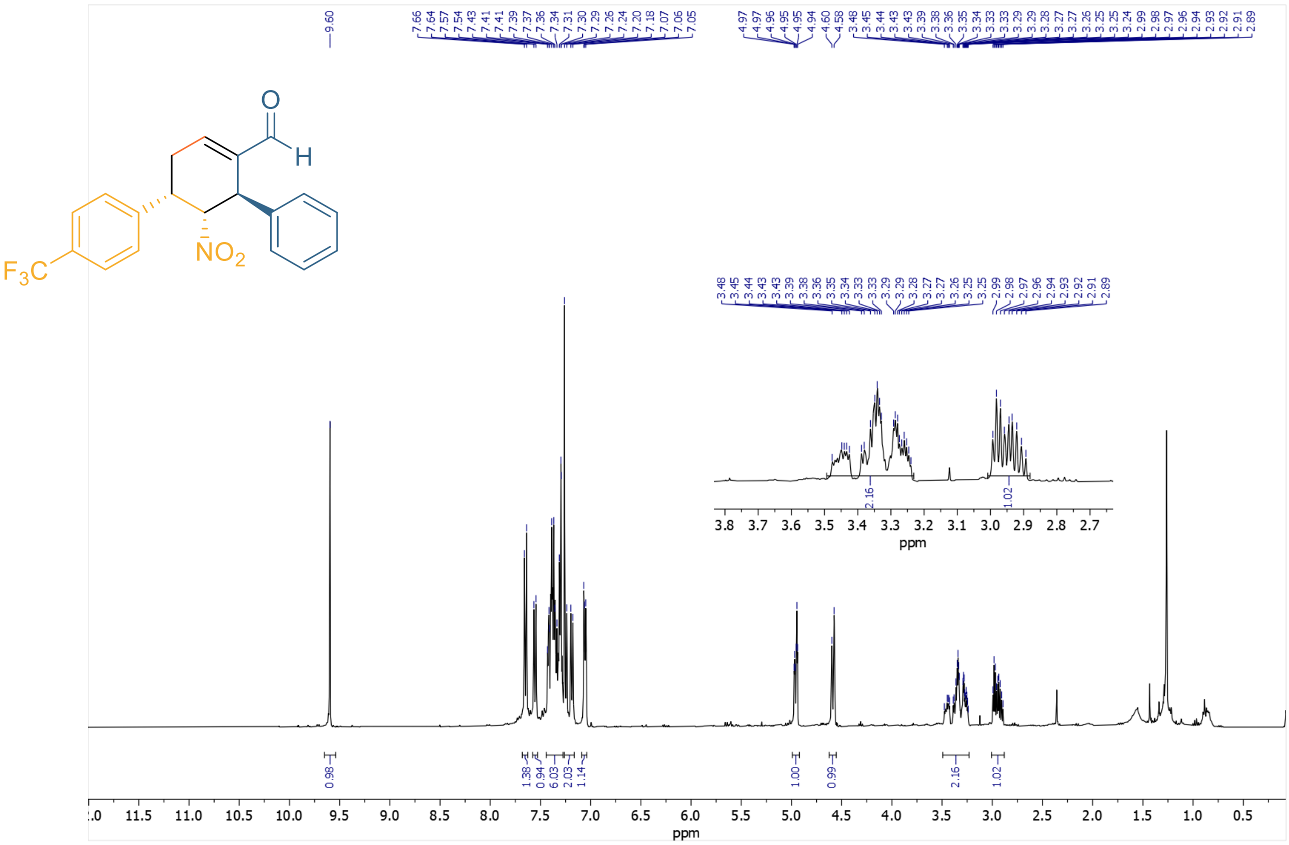

Figure S21: ^{1}H-NMR (400 MHz) spectrum of 5f' in CDCl₃.

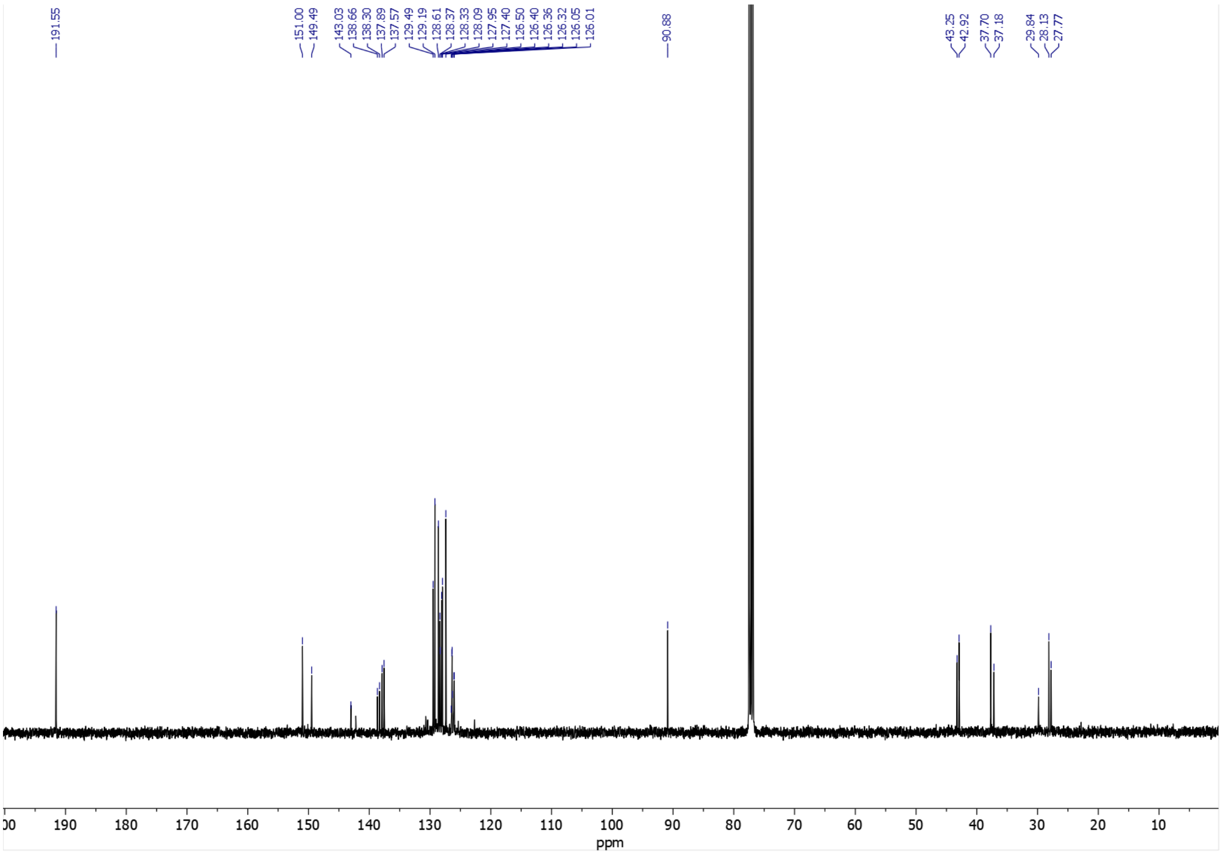

Figure S22: ^{13}C-NMR (101 MHz) spectrum of 5f' in CDCl₃.

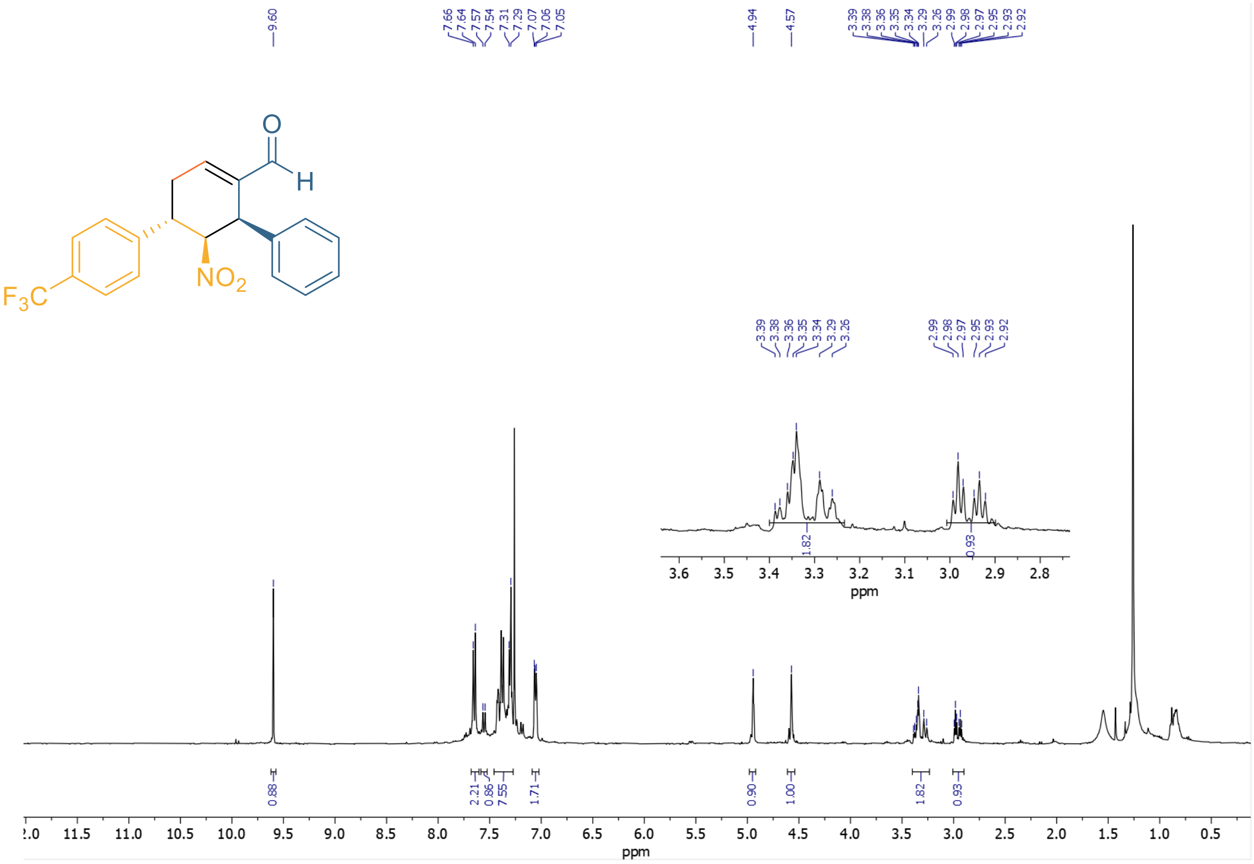

Figure S23: ^{1}H-NMR (400 MHz) spectrum of 5f'' in $CDCl_3$.

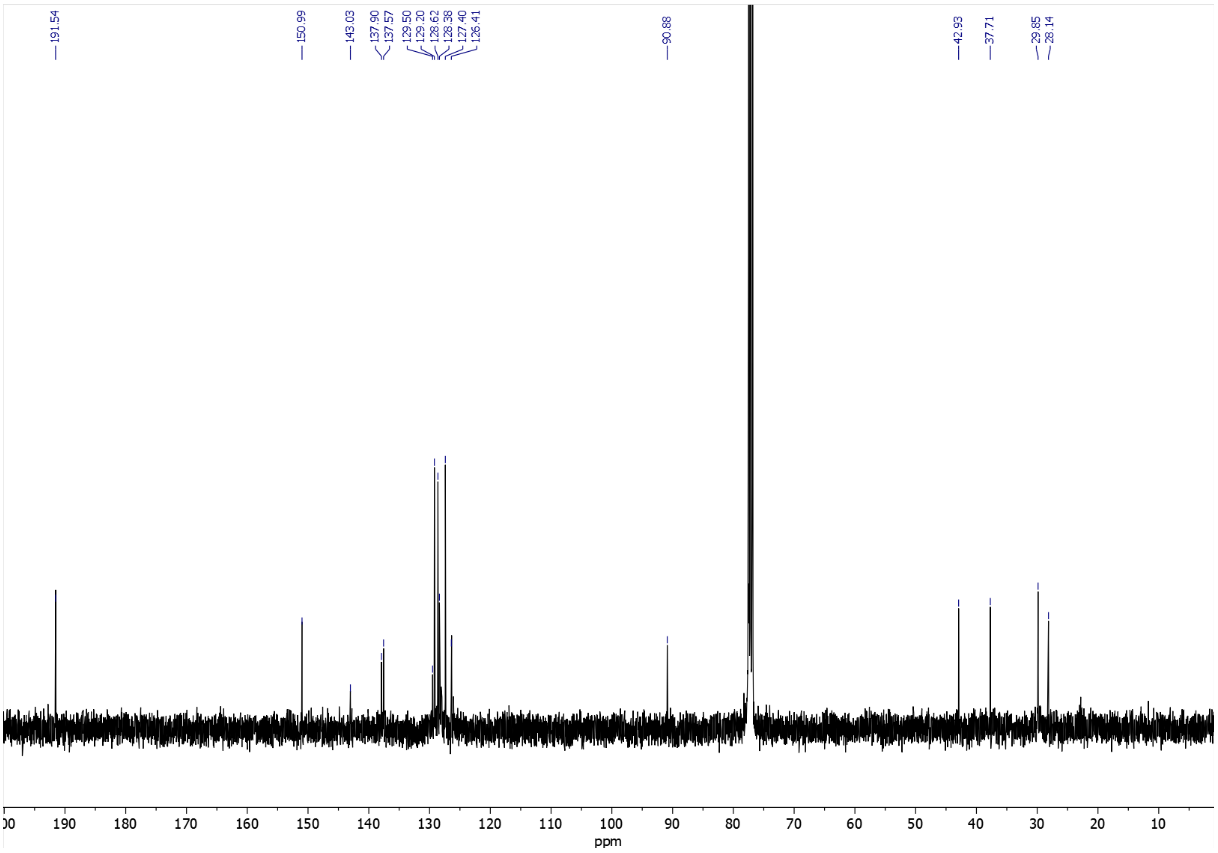

Figure S24: ^{13}C-NMR (101 MHz) spectrum of 5f'' in $CDCl_3$.

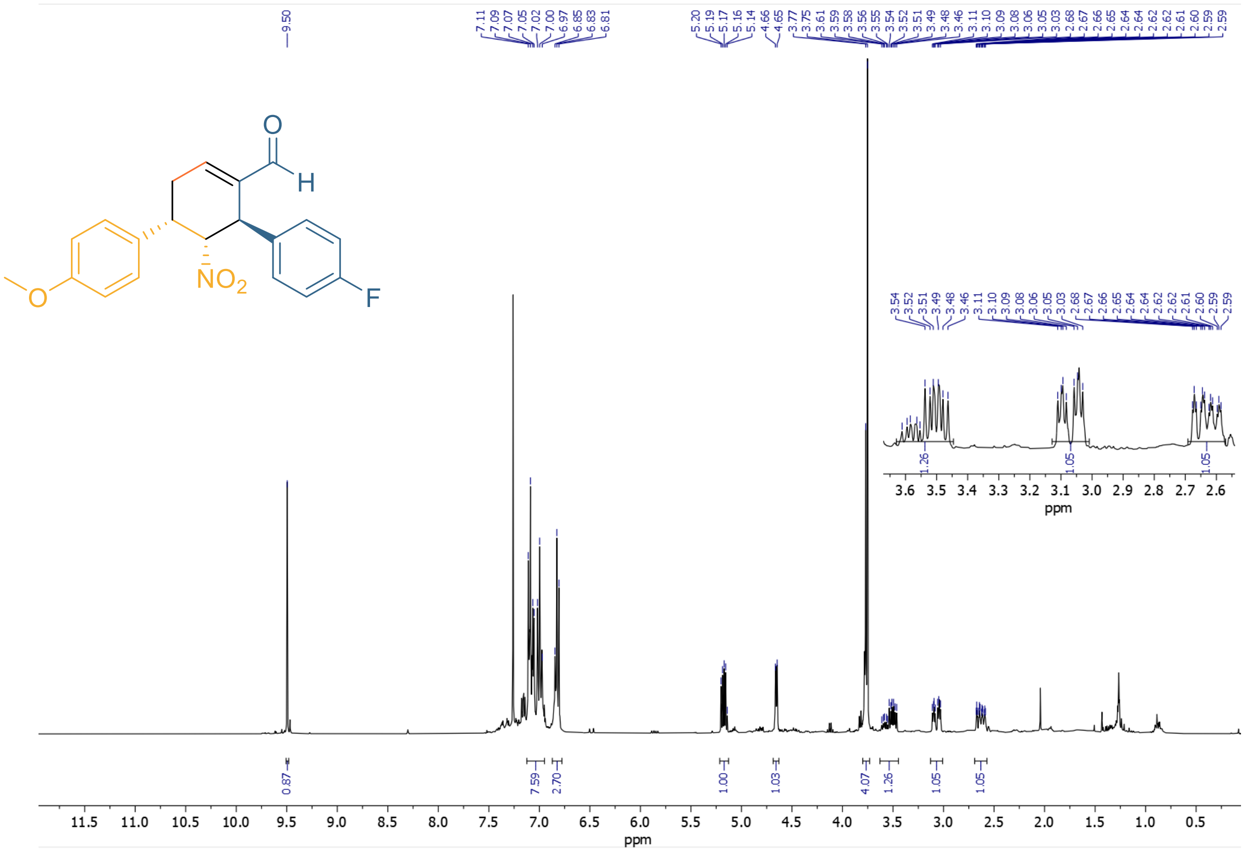

Figure S25: ^{1}H-NMR (400 MHz) spectrum of 5g' in $CDCl_3$.

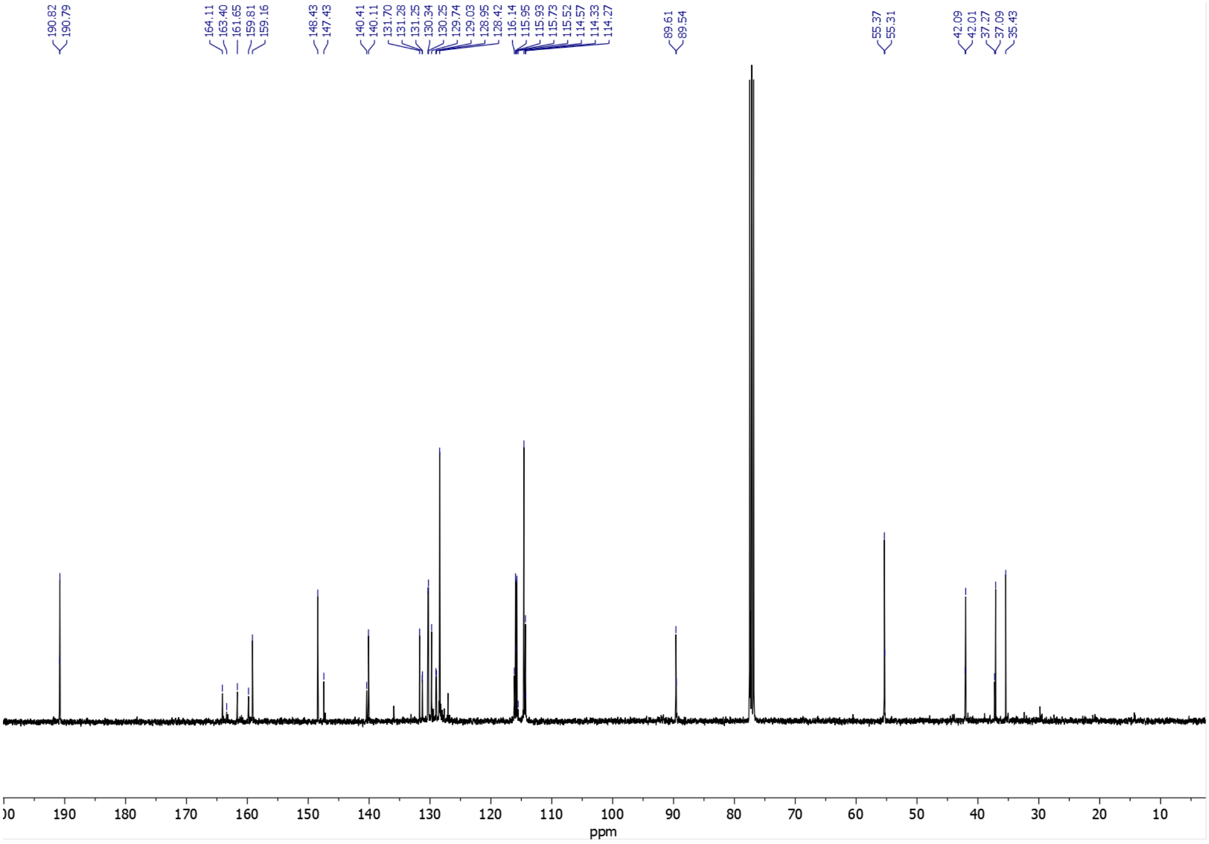

Figure S26: ^{13}C-NMR (101 MHz) spectrum of 5g' in $CDCl_3$.

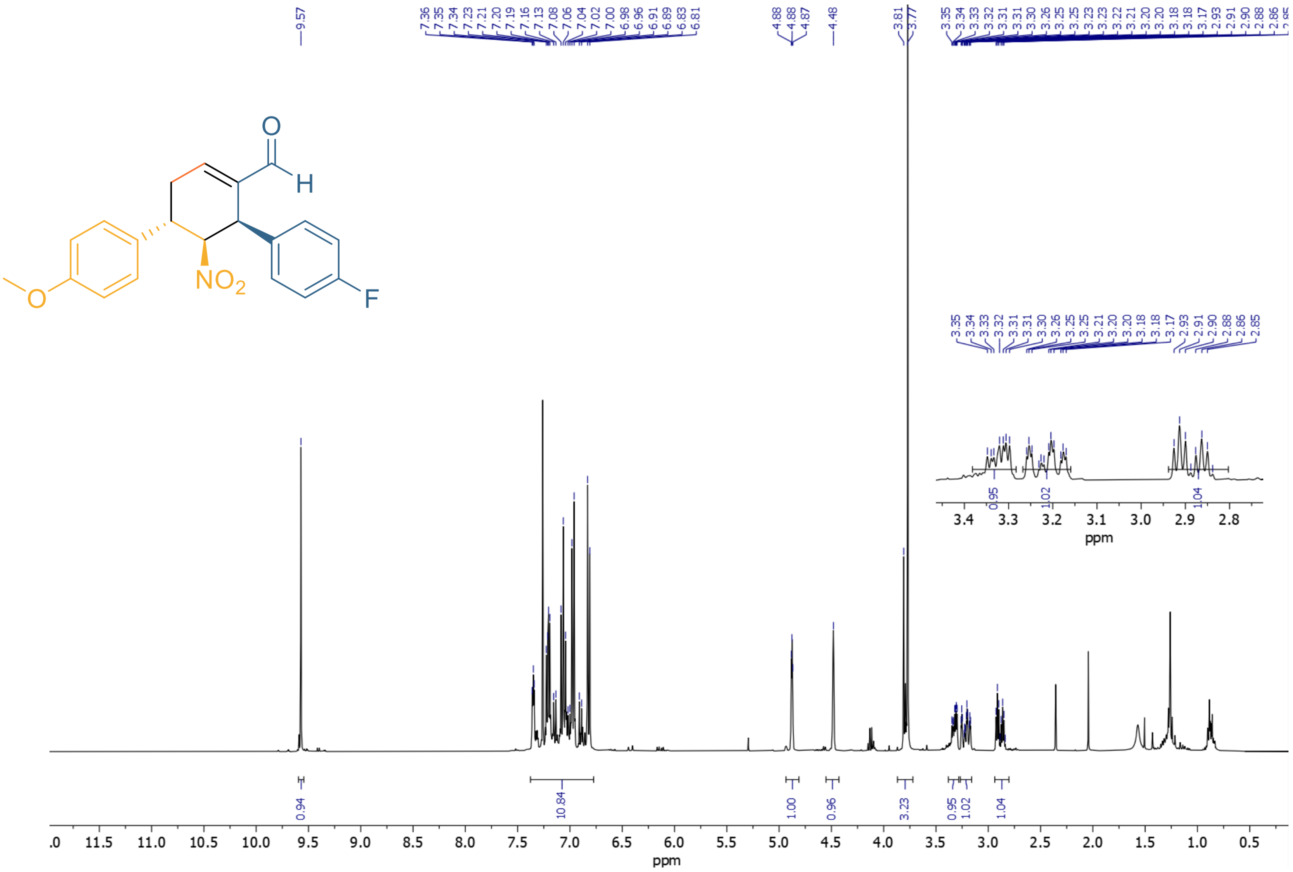

Figure S27: ^{1}H-NMR (400 MHz) spectrum of 5g'' in $CDCl_3$.

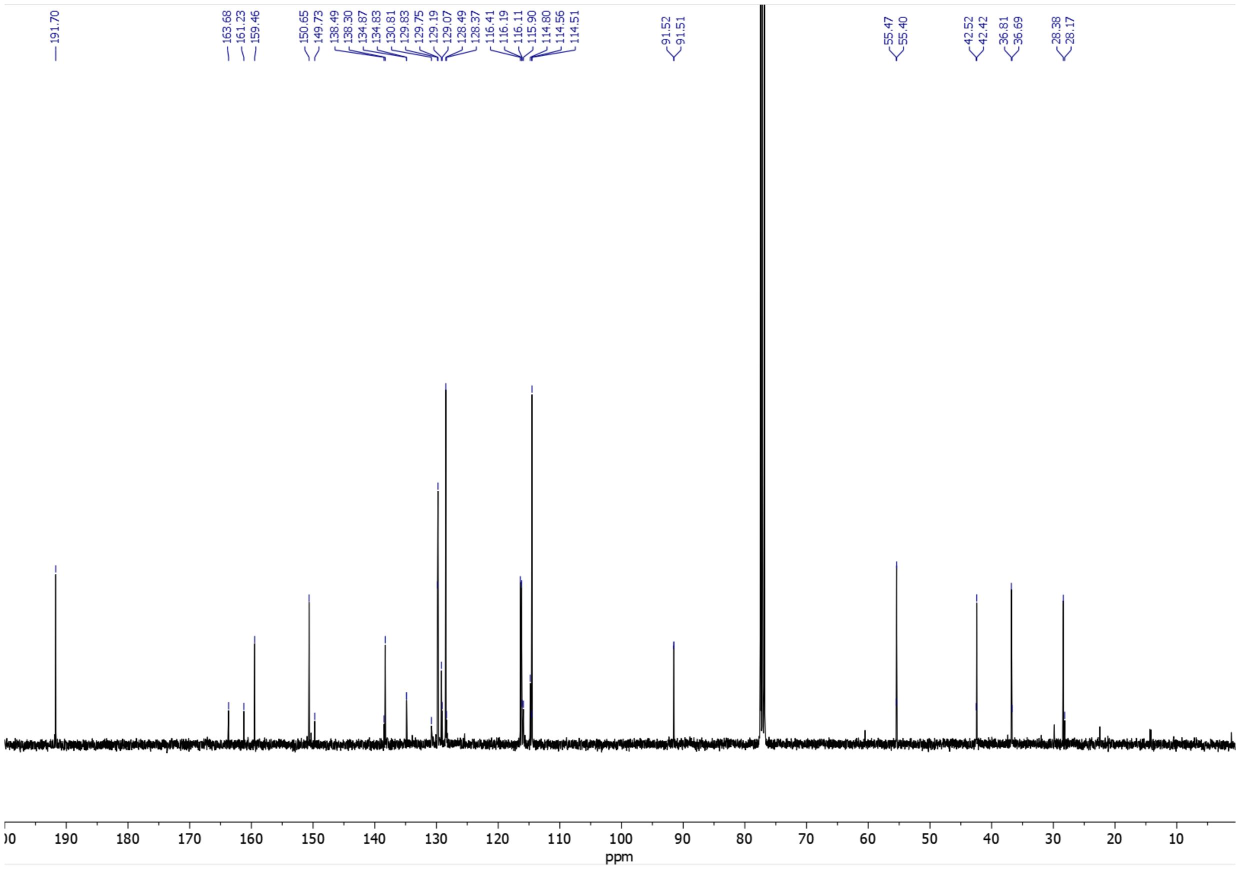

Figure S28: ^{13}C-NMR (101 MHz) spectrum of 5g'' in $CDCl_3$.

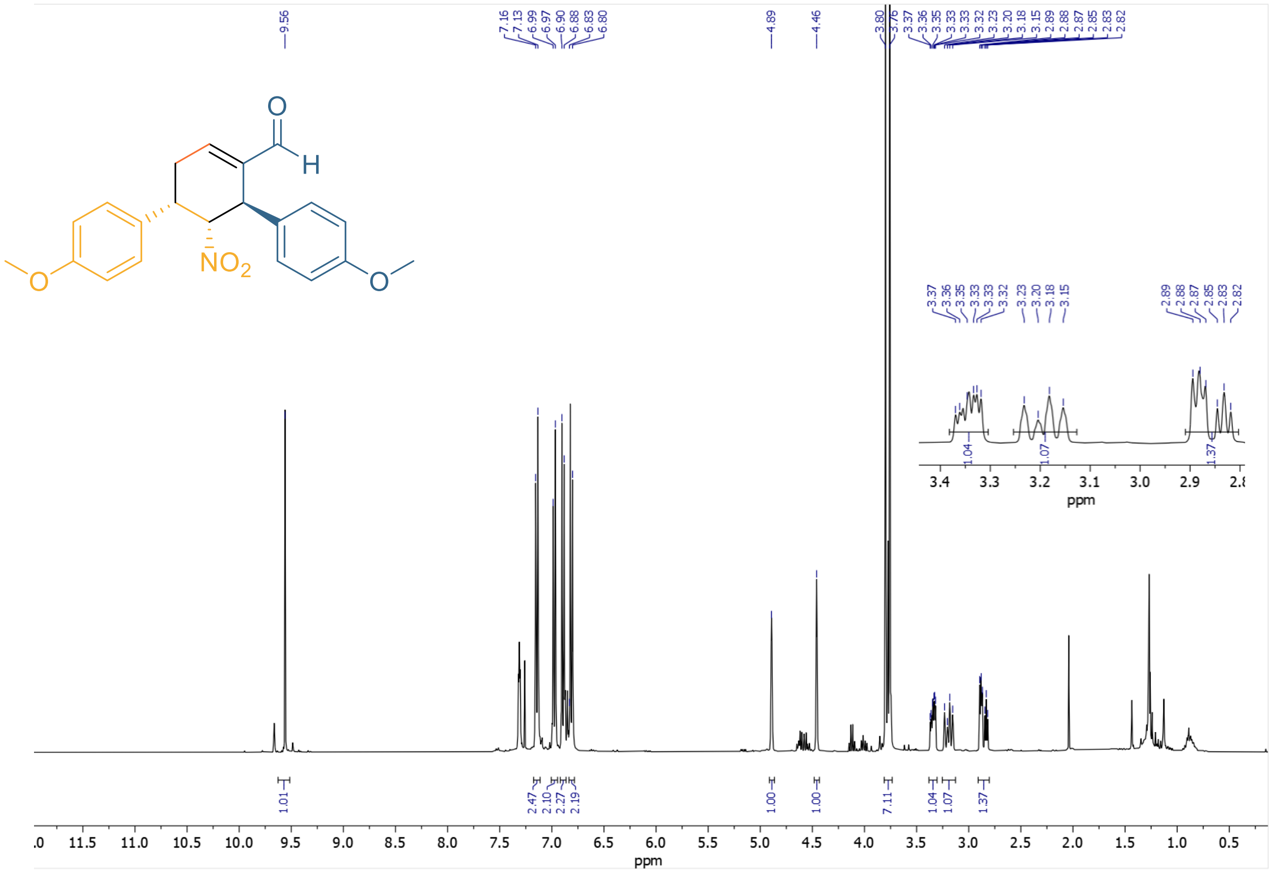

Figure S29: ^{1}H-NMR (400 MHz) spectrum of 5h' in $CDCl_3$.

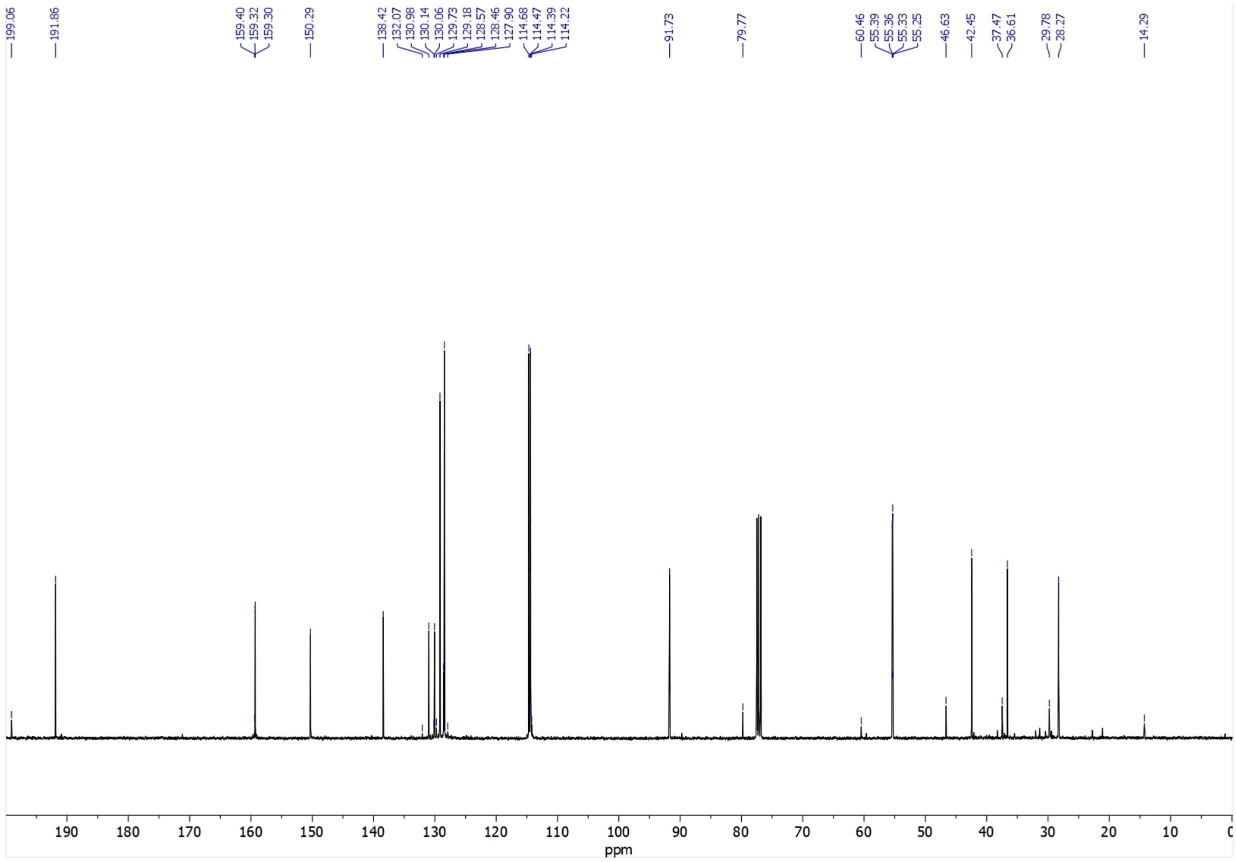

Figure S30: ^{13}C-NMR (101 MHz) spectrum of 5h' in $CDCl_3$.

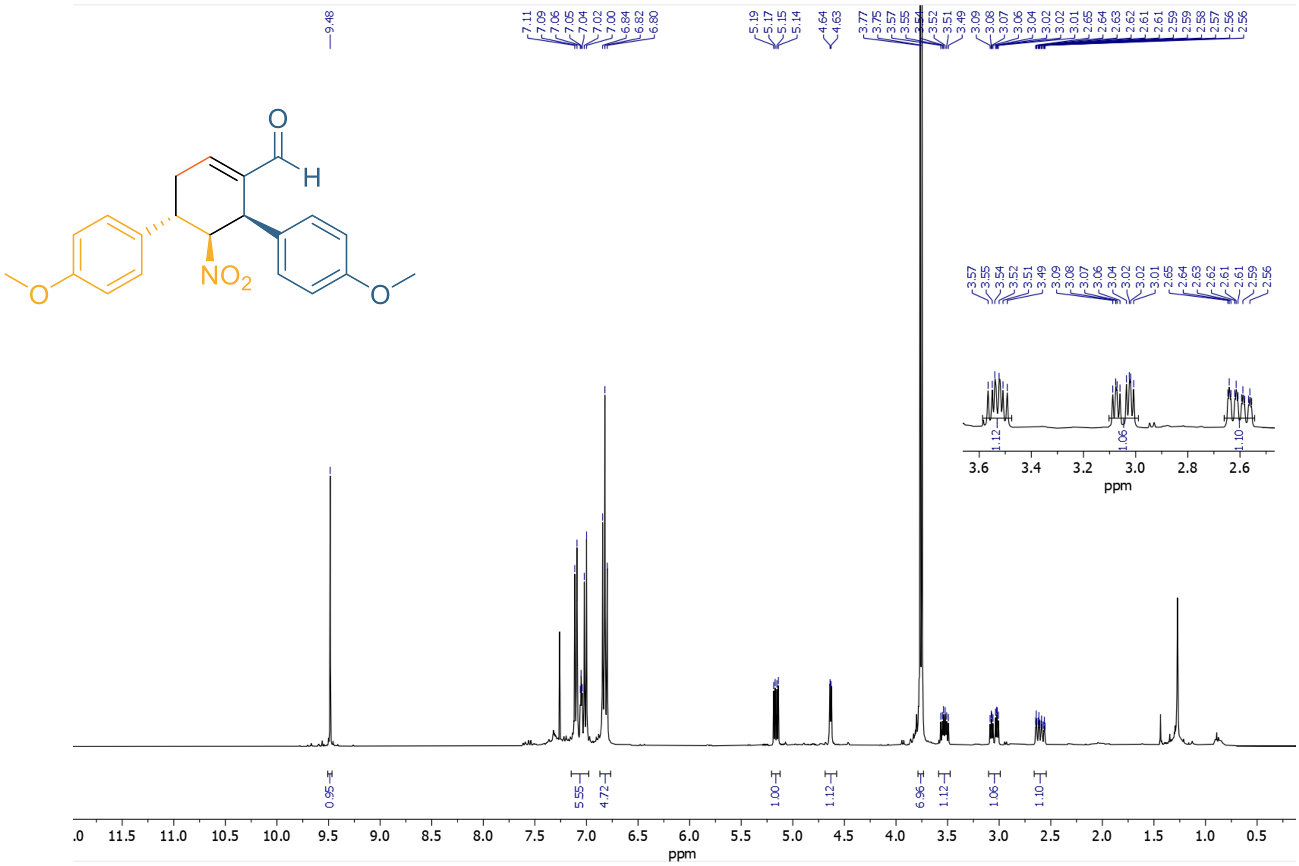

Figure S31: ¹H-NMR (400 MHz) spectrum of 5h'' in $CDCl_3$.

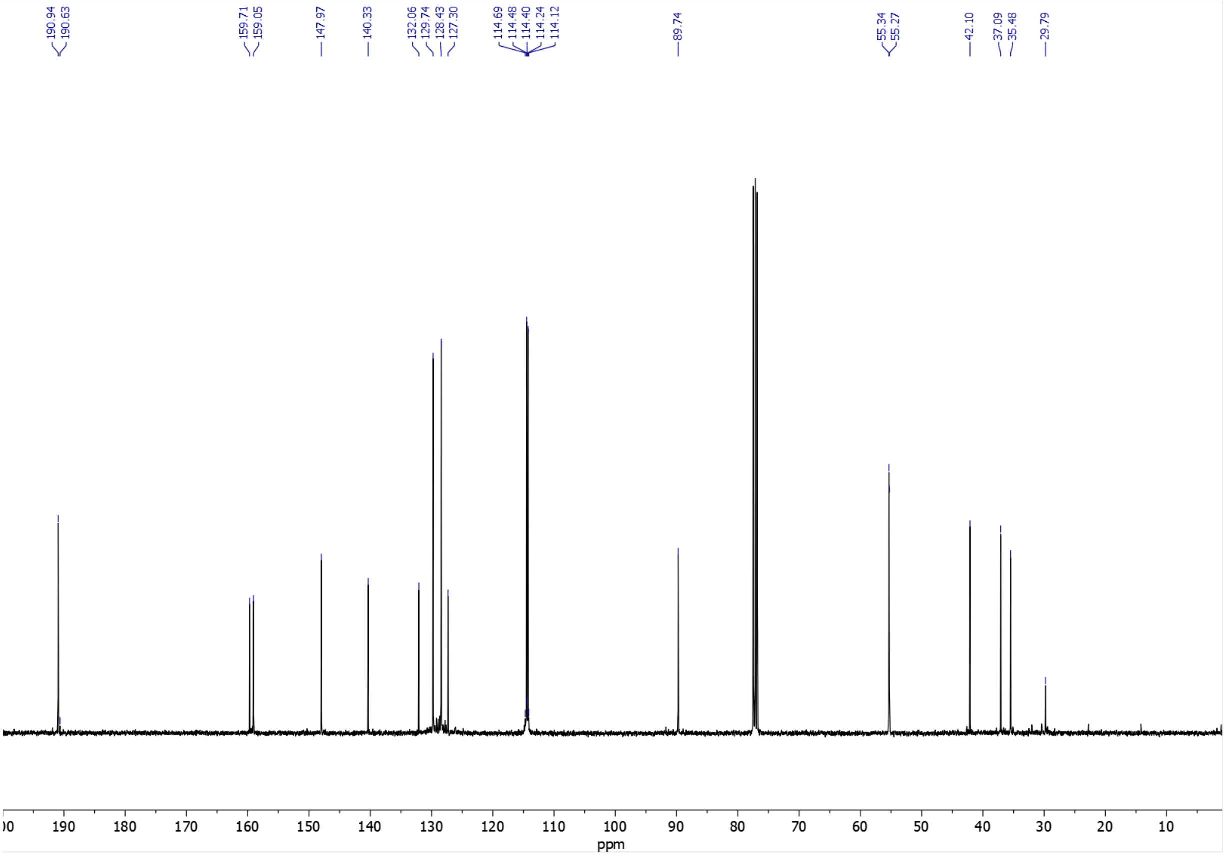

Figure S32: ¹³C-NMR (101 MHz) spectrum of 5h'' in $CDCl_3$.

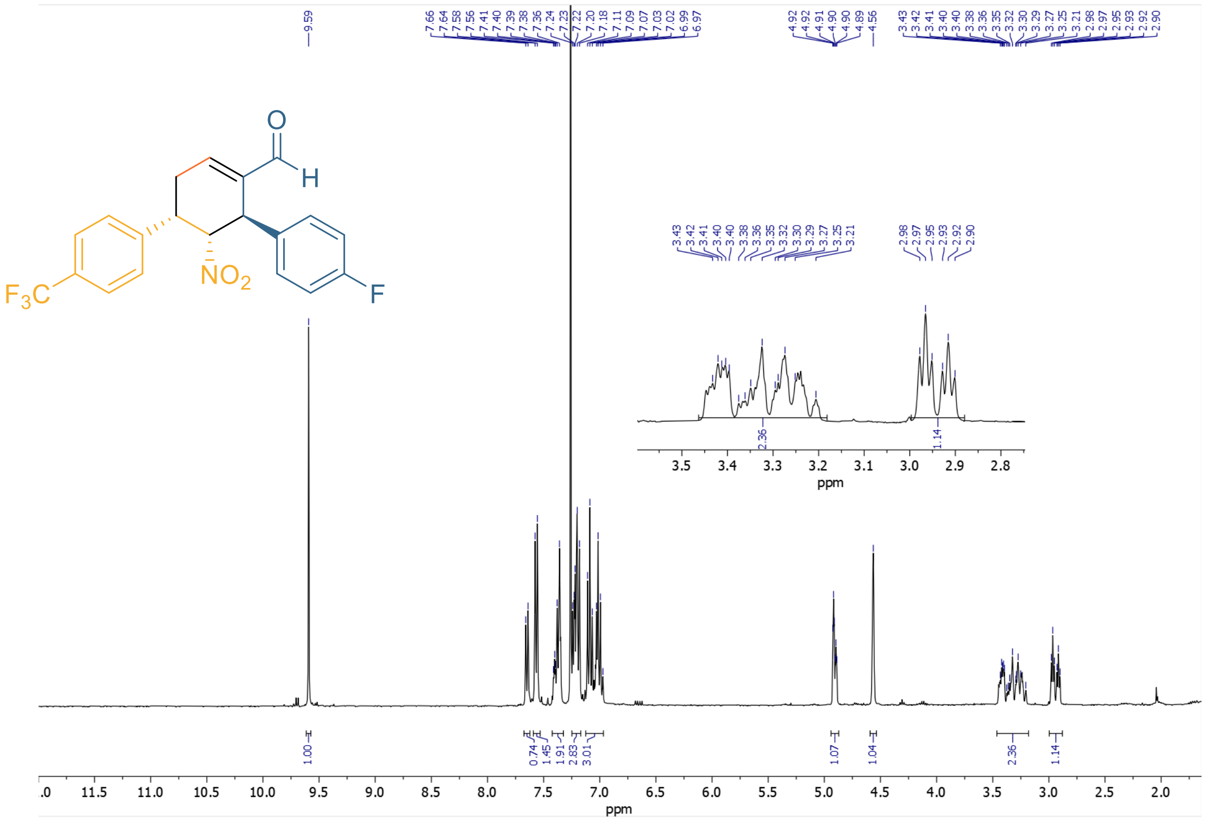

Figure S33: ^{1}H-NMR (400 MHz) spectrum of 5i' in $CDCl_3$.

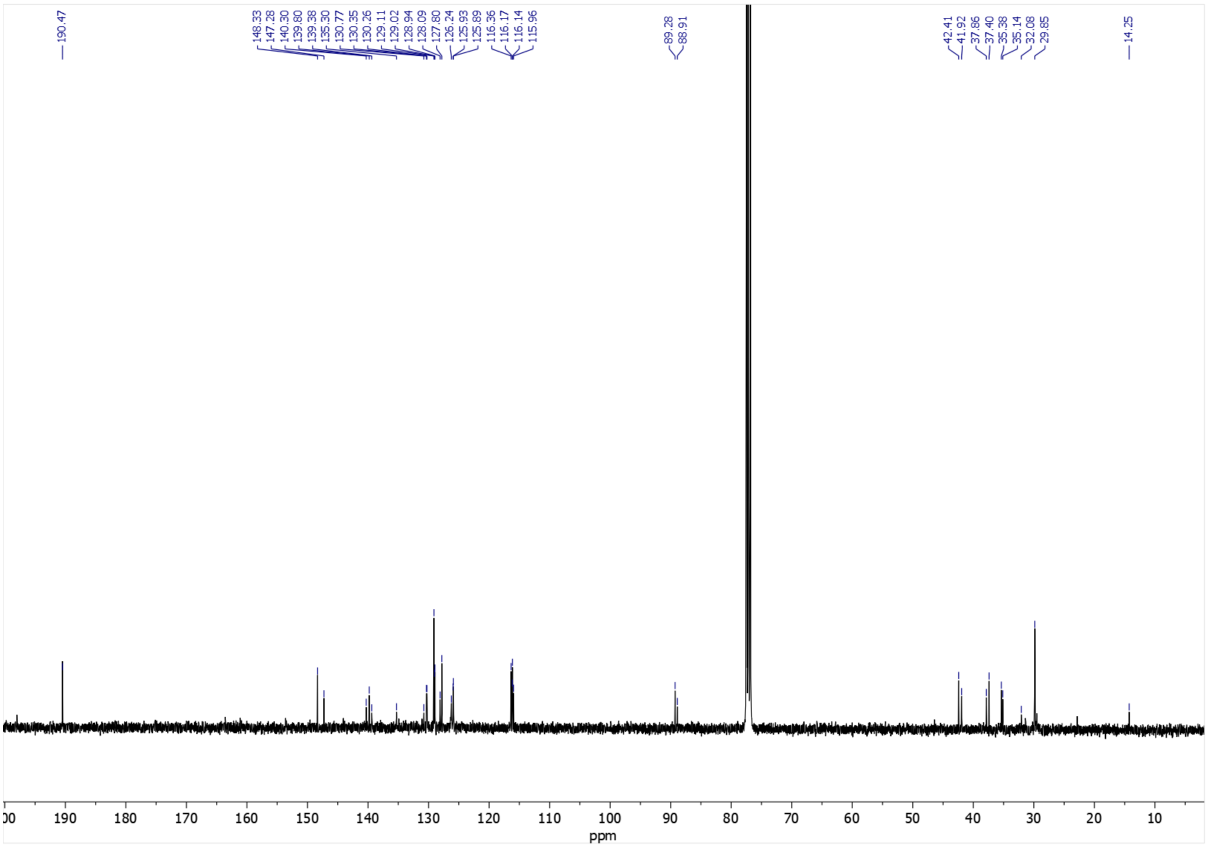

Figure S34: : ^{13}C-NMR (101 MHz) spectrum of 5i' in $CDCl_3$.

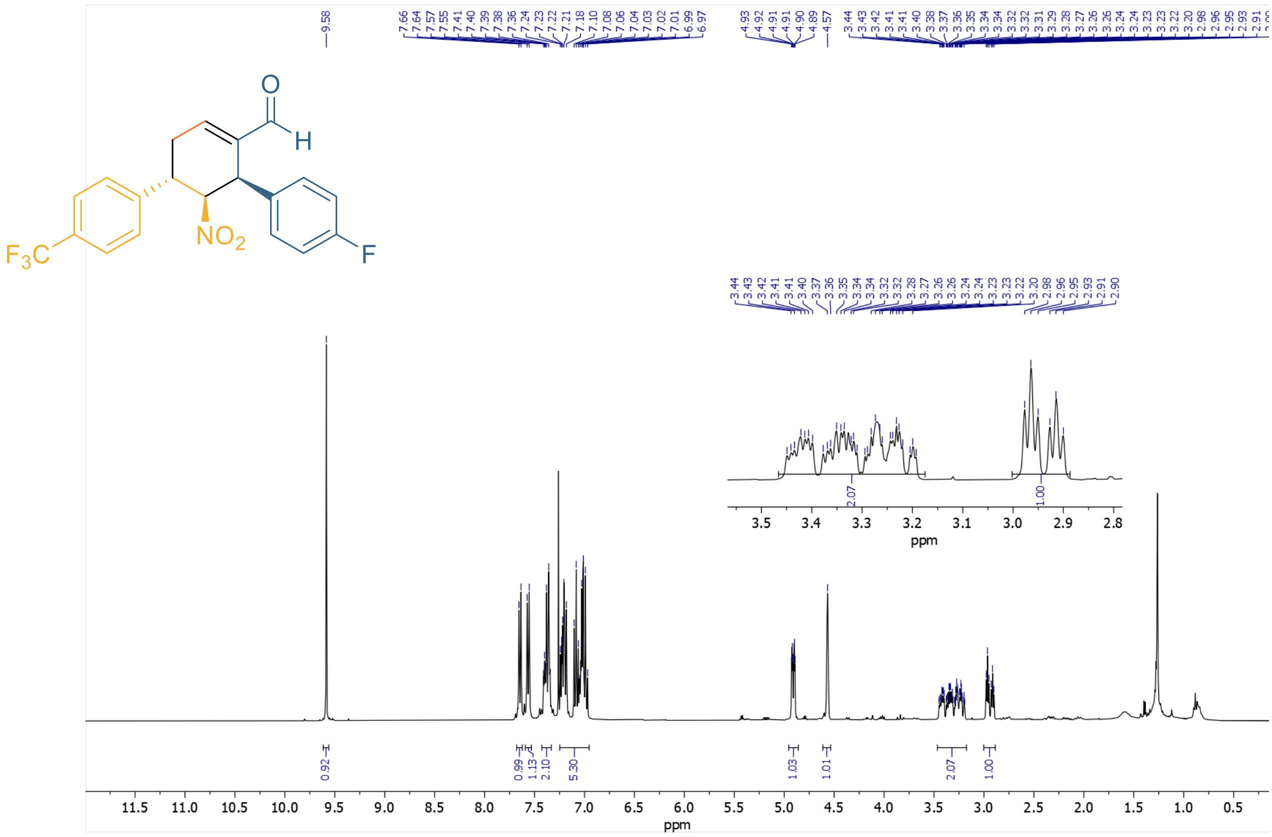

Figure S35: ^{1}H-NMR (400 MHz) spectrum of 5i'' in $CDCl_3$.

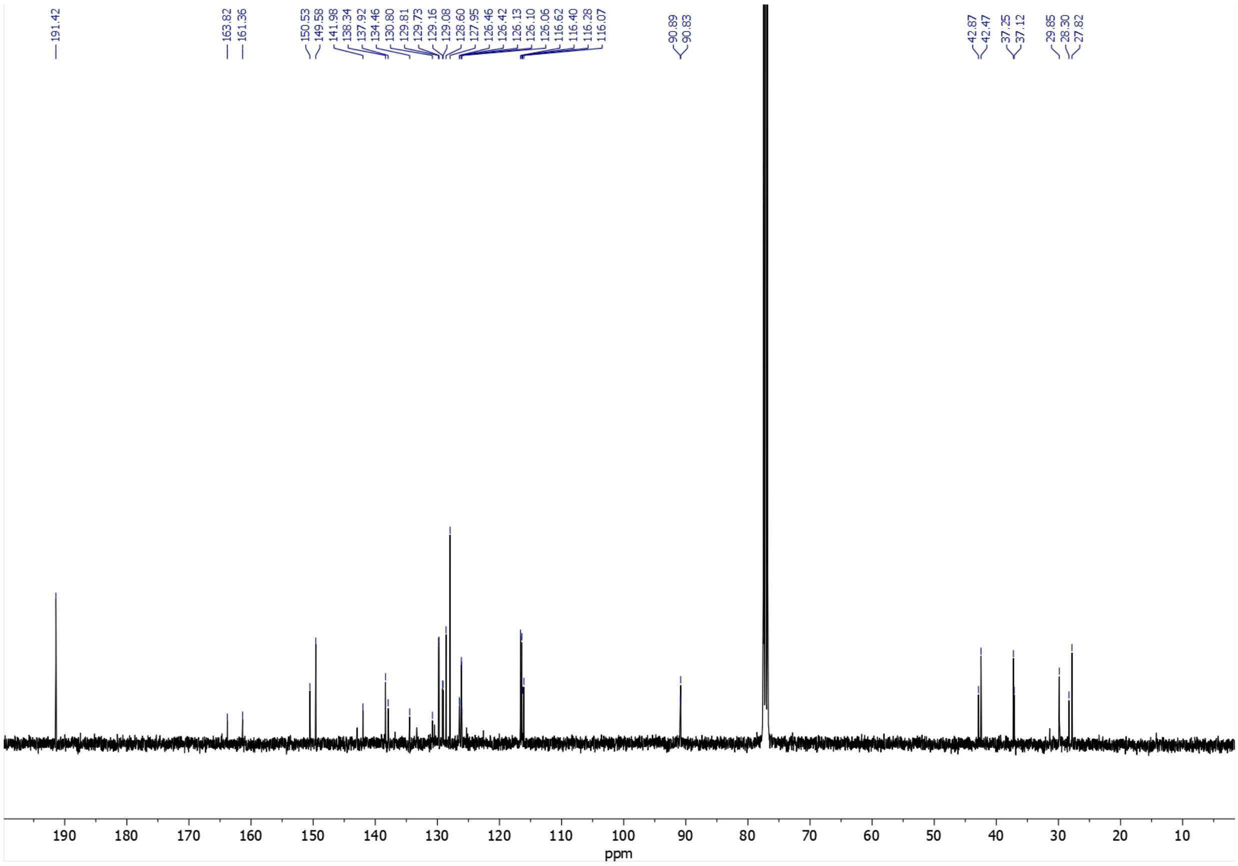

Figure S36: : ^{13}C-NMR (101 MHz) spectrum of 5i'' in $CDCl_3$.

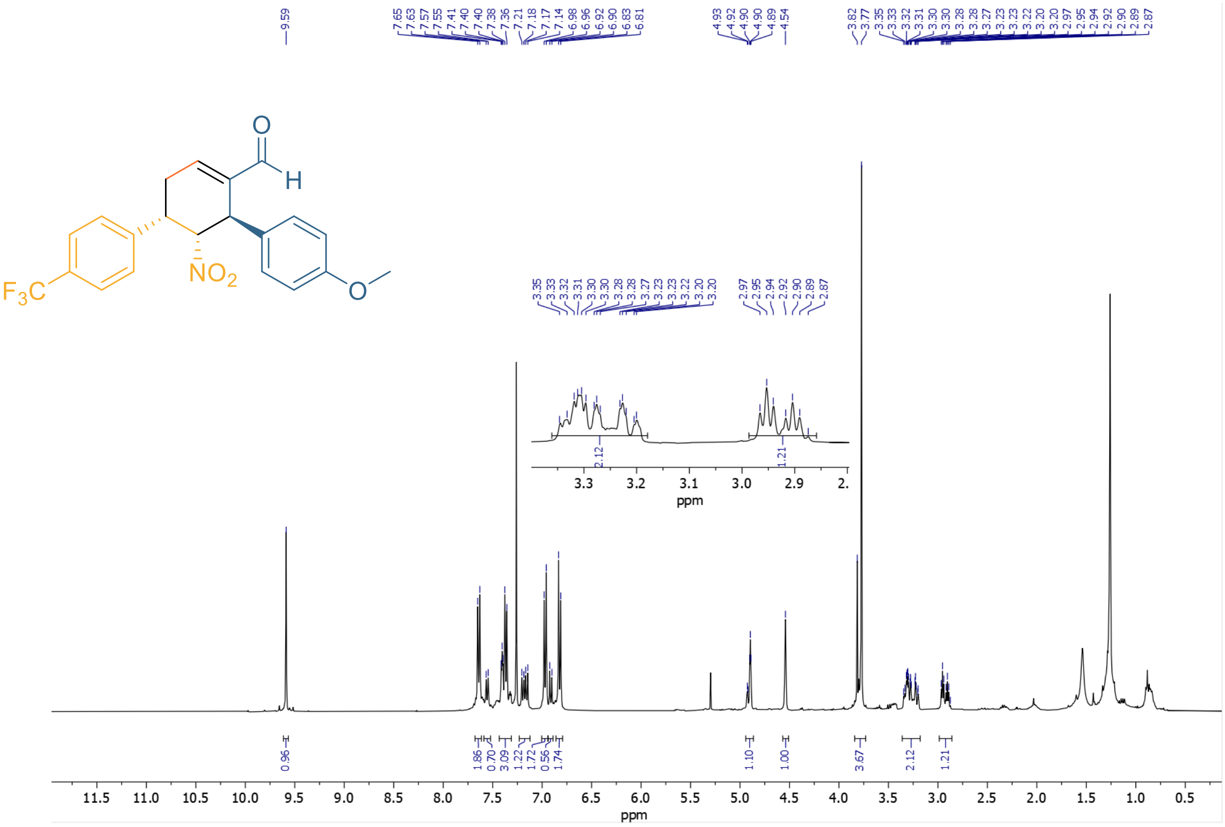

Figure S37: ^{1}H-NMR (400 MHz) spectrum of 5j’ in $CDCl_3$.

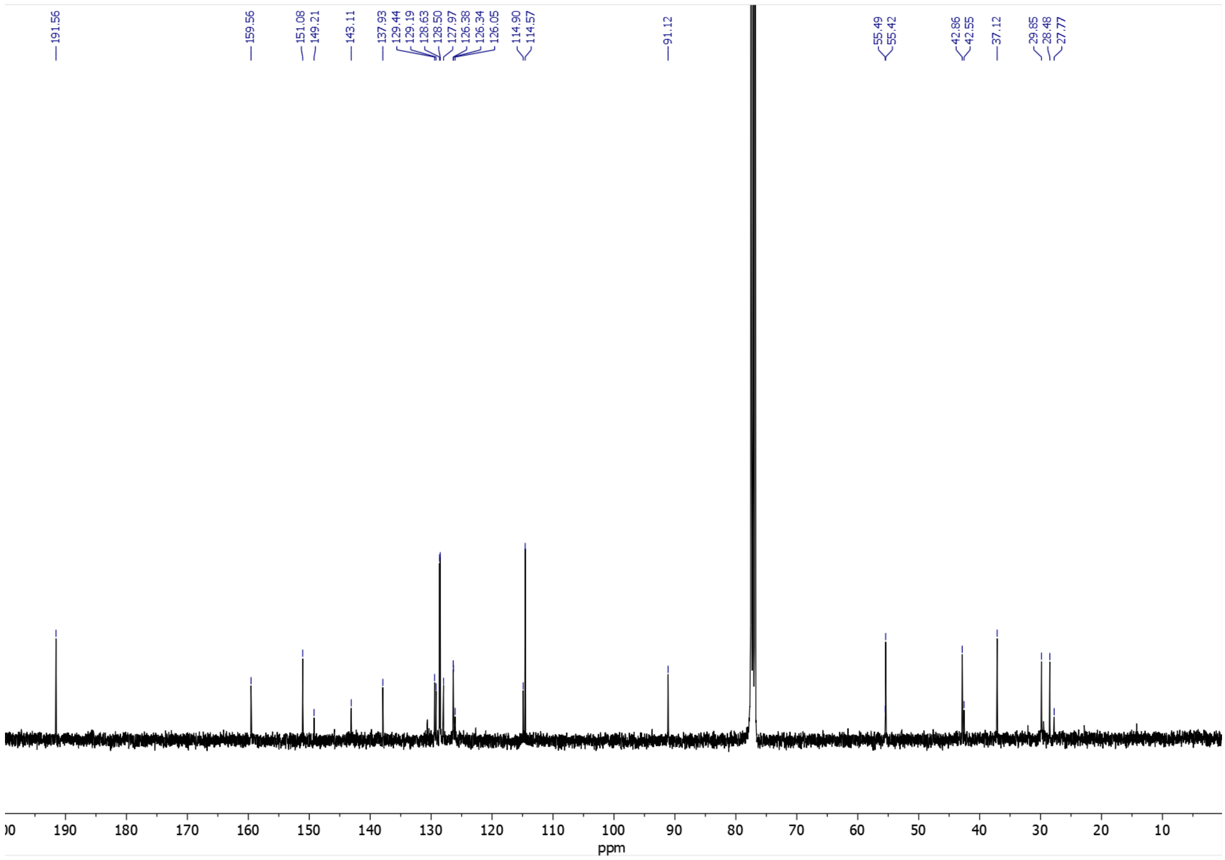

Figure S38: ^{13}C-NMR (101 MHz) spectrum of 5j’ in $CDCl_3$.

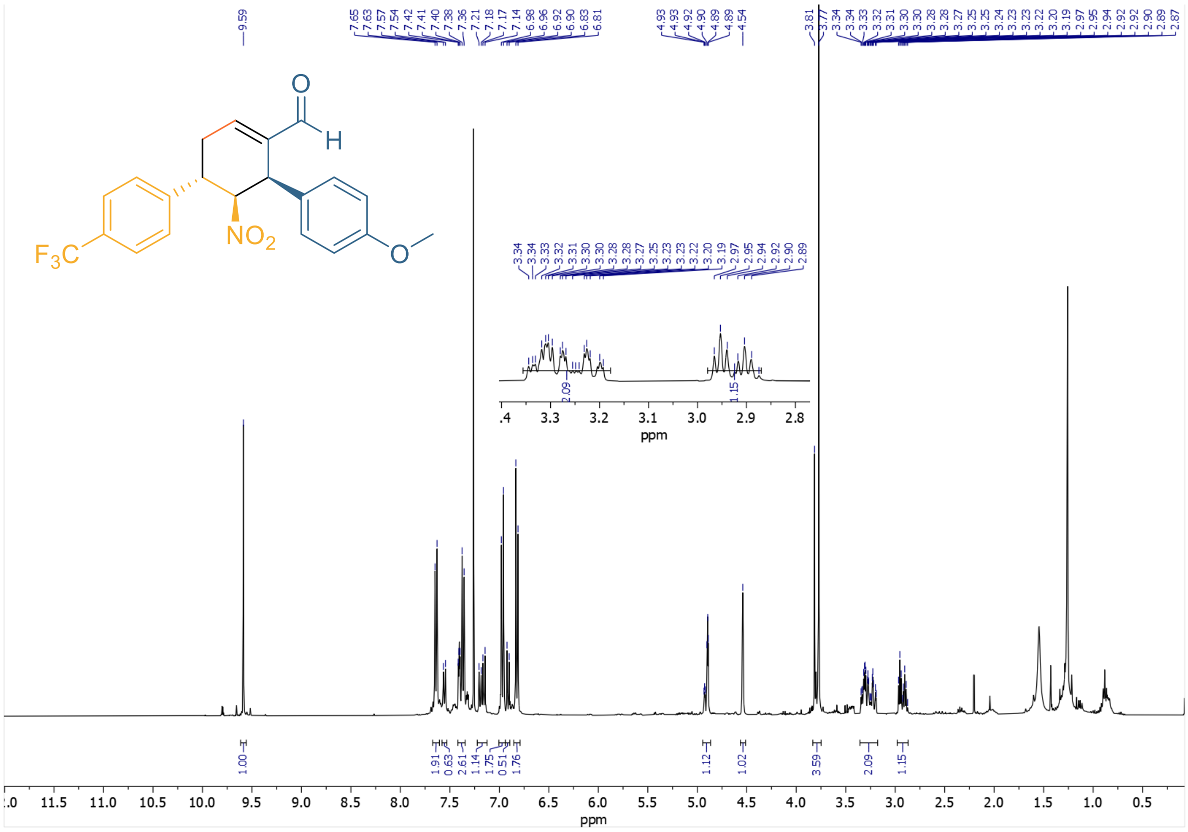

Figure S39: ^{1}H-NMR (400 MHz) spectrum of 5j'' in $CDCl_3$.

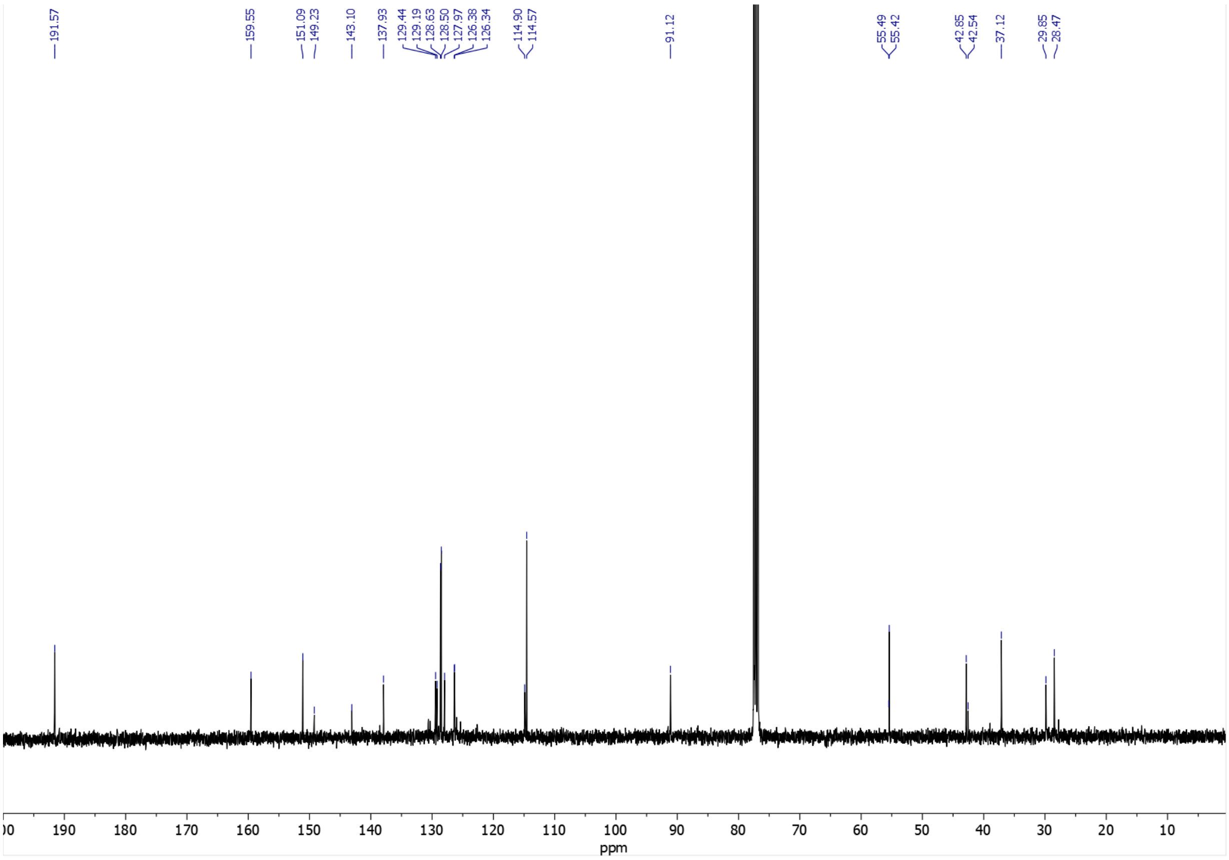

Figure S40: ^{13}C-NMR (101 MHz) spectrum of 5j'' in $CDCl_3$.

4. HPLC spectra

5a’

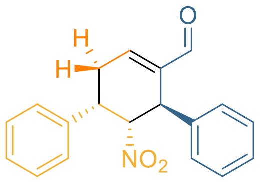

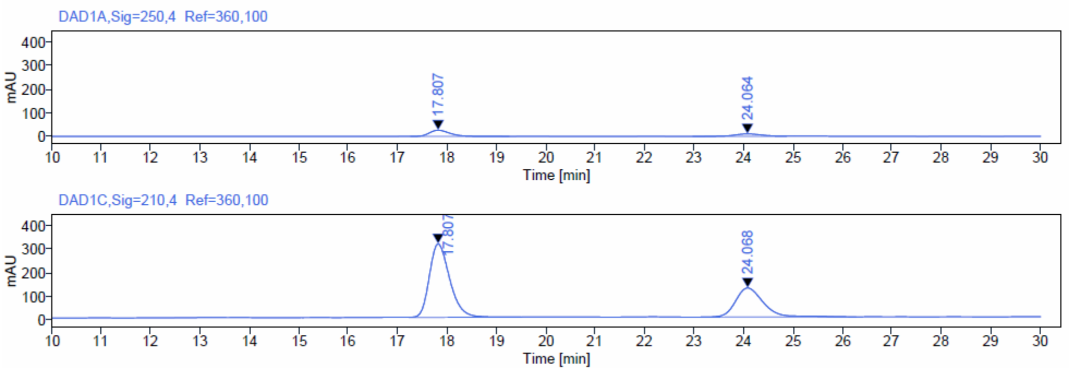

5a’ – Racemic sample

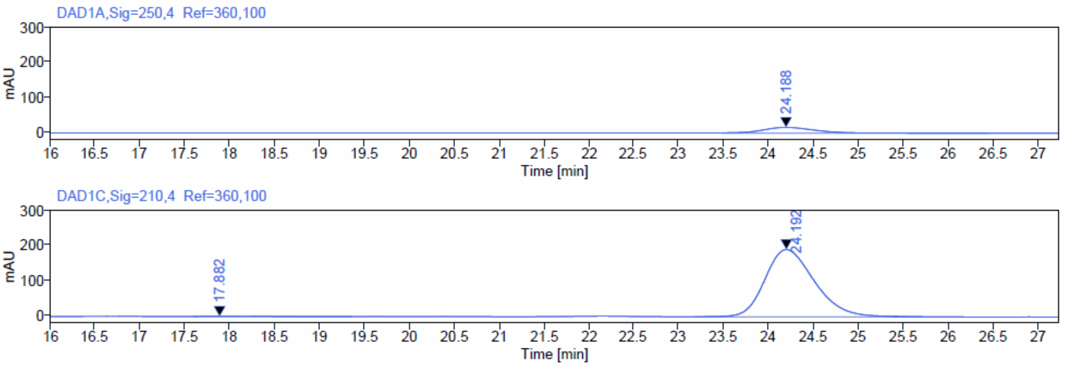

Signal: DAD1A,Sig=250,4 Ref=360,100

RT [min]	Type	Width [min]	Area	Height	Area%	Name
24.188	BB	3.35	660.58	16.22	100.00	
		Sum	660.58			

Signal: DAD1C,Sig=210,4 Ref=360,100

RT [min]	Type	Width [min]	Area	Height	Area%	Name
17.882	MM m	0.63	18.36	0.34	0.25	
24.192	BB	3.22	7378.80	191.59	99.75	
		Sum	7397.16			

5a’ - Enantioenriched sample

5a''

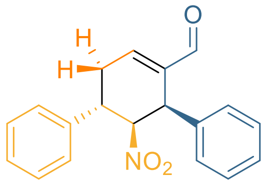

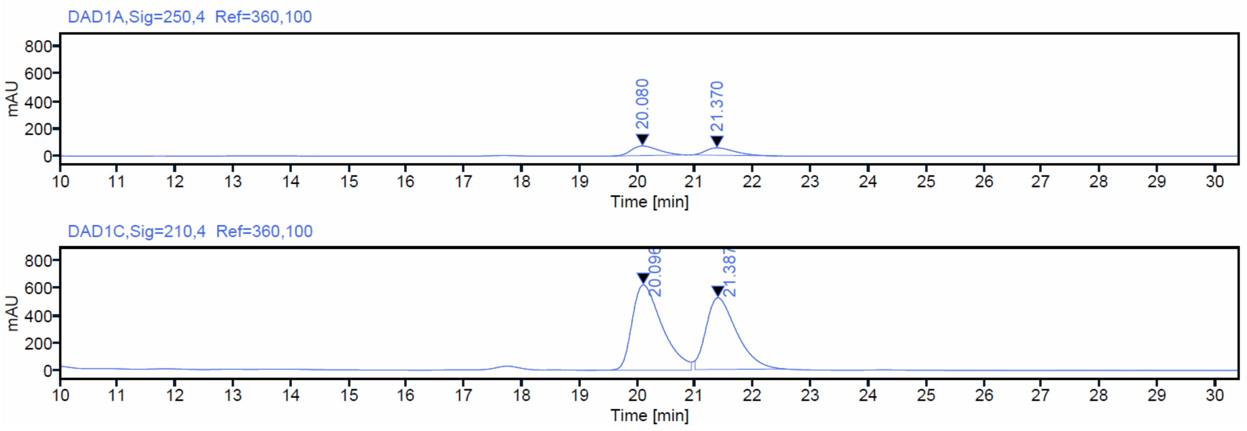

5a'' – Racemic sample

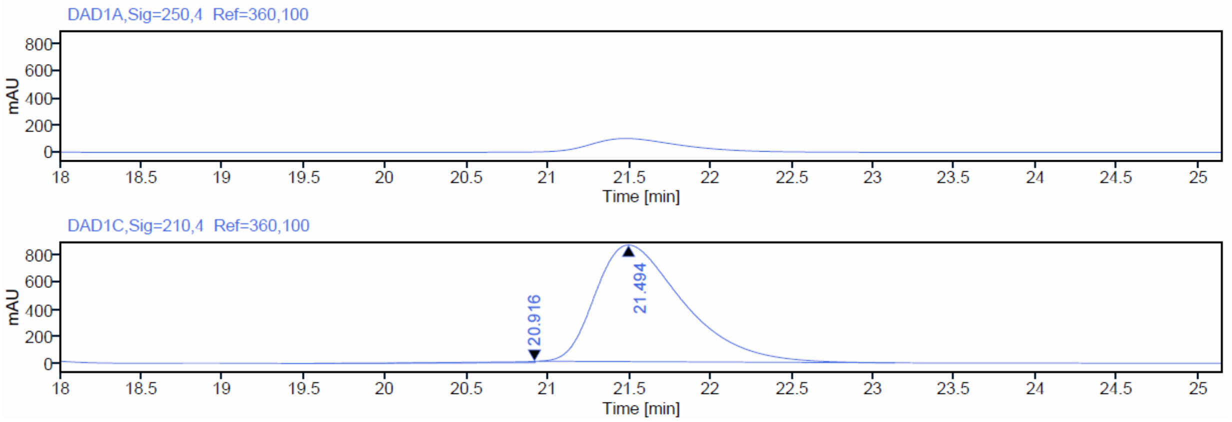

Signal: DAD1C,Sig=210,4 Ref=360,100

RT [min]	Type	Width [min]	Area	Height	Area%	Name
20.916	MM m	0.38	214.91	9.53	0.66	
21.494	MM m	0.57	32481.50	864.03	99.34	
		Sum	32696.41			

5a'' – Enantioenriched sample

5b’

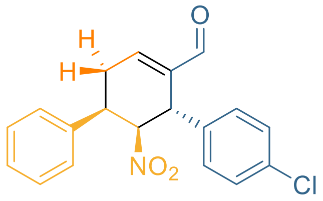

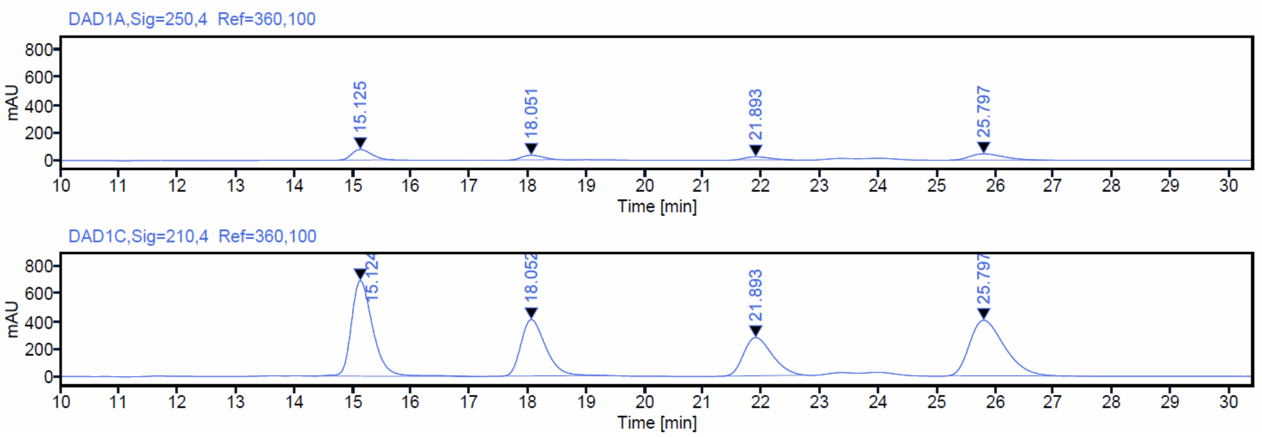

5b’ – Racemic sample

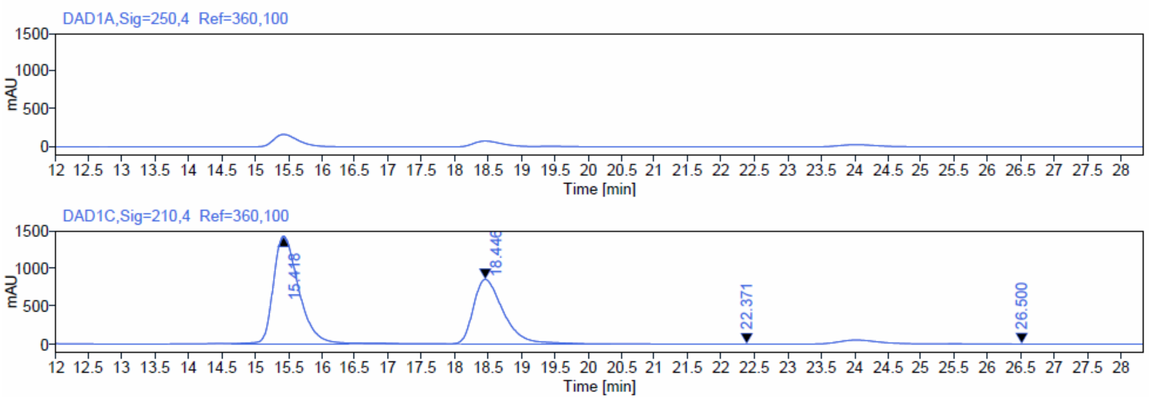

Signal: DAD1C,Sig=210,4 Ref=360,100

RT [min]	Type	Width [min]	Area	Height	Area%	Name
15.418	VB	2.90	38149.50	1426.22	58.63	
18.446	BV	2.78	26792.30	851.99	41.17	
22.371	MM n	0.22	8.25	0.45	0.01	
26.500	MM n	0.91	122.36	1.57	0.19	
		Sum	65072.41			

5b’ – Enantioenriched sample

5b''

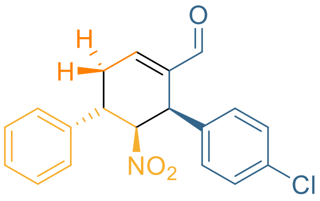

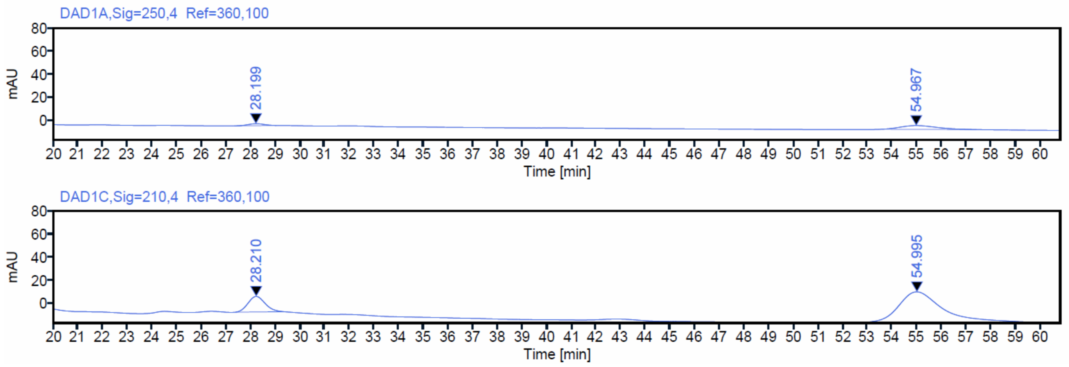

5b'' – Racemic sample

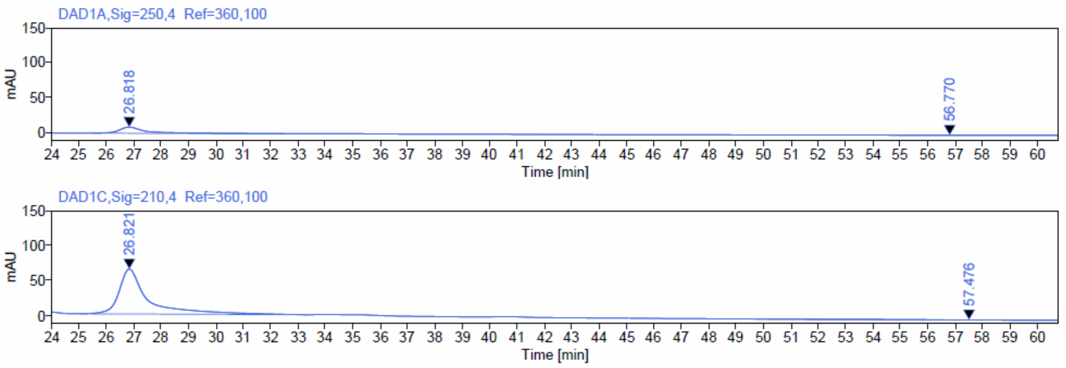

Signal: DAD1A,Sig=250,4 Ref=360,100

RT [min]	Type	Width [min]	Area	Height	Area%	Name
26.818	BB	7.05	606.35	8.72	99.01	
56.770	MM n	2.98	6.05	0.03	0.99	
		Sum	612.41			

Signal: DAD1C,Sig=210,4 Ref=360,100

RT [min]	Type	Width [min]	Area	Height	Area%	Name
26.821	BB	8.33	4757.97	64.08	99.43	
57.476	MM n	3.12	27.30	0.15	0.57	
		Sum	4785.27			

5b'' – Enantioenriched sample

5c’

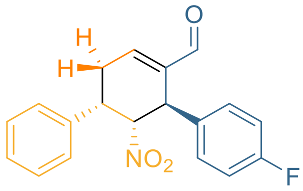

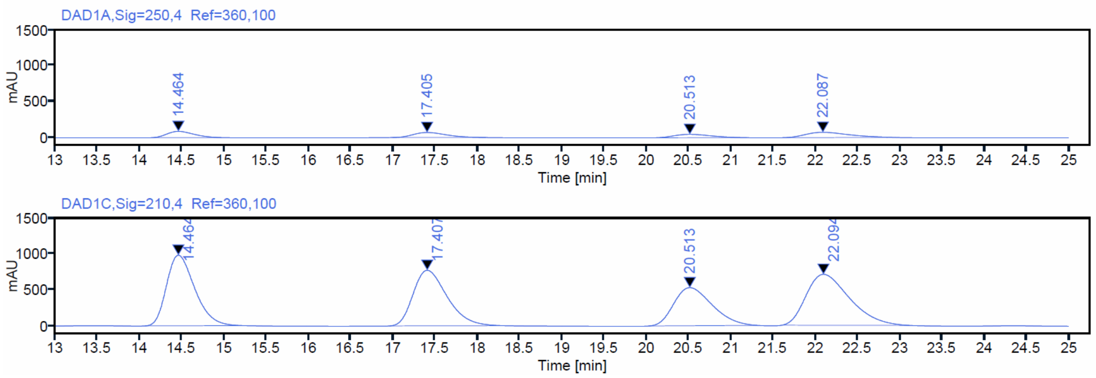

5c’ – Racemic sample

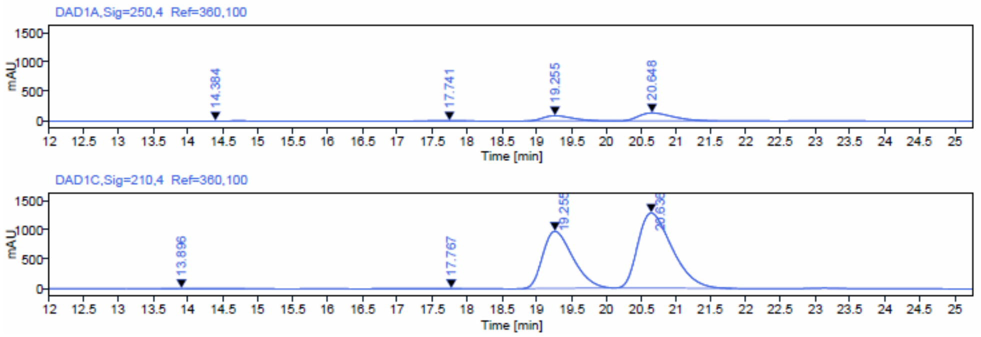

Signal: DAD1A,Sig=250,4 Ref=360,100

RT [min]	Type	Width [min]	Area	Height	Area%	Name
14.384	MM n	0.30	12.80	0.70	0.18	
17.741	MM m	0.13	9.14	1.58	0.13	
19.255	MM m	0.46	2605.63	88.39	35.75	
20.648	MM m	0.53	4661.82	136.36	63.95	
		Sum	7289.38			

Signal: DAD1C,Sig=210,4 Ref=360,100

RT [min]	Type	Width [min]	Area	Height	Area%	Name
13.896	MM m	0.28	68.29	3.53	0.09	
17.767	MM m	0.19	17.81	1.19	0.02	
19.255	MM m	0.46	29162.10	974.94	39.93	
20.636	MM m	0.53	43786.03	1281.22	59.95	
		Sum	73034.22			

5c’ - Enantioenriched sample

5c''

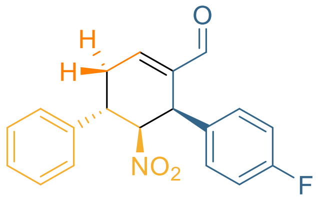

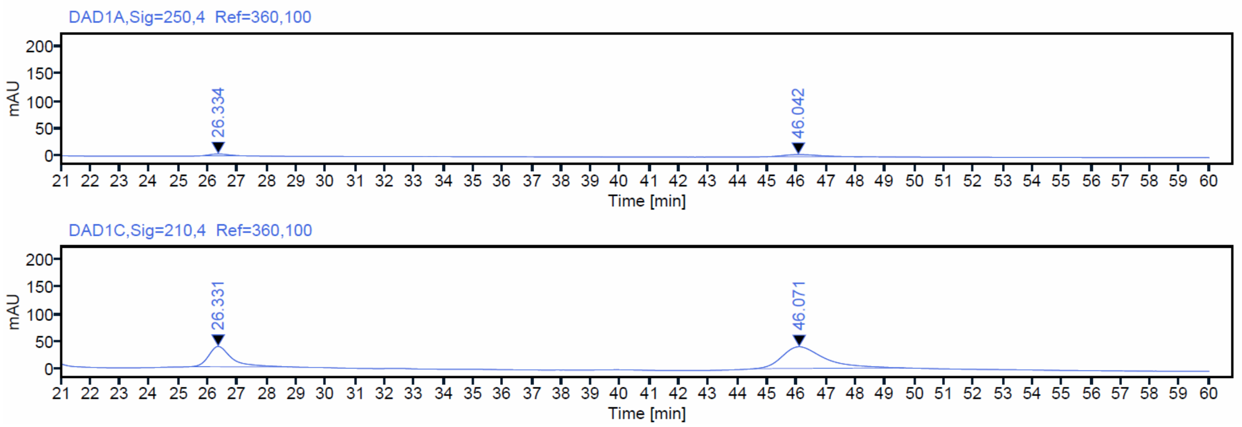

5c'' – Racemic sample

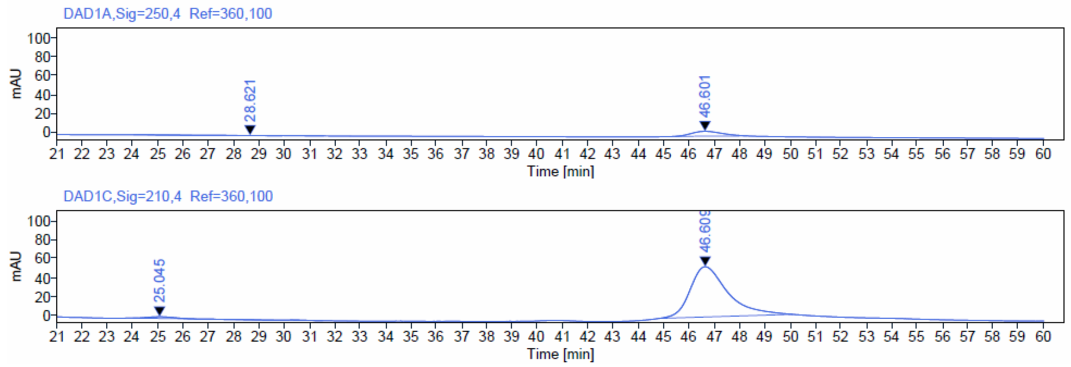

Signal: DAD1A,Sig=250,4 Ref=360,100

RT [min]	Type	Width [min]	Area	Height	Area%	Name
28.621	MM n	1.26	9.57	0.13	2.16	
46.601	MM m	0.98	434.16	5.23	97.84	
		Sum	443.73			

Signal: DAD1C,Sig=210,4 Ref=360,100

RT [min]	Type	Width [min]	Area	Height	Area%	Name
25.045	MM m	0.78	120.04	1.82	2.11	
46.609	MM m	1.39	5563.08	53.84	97.89	
		Sum	5683.12			

5c'' – Enantioenriched sample

5d’

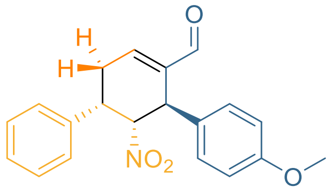

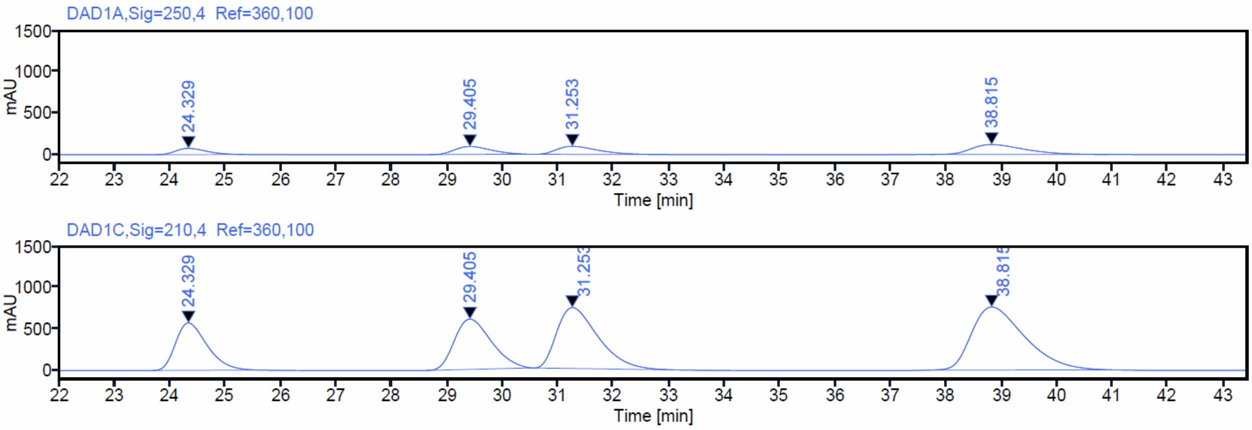

5d’ – Racemic sample

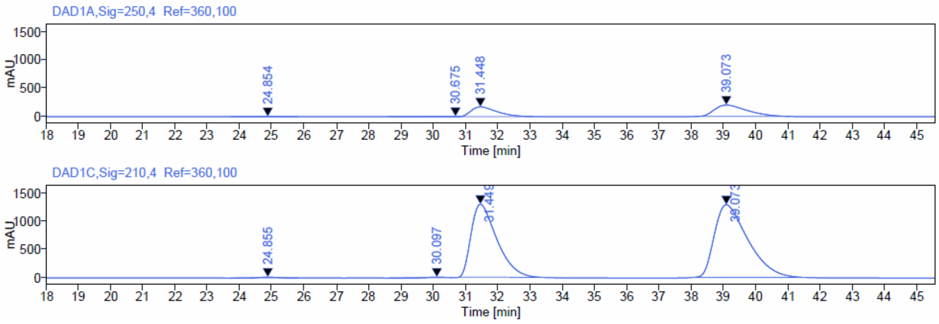

Signal: DAD1A,Sig=250,4 Ref=360,100

RT [min]	Type	Width [min]	Area	Height	Area%	Name
24.854	MM m	0.53	82.79	2.07	0.35	
30.675	MM n	0.67	82.84	2.07	0.35	
31.448	MM m	0.83	9352.40	171.93	39.37	
39.073	MM m	1.07	14234.94	203.13	59.93	
		Sum	23752.97			

Signal: DAD1C,Sig=210,4 Ref=360,100

RT [min]	Type	Width [min]	Area	Height	Area%	Name
24.855	MM m	0.58	615.48	15.40	0.37	
30.097	MM m	0.47	324.54	10.96	0.20	
31.449	MM m	0.84	71934.70	1296.17	43.52	
39.073	MM m	1.05	92400.15	1289.73	55.91	
		Sum	165274.87			

5d’ – Enantioenriched sample

5d''

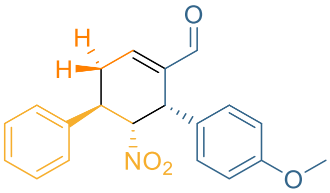

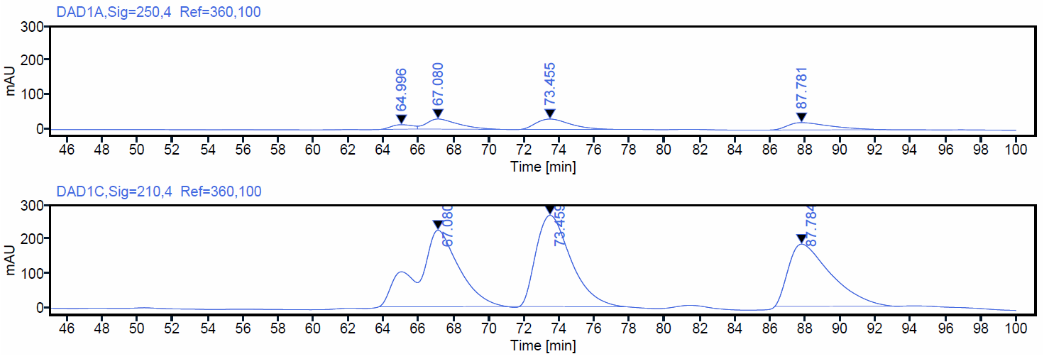

5d'' – Racemic sample

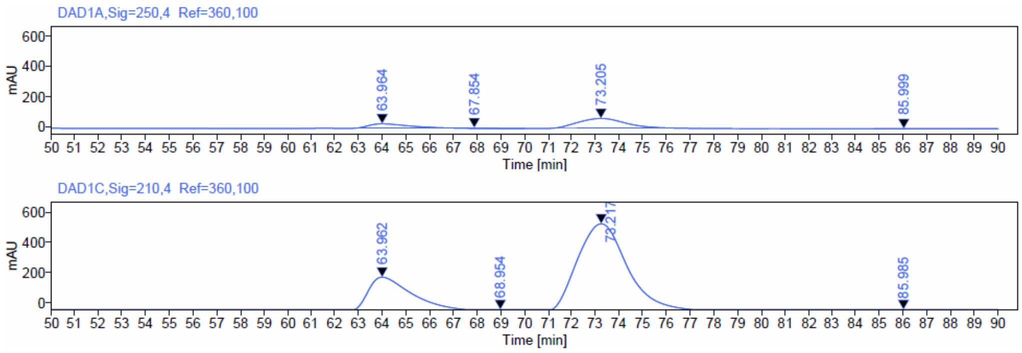

Signal: DAD1A,Sig=250,4 Ref=360,100

RT [min]	Type	Width [min]	Area	Height	Area%	Name
63.964	MM m	1.50	2989.23	27.27	26.02	
67.854	MM n	2.11	208.03	1.64	1.81	
73.205	MM m	1.88	8264.25	62.30	71.94	
85.999	MM m	1.21	26.73	0.26	0.23	
		Sum	11488.24			

Signal: DAD1C,Sig=210,4 Ref=360,100

RT [min]	Type	Width [min]	Area	Height	Area%	Name
63.962	MM m	1.82	28399.08	224.18	25.16	
68.954	MM n	1.28	141.37	1.84	0.13	
73.217	MM m	2.24	84143.74	581.97	74.54	
85.985	MM m	1.12	196.71	2.06	0.17	
		Sum	112880.90			

5d'' – Enantioenriched sample

5e’

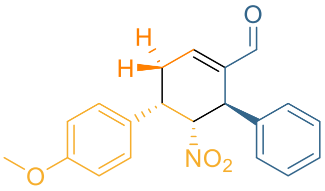

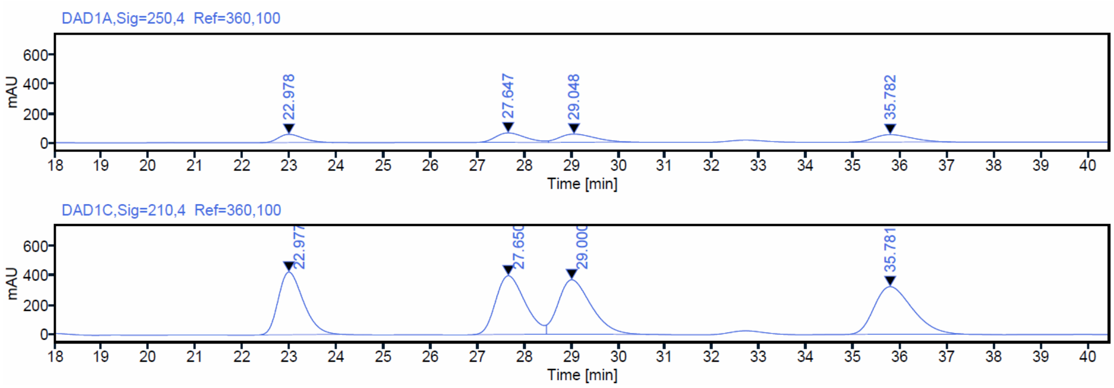

5e’ – Racemic sample

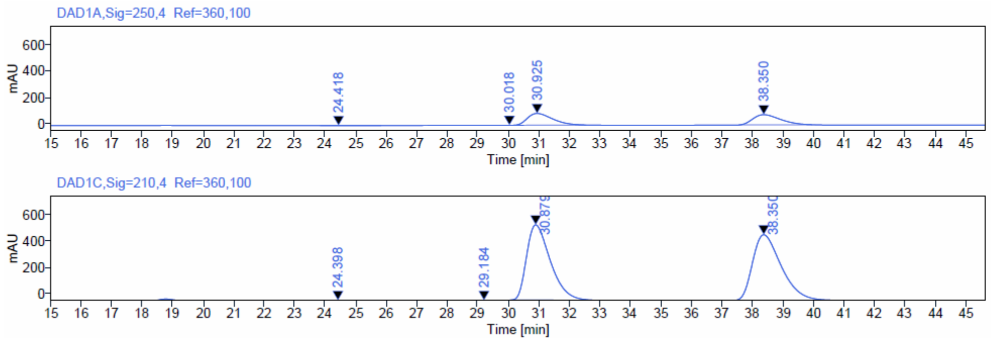

Signal: DAD1A,Sig=250,4 Ref=360,100

RT [min]	Type	Width [min]	Area	Height	Area%	Name
24.418	MM m	0.68	9.25	0.23	0.09	
30.018	MM n	0.30	3.16	0.18	0.03	
30.925	MM m	0.89	5150.29	90.07	51.30	
38.350	MM m	0.96	4877.54	78.51	48.58	
		Sum	10040.23			

Signal: DAD1C,Sig=210,4 Ref=360,100

RT [min]	Type	Width [min]	Area	Height	Area%	Name
24.398	MM m	0.54	69.44	1.53	0.11	
29.184	MM n	0.40	16.40	0.69	0.03	
30.879	MM m	0.83	31083.40	577.04	48.79	
38.350	MM m	0.99	32544.61	502.70	51.08	
		Sum	63713.84			

5e’ – Enantioenriched sample

5e''

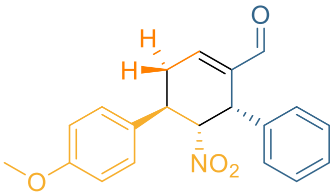

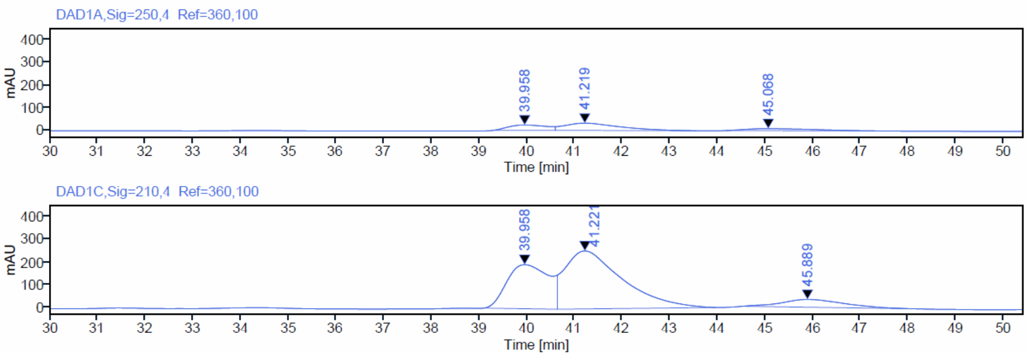

5e'' – Racemic sample

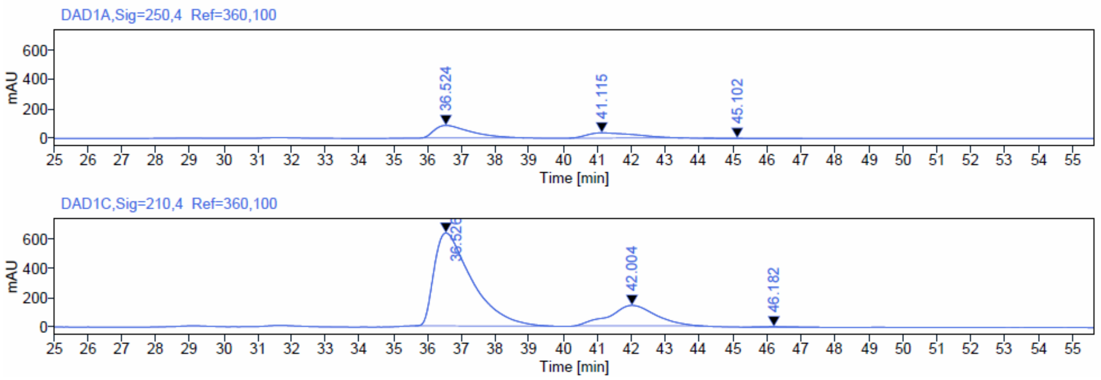

Signal: DAD1A,Sig=250,4 Ref=360,100

RT [min]	Type	Width [min]	Area	Height	Area%	Name
36.524	MM m	1.08	6373.19	86.51	63.62	
41.115	MM m	1.34	3589.18	36.15	35.83	
45.102	MM n	1.23	54.87	0.75	0.55	
		Sum	10017.25			

Signal: DAD1C,Sig=210,4 Ref=360,100

RT [min]	Type	Width [min]	Area	Height	Area%	Name
36.526	MM m	1.13	49559.88	641.56	78.49	
42.004	MM m	1.37	13392.49	140.92	21.21	
46.182	MM m	0.73	185.27	3.03	0.29	
		Sum	63137.63			

5e'' – Enantioenriched sample

5f’

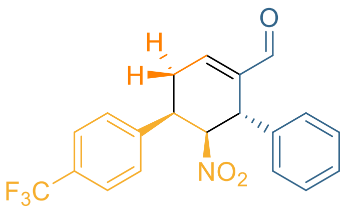

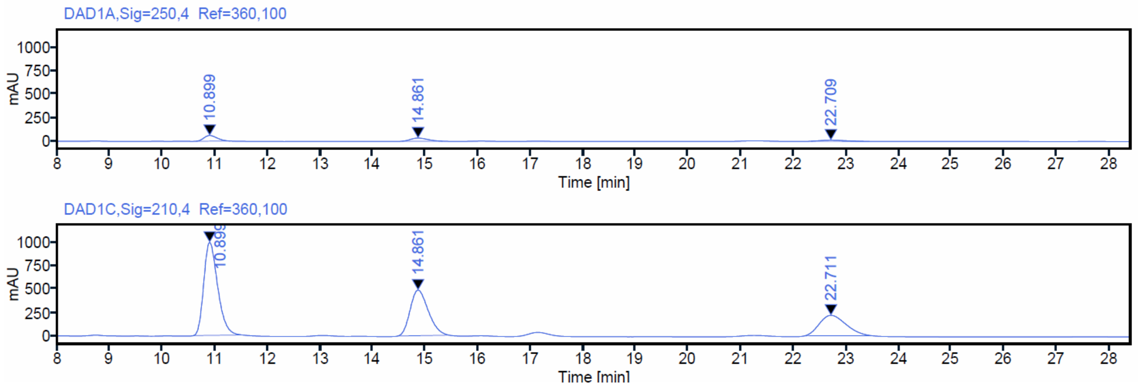

5f’ – Racemic sample

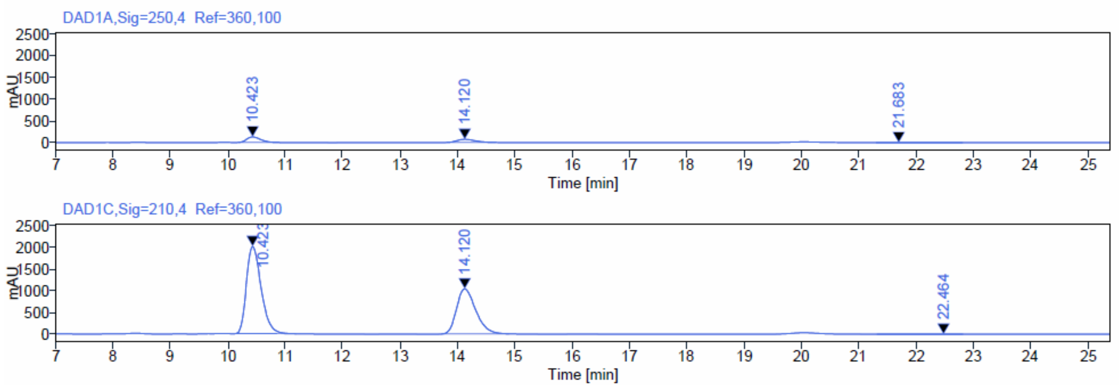

Signal: DAD1A,Sig=250,4 Ref=360,100

RT [min]	Type	Width [min]	Area	Height	Area%	Name
10.423	MM m	0.26	2173.03	129.24	57.24	
14.120	MM m	0.35	1619.06	73.25	42.64	
21.683	MM n	0.40	4.52	0.14	0.12	
		Sum	3796.60			

Signal: DAD1C,Sig=210,4 Ref=360,100

RT [min]	Type	Width [min]	Area	Height	Area%	Name
10.423	MM m	0.28	35949.17	2017.81	59.77	
14.120	MM m	0.36	24163.00	1043.40	40.17	
22.464	MM n	1.19	32.82	0.32	0.05	
		Sum	60144.99			

5f’ – Enantioenriched sample

5f''

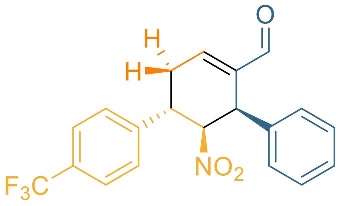

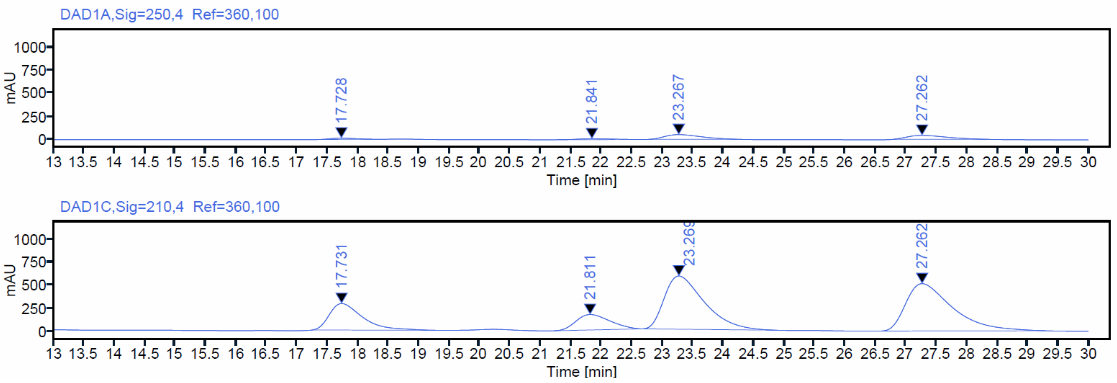

5f'' – Racemic sample

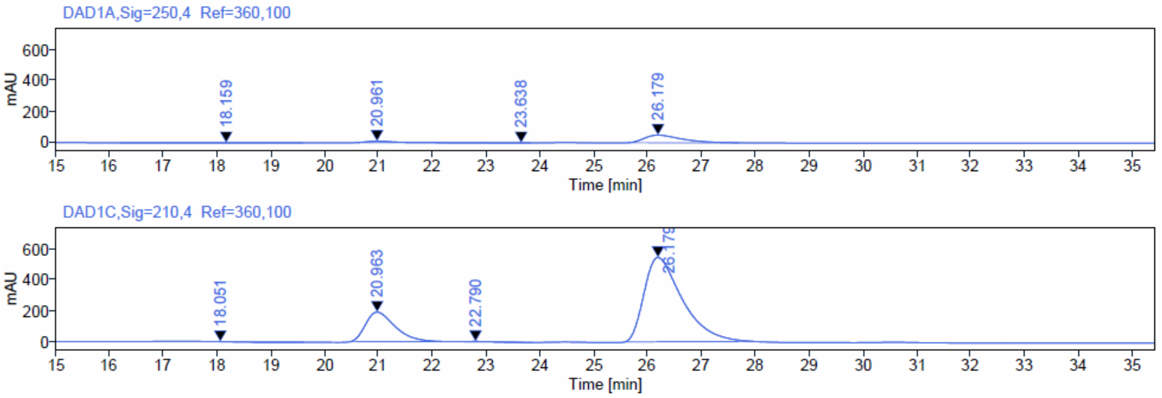

Signal: DAD1A,Sig=250,4 Ref=360,100

RT [min]	Type	Width [min]	Area	Height	Area%	Name
18.159	MM m	0.73	34.55	0.56	1.26	
20.961	MM m	0.51	310.76	9.40	11.35	
23.638	MM n	1.70	27.98	0.19	1.02	
26.179	MM m	0.71	2365.49	49.64	86.37	
		Sum	2738.78			

Signal: DAD1C,Sig=210,4 Ref=360,100

RT [min]	Type	Width [min]	Area	Height	Area%	Name
18.051	MM m	0.34	46.53	2.31	0.13	
20.963	MM m	0.58	7372.88	193.62	21.29	
22.790	MM m	0.40	92.00	2.85	0.27	
26.179	MM m	0.74	27120.62	551.02	78.31	
		Sum	34632.03			

5f'' – Enantioenriched sample

5g'

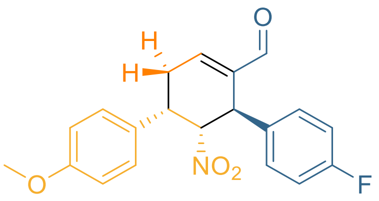

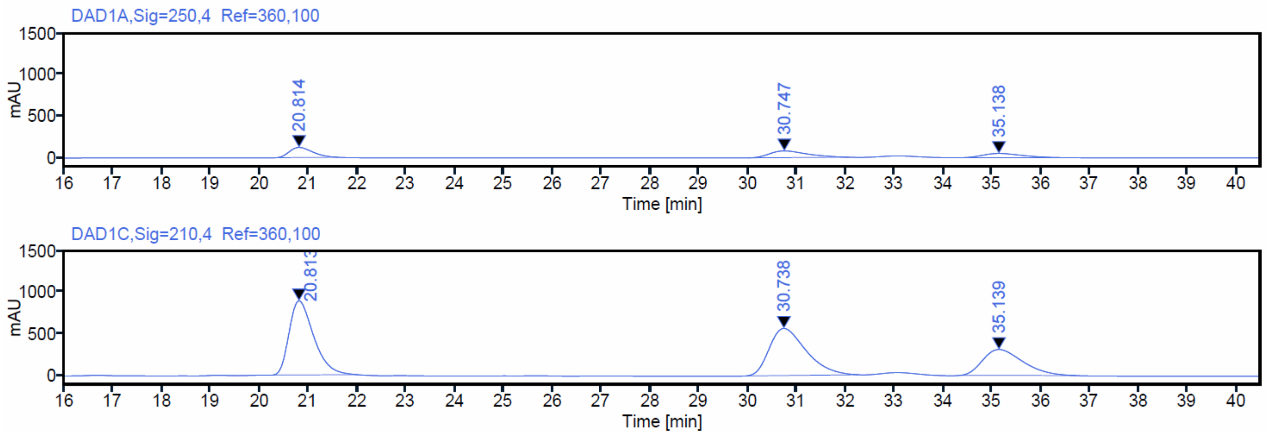

5g' – Racemic sample

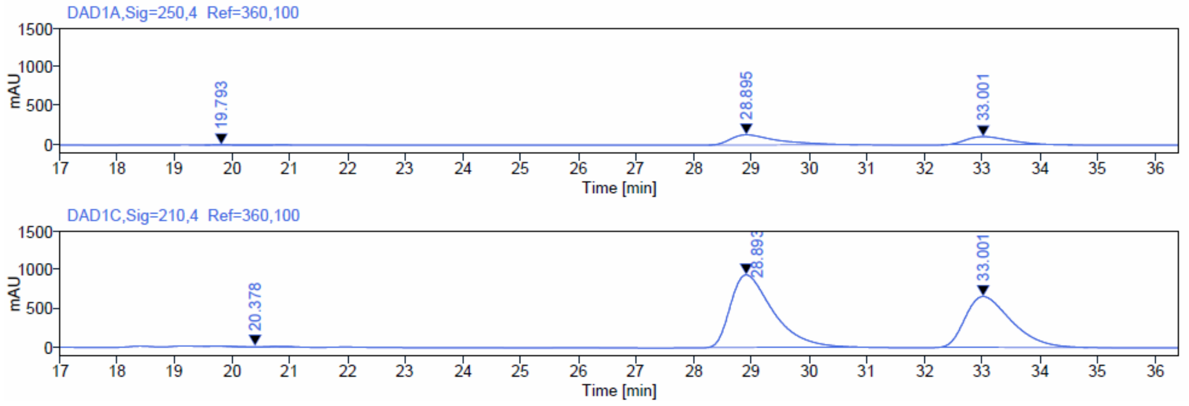

Signal: DAD1A,Sig=250,4 Ref=360,100

RT [min]	Type	Width [min]	Area	Height	Area%	Name
19.793	MM m	0.04	1.78	0.86	0.01	
28.895	MM m	0.83	7307.86	130.51	55.61	
33.001	MM m	0.84	5832.47	107.14	44.38	
		Sum	13142.10			

Signal: DAD1C,Sig=210,4 Ref=360,100

RT [min]	Type	Width [min]	Area	Height	Area%	Name
20.378	MM n	0.13	4.59	7.06	0.01	
28.893	MM m	0.77	47444.52	931.69	56.80	
33.001	MM m	0.85	36087.17	653.24	43.20	
		Sum	83536.29			

5g' – Enantioenriched sample

5g''

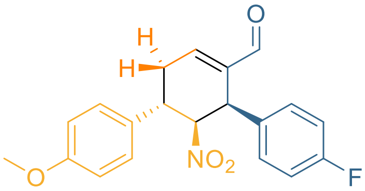

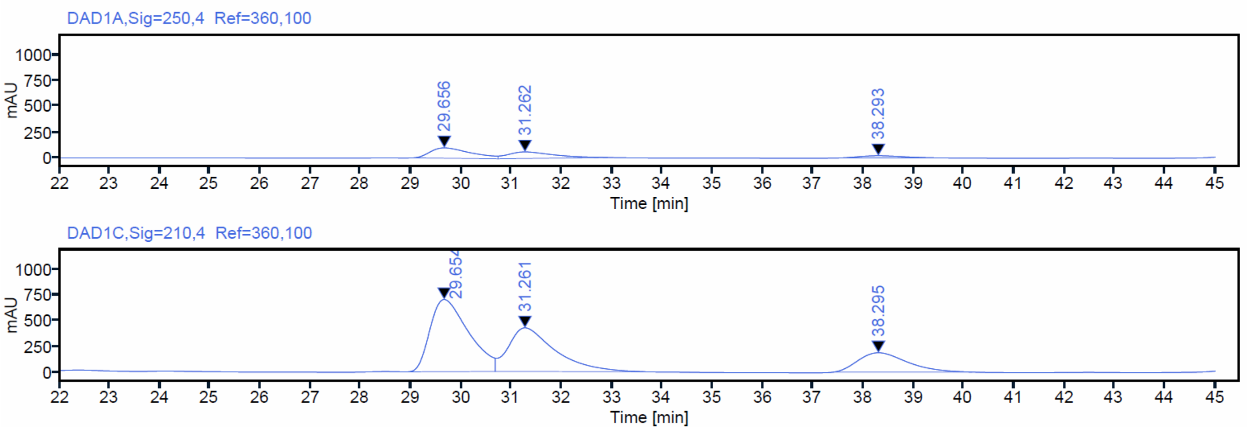

5g'' – Racemic sample

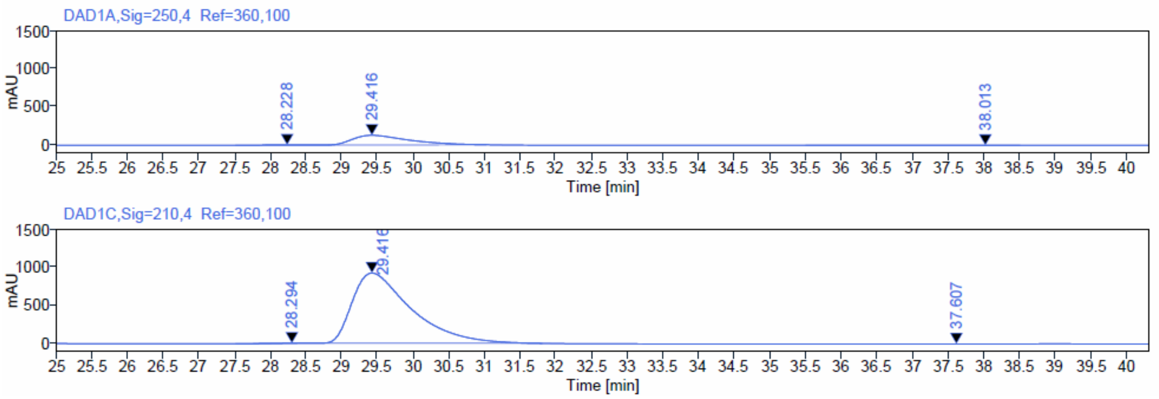

Signal: DAD1A,Sig=250,4 Ref=360,100

RT [min]	Type	Width [min]	Area	Height	Area%	Name
28.228	MM m	0.20	13.12	1.08	0.19	
29.416	MM m	0.82	6828.19	126.14	99.54	
38.013	MM n	1.03	18.19	0.21	0.27	
		Sum	6859.50			

Signal: DAD1C,Sig=210,4 Ref=360,100

RT [min]	Type	Width [min]	Area	Height	Area%	Name
28.294	MM m	0.24	22.46	1.56	0.04	
29.416	MM m	0.85	52631.49	921.00	99.69	
37.607	MM n	1.35	142.18	1.24	0.27	
		Sum	52796.13			

5g'' – Enantioenriched sample

5h’

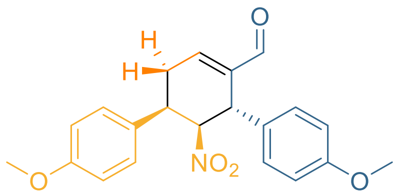

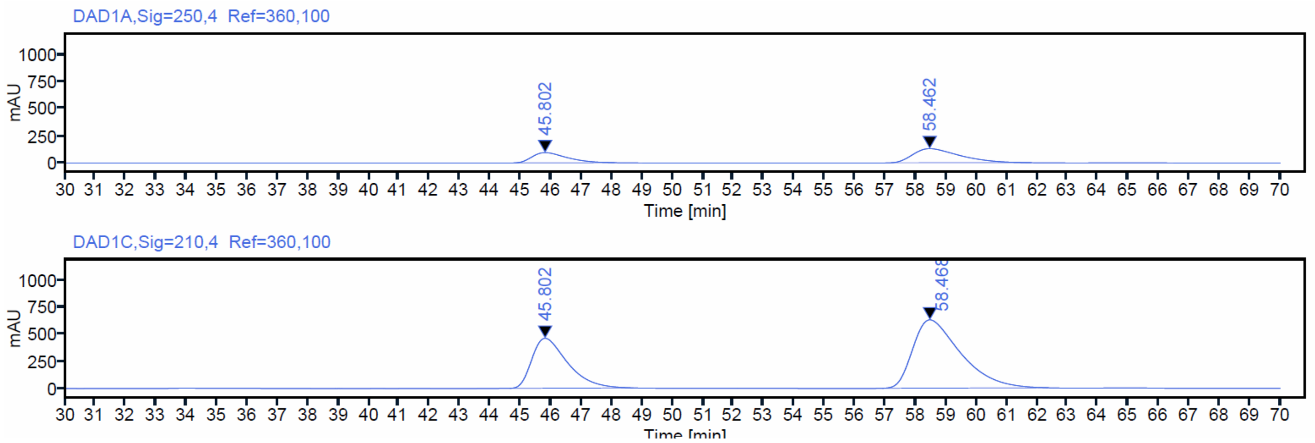

5h’ – Racemic sample

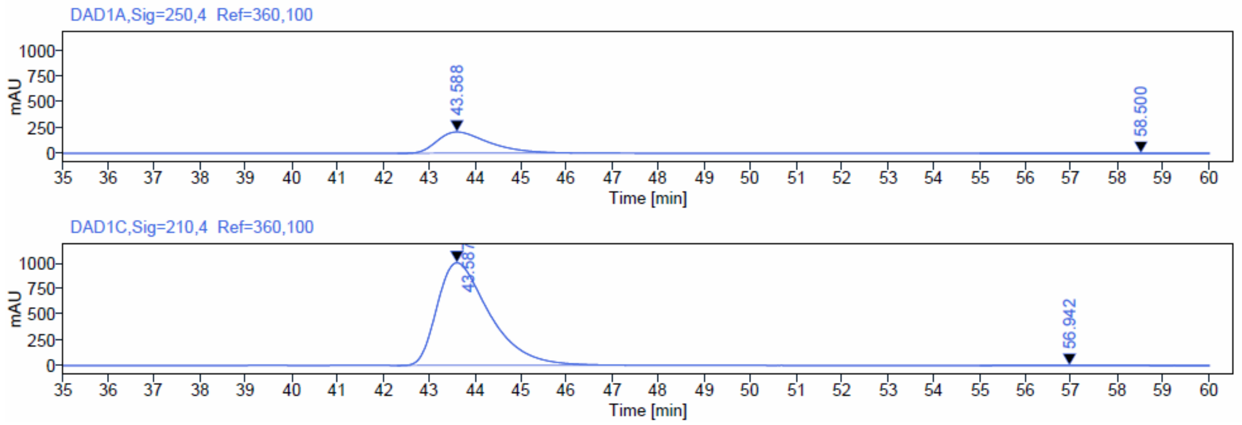

Signal: DAD1A,Sig=250,4 Ref=360,100

RT [min]	Type	Width [min]	Area	Height	Area%	Name
43.588	BM m	1.18	16173.44	208.34	99.85	
58.500	MM m	1.87	24.02	0.15	0.15	
		Sum	16197.45			

Signal: DAD1C,Sig=210,4 Ref=360,100

RT [min]	Type	Width [min]	Area	Height	Area%	Name
43.587	BM m	1.20	79886.47	1007.89	99.90	
56.942	MM m	1.69	81.35	0.56	0.10	
		Sum	79967.82			

5h’ – Enantioenriched sample

5h''

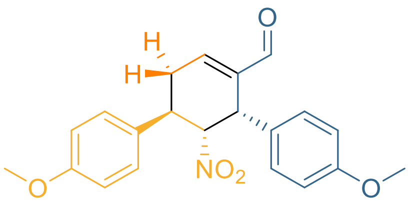

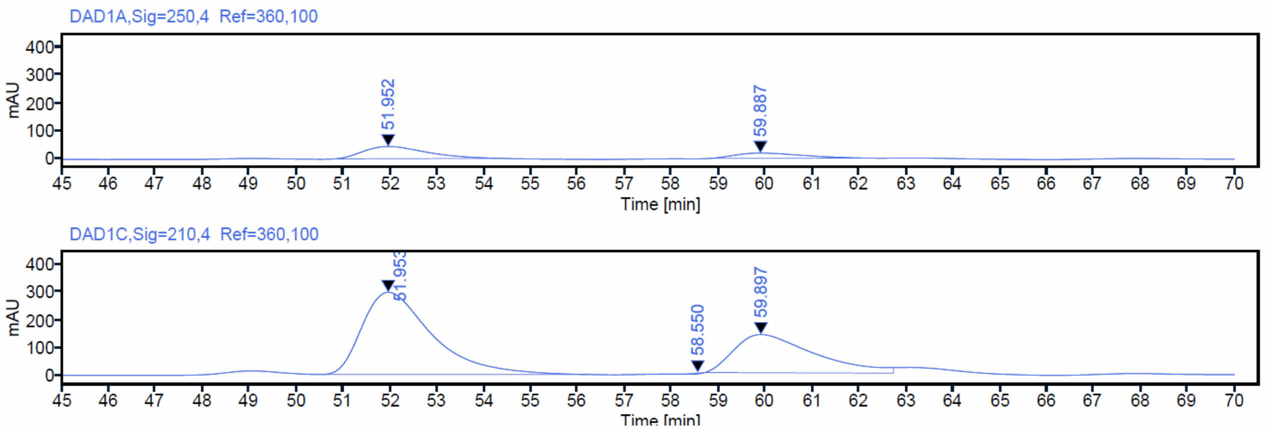

5h'' – Racemic sample

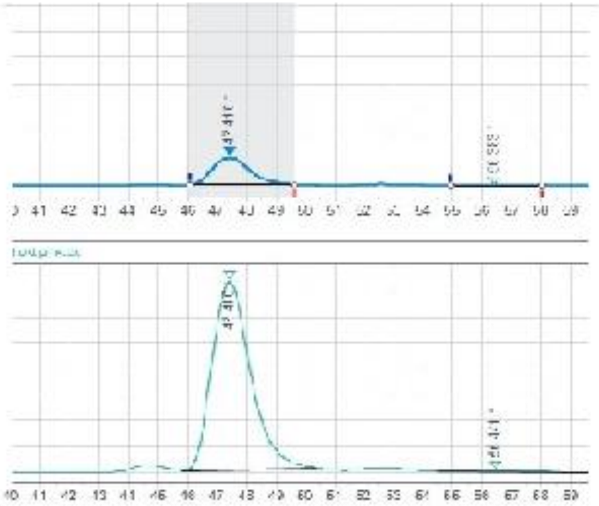

Signal: DAD1A,Sig=250,4 Ref=360,100

RT [min]	Type	Width [min]	Area	Height	Area%	Name
47.410	MM m	1.37	9719.53	110.51	99.95	
59.850	MM n	1.16	5.29	0.05	0.05	
		Sum	9724.82			

Signal: DAD1C,Sig=210,4 Ref=360,100

RT [min]	Type	Width [min]	Area	Height	Area%	Name
47.410	MM m	1.41	66247.48	727.78	99.54	
56.441	MM m	1.06	306.58	3.40	0.46	
		Sum	66554.06			

5h'' – Enantioenriched sample

5i’

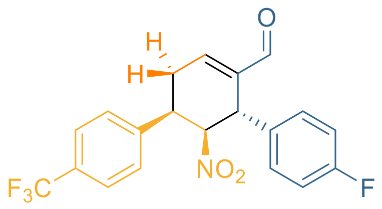

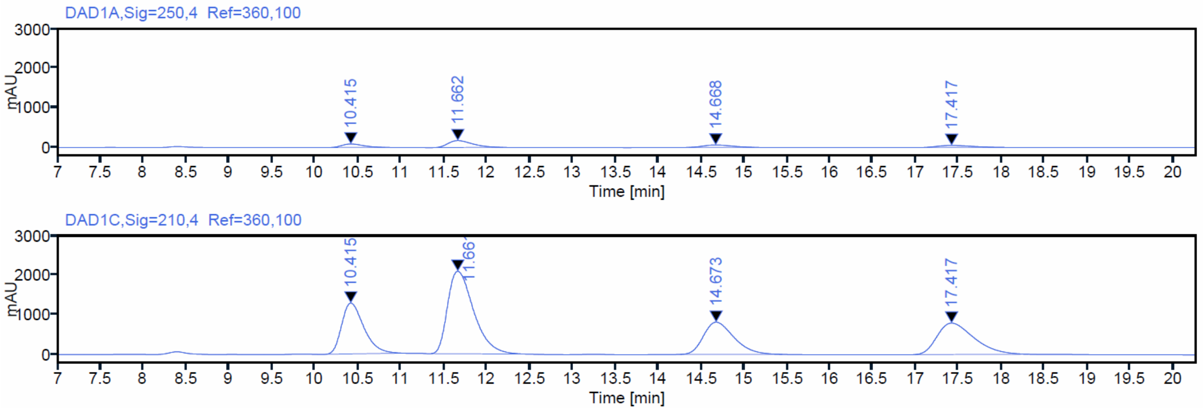

5i’ – Racemic sample

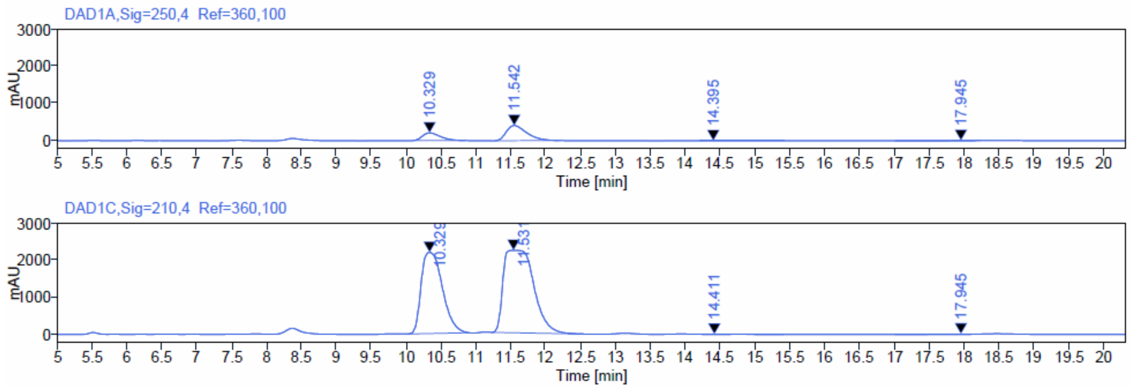

Signal: DAD1A,Sig=250,4 Ref=360,100

RT [min]	Type	Width [min]	Area	Height	Area%	Name
10.329	MM m	0.27	3630.48	205.74	30.27	
11.542	MM m	0.31	8229.88	405.87	68.61	
14.395	MM n	0.16	2.30	0.18	0.02	
17.945	MM n	0.67	132.23	3.30	1.10	
		Sum	11994.89			

Signal: DAD1C,Sig=210,4 Ref=360,100

RT [min]	Type	Width [min]	Area	Height	Area%	Name
10.329	MM m	0.33	46062.44	2180.57	40.63	
11.531	MM m	0.37	67041.17	2213.72	59.14	
14.411	MM n	0.23	35.99	2.66	0.03	
17.945	MM n	0.55	223.37	6.77	0.20	
		Sum	113362.97			

5i’ – Enantioenriched sample

5i''

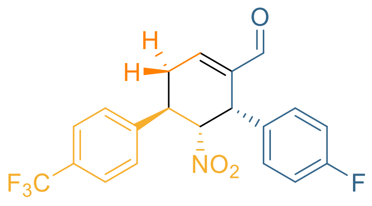

5i

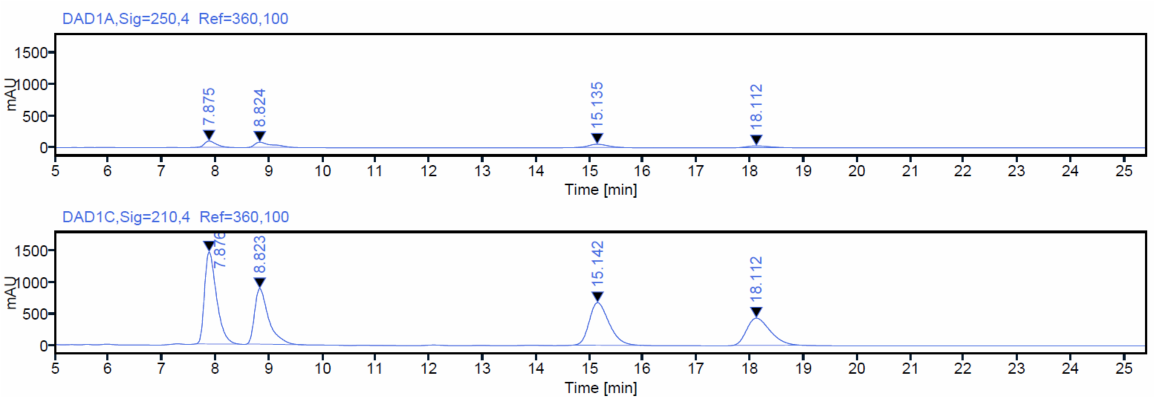

5i'' – Racemic sample

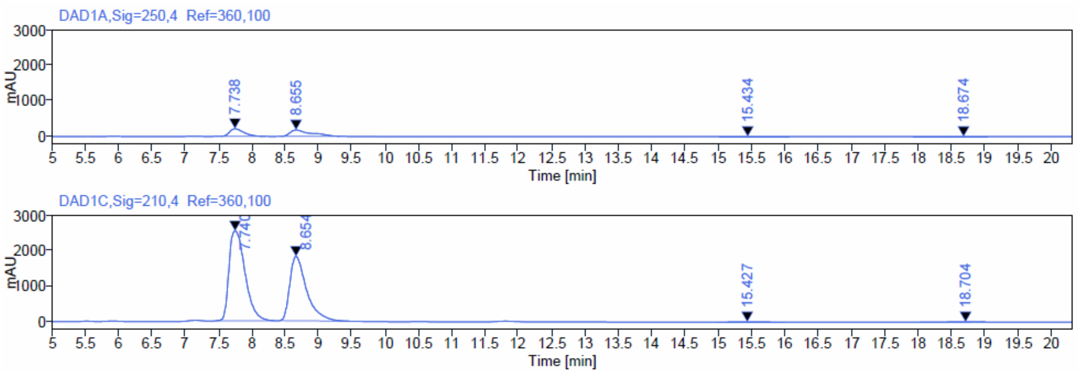

Signal: DAD1A,Sig=250,4 Ref=360,100

RT [min]	Type	Width [min]	Area	Height	Area%	Name
7.738	MM m	0.22	3108.90	212.79	44.40	
8.655	MM m	0.31	3880.17	177.77	55.41	
15.434	MM m	0.28	7.13	0.30	0.10	
18.674	MM m	0.31	6.26	0.25	0.09	
		Sum	7002.45			

Signal: DAD1C,Sig=210,4 Ref=360,100

RT [min]	Type	Width [min]	Area	Height	Area%	Name
7.740	MM m	0.26	42136.29	2521.36	56.94	
8.654	MM m	0.27	31735.90	1808.05	42.89	
15.427	MM m	0.36	78.44	3.22	0.11	
18.704	MM m	0.31	47.46	2.11	0.06	
		Sum	73998.09			

5i'' – Enantioenriched sample

5j’

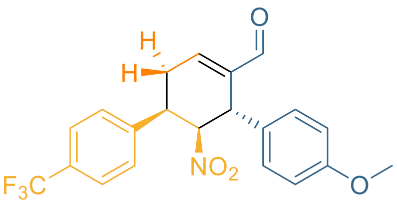

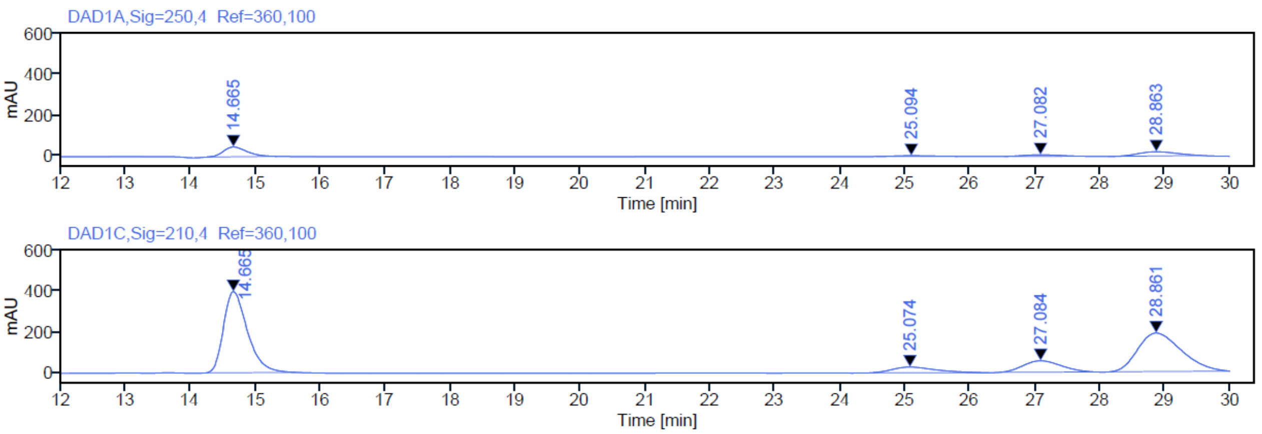

5j’ - Racemic sample

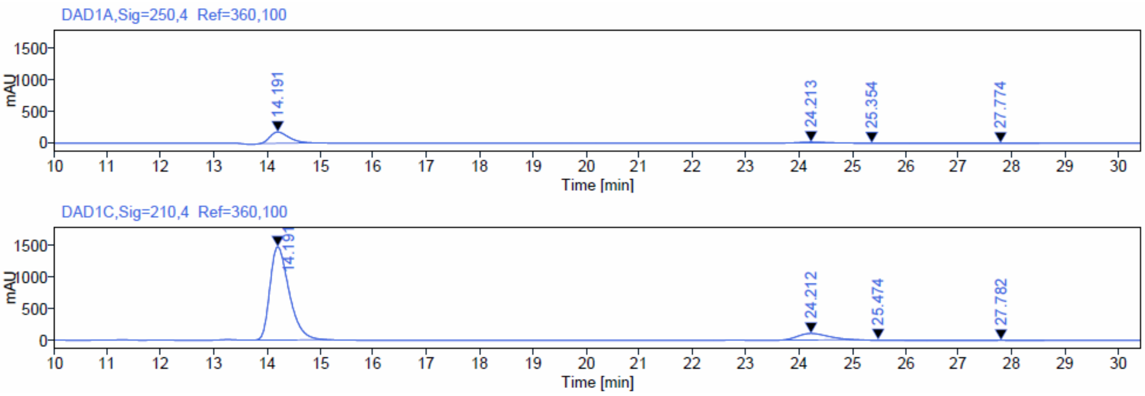

Signal: DAD1A,Sig=250,4 Ref=360,100

RT [min]	Type	Width [min]	Area	Height	Area%	Name
14.191	MM m	0.38	4414.69	179.51	91.98	
24.213	MM m	0.50	342.19	11.39	7.13	
25.354	MM n	0.65	28.39	0.73	0.59	
27.774	MM m	0.56	14.29	0.30	0.30	
		Sum	4799.56			

Signal: DAD1C,Sig=210,4 Ref=360,100

RT [min]	Type	Width [min]	Area	Height	Area%	Name
14.191	MM m	0.39	37107.66	1477.53	89.28	
24.212	MM m	0.63	4087.80	102.87	9.84	
25.474	MM n	0.81	222.17	4.55	0.53	
27.782	MM m	0.60	146.16	2.90	0.35	
		Sum	41563.79			

5j’ – Enantioenriched sample

5j''

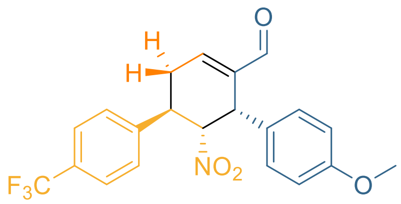

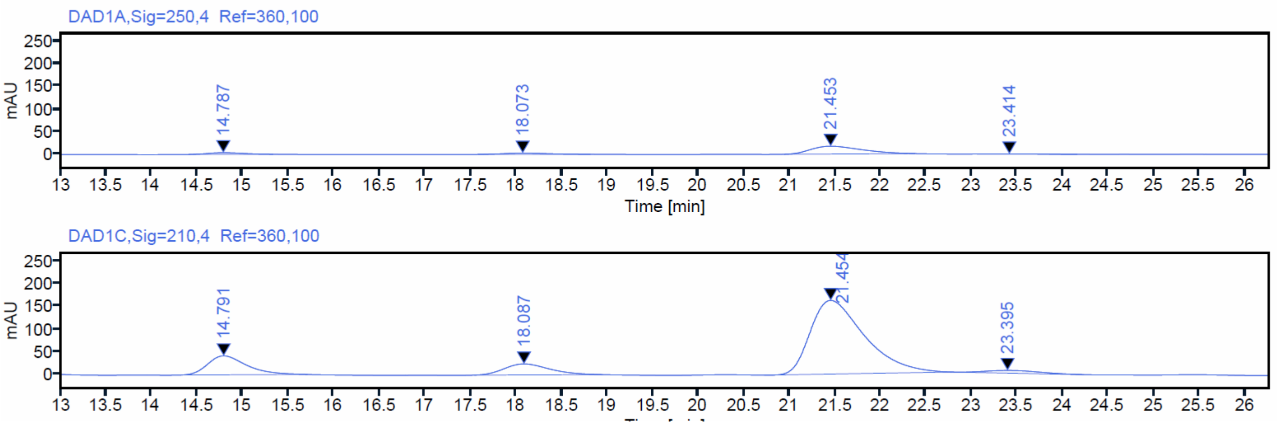

5j'' – Racemic sample

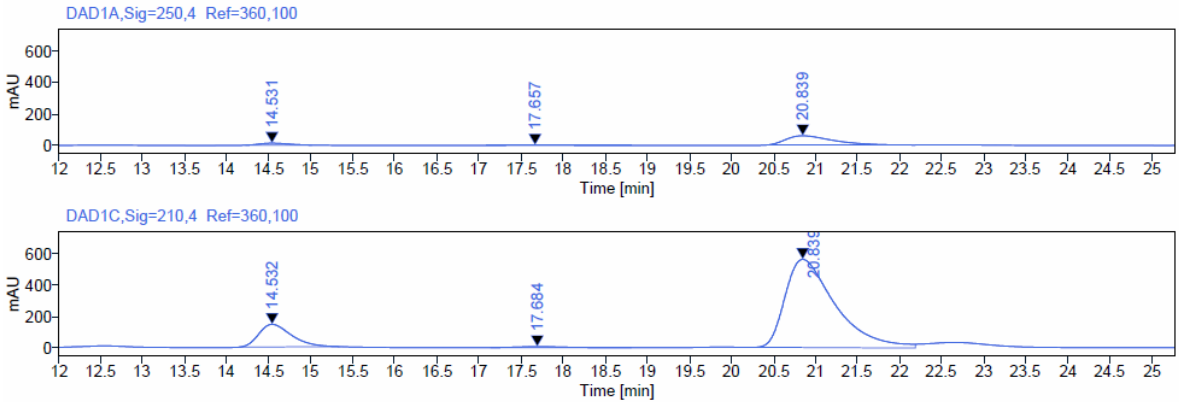

Signal: DAD1A,Sig=250,4 Ref=360,100

RT [min]	Type	Width [min]	Area	Height	Area%	Name
14.531	MM m	0.37	305.36	12.95	11.81	
17.657	MM m	0.50	82.12	2.01	3.18	
20.839	MM m	0.57	2198.33	58.39	85.01	
		Sum	2585.82			

Signal: DAD1C,Sig=210,4 Ref=360,100

RT [min]	Type	Width [min]	Area	Height	Area%	Name
14.532	MM m	0.41	3919.63	145.64	14.19	
17.684	MM m	0.50	256.33	6.48	0.93	
20.839	MM m	0.62	23447.62	567.40	84.88	
		Sum	27623.58			

5j'' – Enantioenriched sample

5. References

[1] G. Giorgianni, V. Nori, A. Baschieri, L. Palombi, A. Carlone, *Catalysts* **2020**, *10*, 1296-1302.

[2] D. Enders, M. Jeanty, J. W. Bats, *Synlett*, **2009**, *19*, 3175 – 3178.
